# Biochemical characterization of the two novel mgCas12a proteins from the human gut metagenome

**DOI:** 10.1038/s41598-022-25227-w

**Published:** 2022-12-02

**Authors:** Han Seong Kim, Dong-wook Kim, Sungjin Kim, Sunghwa Choe

**Affiliations:** 1 School of Biological Sciences, College of Natural Sciences, Seoul National University, Seoul, 08826 Korea; 2 G+FLAS Life Sciences, 123 Uiryodanji-gil, Osong-eup, Cheongju-si, 28160 Korea; 3 Naturegenic Inc., 1281 Win Hentschel Boulevard, West Lafayette, IN 47906 USA

**Keywords:** Biochemistry, Biological techniques, Biotechnology, Cell biology

## Abstract

CRISPR/Cas9 and Cas12a belonging to the Class II CRISPR system are characterized by a single-component effector protein. Despite unique features of Cas12a like DNA cleavage with 5′ staggered ends and a single crRNA, Cas12a has not been adopted in biotechnological applications to the similar extent as Cas9. To better understand the CRISPR/Cas12 systems, we selected two candidates, designated mgCas12a-1 and mgCas12a-2, from an analysis of the human microbiome metagenome (mg) and provided biochemical characterization. These new Cas12a proteins shared about 37% identity in amino acid sequences and shared the same direct repeat sequences in the crRNA with FnCas12a from *Francisella novicida*. The purification yield of the recombinant proteins was up to 3.6-fold greater than that of FnCas12a. In cell-free DNA cleavage assays, both mgCas12a proteins showed the higher cleavage efficiencies when Mn^2+^ was provided with KCl (< 100 mM) than tested other divalent ions. They were able to tolerate ranges of pH points and temperature, and showed the highest cleavage efficiencies at pH 8.0 and 50 °C. In addition, mgCas12a proteins showed 51% less crRNA-independent and 56% less crRNA-dependent non-specific nuclease activity upon prolonged incubation than did FnCas12a. Considering their greater yield in protein preparation and reduced non-specific nuclease activity, our findings may expedite the use of Cas12a especially when genome editing needs to be practiced with the form of ribonucleoproteins.

## Introduction

Clustered regularly interspaced short palindromic repeats (CRISPR) and CRISPR-associated (Cas) proteins comprise a bacterial and archaebacterial adaptive immune system against mobile genetic elements from phages or conjugating bacteria^1–3^. Cas9 and Cas12a belong to the class 2 CRISPR system and consist of a single effector Cas endonuclease that can be guided by a single RNA to cleave specific target DNA sequences^4,5^. Despite their similarities, they differ in that type II Cas9 from *Streptococcus pyogenes* Cas9 (SpyCas9) recognizes the protospacer adjacent motif (PAM) 5′-NGG-3′ and requires both a *trans*-activating CRISPR RNA (tracrRNA) and a CRISPR RNA (crRNA), which are processed by the host RNase III^6^. Successful enzymatic cleavage by SpyCas9 generates blunt-ended DNA double-strand breaks (DSBs) via its HNH and RuvC domains^7,8^. By contrast, type V Cas12a from *Acidaminococcus* sp. (AsCas12a) recognizes the PAM 5′-TTTV-3′, utilizes only a crRNA, and produces 5′ staggered-ended DSBs via the RuvC domain^9,10^. Studies have focused on improving the fidelity while reducing the off-target effects of Cas12a^11–13^ and Cas9^14–16^, and on developing their applications as base editors^17,18^, prime editors^19^, transcriptional regulators^20–23^, and nucleic acid detectors^24–27^.

Of the many members of the Cas12a protein family, those identified from *Acidaminococcus* sp. (AsCas12a), *Francisella novicida* (FnCas12a), and *Lachnospiraceae bacterium* (LbCas12a) are actively in use. Here, we report two new members of Cas12a protein family identified from a survey of the human metagenome, mgCas12a-1 and mgCas12a-2 (accession numbers CDYX01038443.1 and CDZH01035208.1, respectively) and their enzymatic characteristics including crRNA and divalent metal ion compatibility, salt, pH and temperature-dependent target DNA cleavage activities.

Cas12a possesses a unique feature called, “collateral DNase activity”. Activated by Cas12a ribonucleoprotein (RNP) cleaving a target DNA, this non-specific DNase activity degrades surrounding DNA fragments. Researchers recognized this unique characteristic of Cas12a and developed nucleic acid detection methods. In this study, we aimed to further understand various non-specific DNase activities including collateral DNase activity, by comparing such activities among five different Cas12a orthologs. Through cell-free in vitro DNA cleavage assays, we found that both mgCas12a-1 and 2 possess more than 50% reduced non-specific DNase activities than FnCas12a did. In addition, we have also detected remaining non-specific DNase activities even in deactivated version of the mgCas12a proteins.

## Results and discussion

### Discovery of 11 new Cas12a candidates from a human gut microbiome database

**Figure 1:**
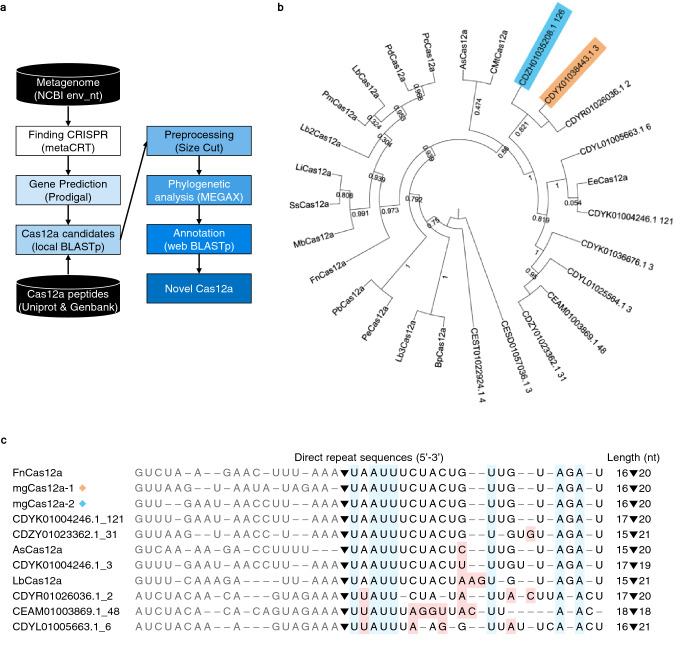
Discovery of Cas12a candidates from the human gut microbiome. (**a**) Pipeline used to search for and classify new putative Cas12a proteins. CRISPR arrays were identified from GenBank metagenome data; putative Cas proteins were then sorted based on amino acid length and their evolutionary relationships with 16 previously characterized Cas12a proteins. (**b**) Phylogenetic tree of 27 Cas12a variants. The tree consists of 16 previously known Cas12a proteins and 11 Cas12a candidates extracted from the human metagenome. The tree was constructed with iTOL v6; bootstrap values (1000 ×) are displayed at each node. MgCas12a-1 and mgCas12a-2 are highlighted in orange and blue, respectively. (**c**) Alignment of direct repeat sequences associated with 8 novel Cas12a candidates and 3 previously known Cas12a orthologs. The sequences removed during crRNA maturation are shown in gray. MgCas12a-1 and mgCas12a-2 are indicated with orange and blue diamonds, respectively. Black triangle indicates putative cleavage site during crRNA processing. Consensus sequences and mismatching 5′ handle sequences are highlighted in blue and pink, respectively.

To discover new members of the Cas12a protein family, we built a pipeline that searches and sorts out new candidates from the National Center for Biotechnological Information (NCBI) human metagenome database. Using this search pipeline (Fig. 1a), we initially discovered 42,045 CRISPR array sequences from the NCBI env_nt metagenome database^28^. We identified all proteins encoded by genes located within 15 kb of the CRISPR arrays and saved them in a local database for a BLASTp search. We then queried the local database using 16 known Cas12a proteins as baits (Supplementary Table 1), which led to the identification of 11 new Cas12a candidates (Fig. 1b and Supplementary Table 2). We first excluded the three entries, CDYL01025564.1_3, CESD01057036.1_3, and CEST01022924.1_4, due to unclear premature-crRNA processing sequences and lack of direct repeat sequences. The remaining eight had direct repeat sequences with a conserved crRNA processing region and 5′ handle sequences (Fig. 1c). Interestingly, the direct repeat sequences were conserved among FnCas12a, mgCas12a-1 (CDYX01038443.1_3), mgCas12a-2 (CDZH01035208.1_126), and CDYK01004246.1_121.

The candidates CDZY01023362.1_31 and CEAM01003869.1_48 were identical in their protein sequence and length, as were CDYR01026036.1_2 and CDYX01038443.1_3; however, they carried different spacer sequences, suggesting that they may have originated from similar species but from distinct individuals that experienced a different infection history. Another two candidates, CDYK01004246.1_121 and CDYL01005663.1_6, clustered with *Eubacterium eligens*^29^ in a phylogenetic tree with 98% sequence identity (Fig. 1b and Supplementary Table 3). We performed a multiple amino acid sequence alignment of 16 previously reported Cas12a proteins and the 11 new candidates from this study and selected two final Cas12a candidates, CDYX01038443.1_3 and CDZH01035208.1_126, for further characterization. We designated these two proteins mgCas12a-1 and mgCas12a-2, with the prefix “mg” meaning “metagenome.” To assess the functionality of these candidates as members of the Cas12a family, we compared their predicted three-dimensional (3D) structure against that of FnCas12a (PDB ID: 5NG6) over the amino acid residues participating in DNA–RNA interaction^30^ and observed polymorphisms between the DNA–RNA interacting residues in both mgCas12a candidates relative to FnCas12a.

### mgCas12a remains functional despite the absence of adaptor Cas proteins

**Figure 2:**
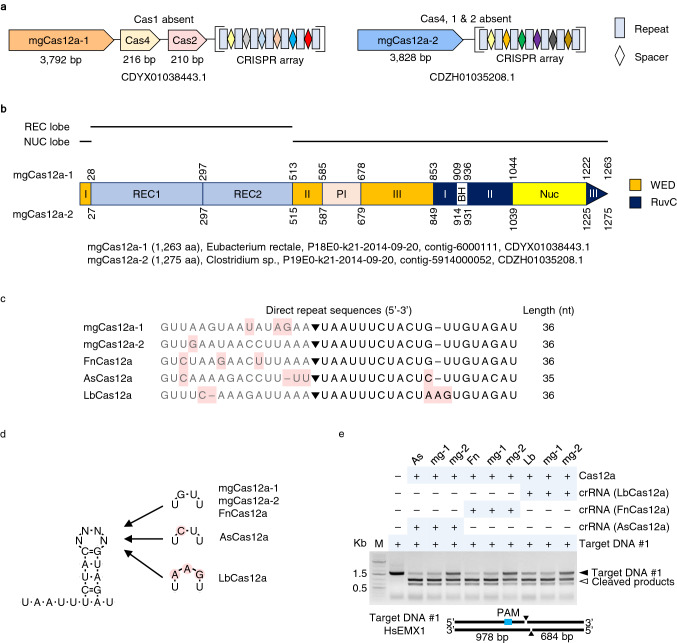
Identification of a functional crRNA for mgCas12a. (**a**) CRISPR loci of *mgCas12a-1* and *mgCas12a-2*. Unlike other class 2 type V-A CRISPR Cas12a systems, no *Cas1* sequence was found at the *mgCas12a-1* locus, while no gene encoding an adaptor Cas protein was found at the mgCa12a-2 locus. Seven repeats and six spacers were identified within the CRISPR array of each *mgCas12a* locus. (**b**) Domain structure of mgCas12a-1 and mgCas12a-2. Protein domains were predicted using structure-based alignments with the amino acid sequences of AsCas12a, FnCas12a, and LbCas12a. Amino acid numbers between each domain are shown between each domain; the putative gut bacterial species, sequence identifier, and GenBank accession numbers are given below the diagrams. WED, wedge domain; REC, recognition domain; PI, PAM interaction domain; RuvC, RuvC nuclease domain; BH, bridge helix domain; Nuc, nuclease domain. (**c**) Alignment of direct repeat sequences and predicted 5′ handle structures from the five *Cas12a* orthologous loci. The sequences removed during crRNA maturation are shown in gray. Triangle, putative cleavage site during crRNA processing. Mismatching 5′ handle sequences are highlighted in pink. (**d**) 5′ handle structure of mature crRNA from *Cas12a*. (**e**) Compatibility of mgCas12a with various crRNA forms for target DNA cleavage. Three types of crRNA (from the *AsCas12a*, *FnCas12a*, or *LbCas12a* loci) were used to test mgCas12a RNP-mediated target DNA cleavage activity. A partial amplicon of *HsEMX1* was used as target DNA #1. Filled arrowhead: Target DNA, 1,662 bp; unfilled arrowhead: Cleaved products, 978 bp + 684 bp. Original gels are presented in Supplementary Fig. 3.

In class 2 type V-A CRISPR/Cas12a systems, adaptor Cas modules including *Cas4*, *Cas1*, and *Cas2* reside between *Cas12a* and the CRISPR array^9,31,32^. We identified *Cas2* and *Cas4* genes, but not *Cas1*, at the *mgCas12a-1* locus, while none of the adaptor Cas proteins appeared to be encoded by the *mgCas12a-2* locus. The CRISPR arrays consisted of seven repeats and six spacers for both *mgCas12a* loci (Fig. 2a).

Multiple amino acid sequence alignment of mgCas12a with 16 known Cas12a orthologs suggested that both mgCas12a proteins share an identical domain architecture, with a RuvC domain as their endonuclease domain^9,10^ (Fig. 2b and Supplementary Fig. 1). We next aligned the direct repeat sequences in the CRISPR arrays of the *mgCas12a-1* and *mgCas12a-2* loci, which revealed that the 5′ handle of mgCas12a is identical to that of FnCas12a, differing only by a few nucleotides at the loop region relative to AsCas12a and LbCas12a^9^ (Fig. 2c,d). To ascertain whether the new mgCas12a candidates are functional and to identify the most effective crRNA structure for target DNA cleavage activity, we assembled RNP complexes with each crRNA and mgCas12a and performed in vitro DNA cleavage assays against a target DNA amplified from the human gene *Empty spiracles homeobox 1* (*EMX1*) (Fig. 2e and Supplementary Fig. 2). Both mgCas12a proteins complexed with all three crRNA handle structures successfully and cleaved the target DNA in vitro. Judging by the intensity of the uncleaved DNA, mgCas12a-1 cut more DNA than mgCas12a-2.

To test if the mgCas12a accept the PAM sequence with variation, 5′-TTTV-3′ PAM sequence was modified at their 5’terminal base and performed in vitro cleavage assay (Supplementary Fig. 3). When the substrate DNA with different PAM sequences were subjected with the mgCas12a-1 complexed with the HsCCR5_crRNA3, 5′-terminal base of the TTTV was well tolerated. The percentage of the cleaved DNA when treated with 4 pmol of mgCas12a-1, the cleavage efficiency was 89, 88, 94, and 99% for the GTTA, ATTA, CTTA, and TTTA PAM, respectively. Pyrimidine nucleotides at the 5’ terminal of the PAM such as CTTA and TTTA is slightly preferred over purine.

### Mn^2+^ and low salt concentration enhance the activity of the new mgCas12a candidates

**Figure 3:**
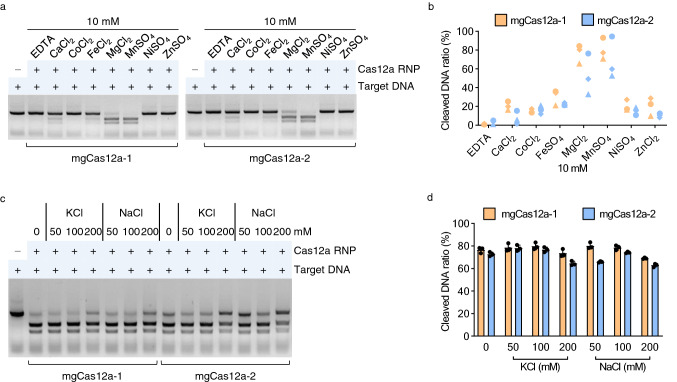
In vitro characterization of the mgCas12a variants in metal ion and salt variations. (**a**) Metal ion-dependent in vitro DNA cleavage activity. 10 mM of EDTA, CaCl_2_, CoCl_2_, FeCl_2_, MgCl_2_, MnSO_4_, NiSO_4_ and ZnSO_4_ were used as cofactors for target DNA cleavage. Original gels are presented in Supplementary Fig. 4. (**b**) The metal ion-dependent cleavage efficiency of mgCas12a was measured with the assay shown in panel (A) and Supplementary Fig. 4. Three different target DNAs were used. Circle: Target DNA #1; Diamond: Target DNA #2; Triangle: Target DNA #3 (*n* = 1 for each target DNA). (**c**) Salt-dependent in vitro DNA cleavage activity. Cleavage assays under a gradient of KCl and NaCl from 50 to 200 mM were performed using target DNA #1. Original gels are presented in Supplementary Fig. 5. (**d**) The cleavage efficiency of mgCas12a under different salt concentrations was measured with the assay shown in panel (C) and Supplementary Fig. 5. The error bars represent standard deviations (*n* = 3).

To identify the cofactors required for full enzymatic activity, we tested the effect of various metal ions on DNA cleavage activity of the new mgCas12a candidates. We incubated each RNP complex with 10 mM CaCl_2_, CoCl_2_, FeCl_2_, MgCl_2_, MnSO_4_, NiSO_4_, or ZnSO_4_ or with distilled water or 10 mM EDTA as negative controls (Fig. 3a and Supplementary Fig. 4). In the presence of MnSO_4_, mgCas12a-1 and mgCas12a-2 showed an average cleavage efficiency of 80% and 69%, respectively. MgCl_2_ also supported cleavage activity, with 77% (mgCas12a-1) and 53% (mgCas12a-2) cleavage efficiency. Several other metal ions, such as CaCl_2_ and FeCl_2_, resulted in only marginal activity (Fig. 3b).

We also tested varying concentrations (50, 100, and 200 mM) of potassium or sodium. In the KCl-based assays, we observed the highest cleavage efficiency for mgCas12a-1 (80% cleavage of the substrate DNA) in the presence of 100 mM KCl and for mgCas12a-2 (78%) with 50 mM KCl (Fig. 3c and Supplementary Fig. 5). When using NaCl, mgCas12a-1 cleaved up to 80% of the target DNA in the presence of 50 mM NaCl, and mgCas12a-2 cleaved up to 74% with 100 mM NaCl. Both mgCas12a RNPs showed their lowest cleavage activity at concentrations of 200 mM for both salts (Fig. 3d).

### Wide spectrum of pH and temperature tolerance

**Figure 4:**
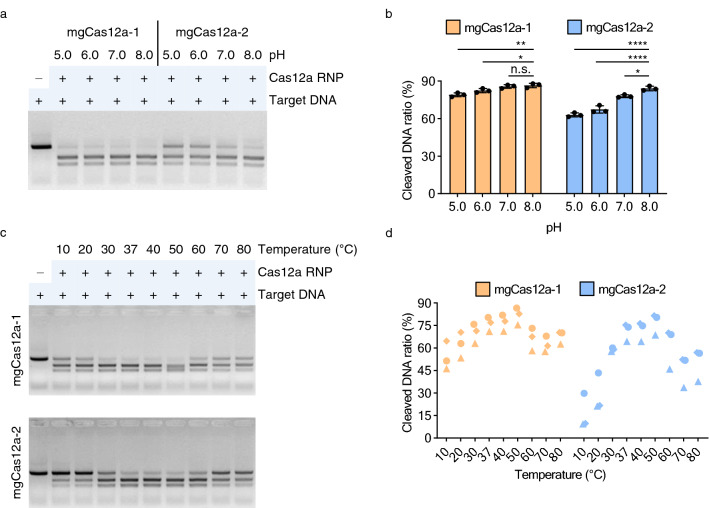
In vitro characterization of the mgCas12a variants in pH and temperature variations. (**a**) pH-dependent in vitro DNA cleavage activity. Cleavage assays under four different pH points from 5.0 to 8.0 were performed using target DNA #1. Original gels are presented in Supplementary Fig. 6. (**b**) The cleavage efficiency of mgCas12a under different pH points was measured with the assay shown in panel (A) and Supplementary Fig. 6. Error bars represent standard deviations (*n* = 3). Significance was analyzed using a one-way ANOVA followed by Dunnett’s multiple comparison test (*p < 0.05; **p < 0.01; ***p < 0.001; ****p < 0.0001; n.s. not significant). (**c**) Temperature-dependent in vitro DNA cleavage activity. Target DNA and mgCas12a RNP complex were incubated under a gradient of temperature from 10 to 80 °C for 30 min. Original gels are presented in Supplementary Fig. 7. (**d**) The temperature-dependent cleavage efficiency of mgCas12a was measured with the assay shown in panel (C) and Supplementary Fig. 7. Three different target DNAs were used. Circle: Target DNA #1; Diamond: Target DNA #2; Triangle: Target DNA #3 (*n* = 1 for each target DNA).

After metal ions and salt concentrations, we determined the effect of pH on mgCas12a activity. The calculated pI values for mgCas12a-1 and mgCas12a-2 were 6.91 and 6.15, respectively. We thus tested cleavage efficiency for each mgCas12a RNP complex at pH values 5.0, 6.0, 7.0, and 8.0 and obtained the highest activity at pH 8.0 (Fig. 4a and Supplementary Fig. 6). The activity of mgCas12a-2 at pH 8.0 was about 30% higher than that at pH 5.0, whereas the activity of mgCas12a-1 was only about 10% higher at pH 8.0 versus pH 5.0, suggesting that the structure of mgCas12a-2 may be more sensitive to changes in pH (Fig. 4b).

We also tested nine different temperatures (10, 20, 30, 37, 40, 50, 60, 70, and 80 °C) for in vitro cleavage assays. We incubated pre-assembled mgCas12a RNP complexes and target DNAs at each temperature. Over 60% of the substrate DNA was cleaved by mgCas12a-1 at temperatures above 20 °C, suggesting a broad spectrum of temperature tolerance (Fig. 4c and Supplementary Fig. 7). For mgCas12a-2, several temperatures (30, 37, 40, 50, and 60 °C) supported DNA cleavage above 60% efficiency. In addition, the cleavage efficiency exhibited by mgCas12a-1 in vitro increased 1.5-fold from 10 to 50 °C, while that of mgCas12a-2 increased 4.8-fold over the same temperature range, suggesting that mgCas12a-1 is more tolerant to changes in temperature (Fig. 4d).

### Prolonged incubation results in collateral DNase activity

**Figure 5:**
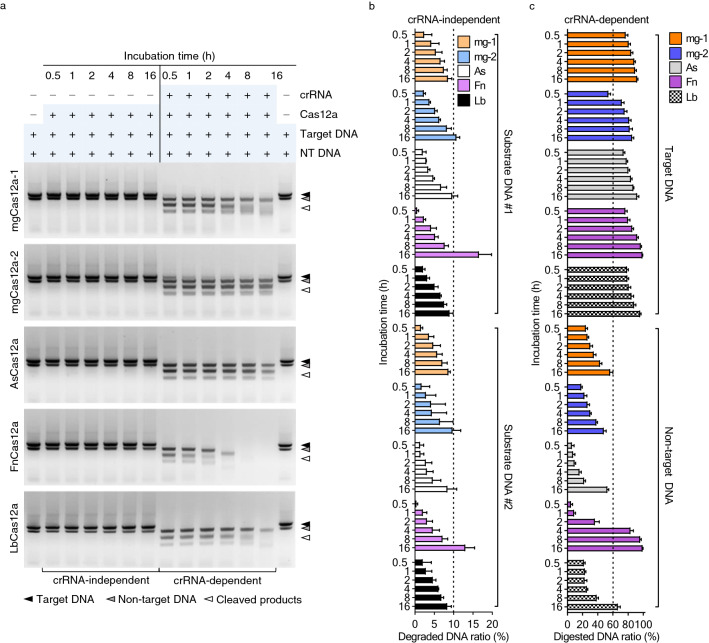
Collateral DNase activity is preserved in mgCas12a proteins. (**a**) Assessment of collateral dsDNase activity of the Cas12a protein family. Cas12a RNP complexes were incubated with target and non-target DNA at 37 °C from 30 min to 16 h. Original gels are presented in Supplementary Fig. 9. Filled arrowhead: Target DNA, 1662 bp; grey arrowhead: Non-target DNA, 1321 bp; unfilled arrowhead: Cleaved products, 978 bp + 684 bp. (**b**, **c**) Comparison of Cas12a-mediated RNA-independent (**b**) and RNA-dependent (**c**) random dsDNase activity on target DNA and non-target DNA. The intensity of digested and undigested target DNA and non-target DNA bands shown in (**a**) and Supplementary Fig. 9 were measured with ImageJ. Error bars represent standard deviation (*n* = 3).

Cas12a proteins show collateral DNase activity^24,33^, which is activated after successful cleavage of a target DNA sequence (Supplementary Fig. 8, right). We performed a time-course analysis from 30 min to 16 h of mgCas12a activity in an in vitro cleavage assay in the absence or presence of crRNA and with non-target DNA as substrate (Fig. 5a and Supplementary Fig. 9).

In the absence of crRNA, we detected a degradation level of less than 10% up to 16 h when the non-target substrate DNA was incubated with either of the two mgCas12a proteins (Fig. 5b). However, FnCas12a degraded about 17% of the substrate DNA under the same conditions. In the presence of crRNA, most Cas12a RNP complexes reached at least 70% in cleavage efficiency of their target DNA within 30 min, with mgCas12a-2 reaching 70% after 1 h (Fig. 5c). The non-target DNA substrate required more than 8 h to be degraded by 50% in the presence of each Cas12 RNP complex. The collateral DNase activity was relatively high in FnCas12a and low in our mgCas12a’s and AsCas12a.

### Substitution mutations in the RuvC domain of Cas12a preserve random DNase activity

**Figure 6:**
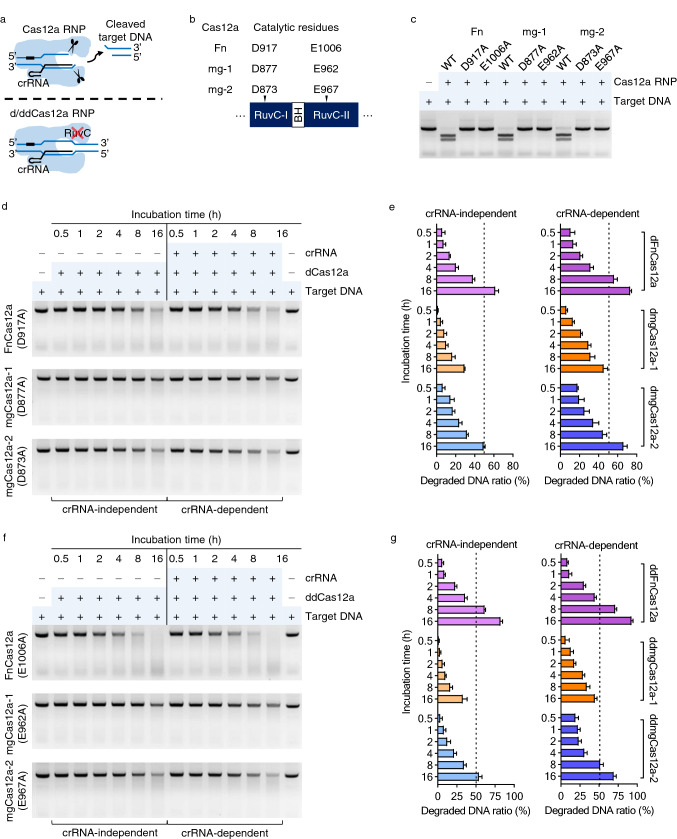
Catalytically inactive Cas12a mutants retain random DNase activity. (**a**) Schematic diagram illustrating cleavage of target DNA by the Cas12a RNP complex (top) but not by a catalytically inactive mutant Cas12a RNP complex (bottom). (**b**) Partial domain architecture of Cas12a proteins. Catalytic residues (marked with arrowheads) for each Cas12a protein are shown, which were predicted by multiple amino acid sequence alignment with Cas12a orthologs (Supplementary Fig. 1). RuvC, RuvC nuclease domain; BH, bridge helix domain. (**c**) Identification of the catalytic residues responsible for target DNA cleavage activity. Target DNA remained undigested when incubated with each mutant Cas12a RNP complex at 37 °C for 30 min. (**d**) CRISPR RNA-independent and RNA-dependent random DNase activity of dCas12a mutants. Target DNA was incubated with dCas12a mutants only or with dCas12a RNP complexes at 37 °C from 30 min to 16 h. Original gels are presented in Supplementary Fig. 10. (**e**) Average random DNase activity of dCas12a and dCas12a RNP complexes based on the assay shown in (**d**). The intensity of digested and undigested target DNA bands shown was measured with ImageJ. Error bars represent standard deviation (*n* = 3). (**f**) CRISPR RNA-independent and RNA-dependent random DNase activity of ddCas12a mutants. Target DNA was incubated with ddCas12a mutants only or with ddCas12a RNP complexes at 37 °C from 30 min to 16 h. Original gels are presented in Supplementary Fig. 11. (**g**) Average random DNase activity of dCas12a and ddCas12a RNP complexes based on the assay shown in (**f**). The intensity of digested and undigested target DNA bands shown was measured with ImageJ. Error bars represent standard deviation (*n* = 3).

The two amino acid residues D917 and E1006 of FnCas12a within the RuvC domain are responsible for cleaving target DNA^9^ (Fig. 6a). We identified the equivalent residues in mgCas12a proteins from a multiple sequence alignment (Supplementary Fig. 1). We determined that D877 and D873 in mgCas12a-1 and mgCas12a-2, respectively, correspond to D917 in FnCas12a, while E1006 in FnCas12a is equivalent to E962 and E967 in mgCas12a-1 and mgCas12a-2, respectively (Fig. 6b).

We generated variants of mgCas12a in which each residue potentially important for collateral DNase activity was replaced by alanine. We investigated whether these variants were catalytically inactive on target DNA by incubating RNP complexes consisting of the dCas12a or ddCas12a variants with target DNA for 30 min at 37 °C. Only the wild-type versions of FnCas12a and mgCas12a cleaved the target DNA, indicating that the mutants are catalytically inactive (Fig. 6c).

We then tested their collateral DNase activity. We repeated the time-course analysis presented above in Fig. 5, incubating each RuvC-deactivated mutant with the substrate DNA either alone or as an RNP complex with crRNA (Fig. 6d and Supplementary Fig. 10). The D917A mutant of FnCas12a failed to cleave DNA at the target site; however, this mutant did degrade the substrate DNA after at least 8 h of incubation regardless of the presence of crRNA. After 16 h, more than 80% of the substrate DNA had disappeared (Fig. 6e). By contrast, collateral DNase activity was much lower for the mutated mgCas12a proteins. Only the mgCas12a-2 RNA complex resulted in a 60% degradation of the target DNA after a 16-h incubation, regardless of the presence of crRNA, as seen with the FnCas12a variants (Fig. 6f,g, and Supplementary Fig. 11).

The random DNase activity detected in the Cas12a variants even in the absence of target DNA cleavage activity suggests that this collateral nuclease function is independent of the two residues in the active site of the RuvC domain. The WED III domain also carries out nuclease function^1^. In particular, residue K869 participates in acid–base hydrolysis of RNA when processing crRNA. In light of the facts that CRISPR/Cas9 requires an RNase function provided by the host to process the guide RNA and that Cas9 does not have collateral nuclease activity, it is tempting to propose that crRNA processing might be responsible for the non-specific nuclease activity observed during prolonged incubation in our experiments. Collateral DNase activity required longer incubations of at least 1 h and was favored in FnCas12a. A comparison of protein sequences among Cas12a proteins revealed a 40-amino acid insertion in front of the K869 residue in FnCas12a (Supplementary Fig. 1). The influence of this insertion on the 3D structure of the proteins and associated collateral DNase activity would be worth pursuing in future research.

### High purification yield of mgCas12a-1

Chimeric Cas12a proteins with the maltose-binding protein (MBP) at their N termini increases levels of protein production^34^. Although MBP may increase the yield of soluble protein, usage of this system requires additional protein purification steps to cleave and separate MBP from Cas12a, which would lead to lower final protein yields. We compared the purification levels of the five different Cas12a proteins (mgCas12a-1, mgCas12a-2, AsCas12a, FnCas12a, and LbCas12a) without any help of MBP in the bacterial (*Escherichia coli*) production system. Based on SDS-PAGE analysis of fractions purified on immobilized affinity chromatography columns and final Cas12a protein yield measurements, we estimated that mgCas12a-1 shows the highest yield, followed by AsCas12a, mgCas12a-2, LbCas12a, and FnCas12a (Supplementary Fig. 12b,c). Using the mgCas12a-1 could be advantageous especially when needing to perform genome editing in a RNP format.

### mgCas12a-1 successfully edit the CCR5 gene in a human cell

Our DNA in vitro cleavage assay exhibited that the assembled mgCas12a RNPs in reaction buffer does not need any other components when creating double strand breaks in the target DNA (Figs. 1, 2, 3, 4, 5, 6). In order to test if the RNP complex can edit the target DNA in human cells, we assembled the crRNA’s targeting the CCR5 gene and mgCas12a-1 effector protein, and transfected the RNP into human cell line HEK293T via electroporation. At 24 h after transfection, genomic DNA was prepared, and PCR was performed to amplify the DNA covering the target sites of crRNA1 and crRNA3. The 818 bp PCR products were subjected to digestion with T7 Endonuclease I (T7E1)^35^.

**Figure 7:**
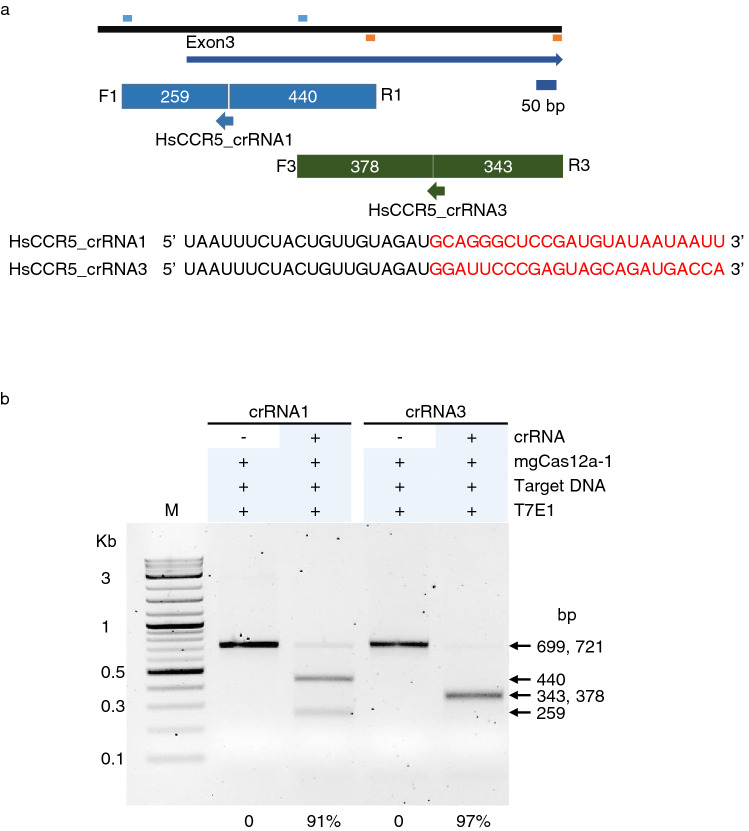
A mgCas12a-1 ribonucleoprotein successfully edits the CCR5 gene in HEK293 cells. (**a**) Schematic diagram illustrating target sites of the CCR5 gene. Solid lines indicate the locations for genomic DNA (top) and the exon 3 of the CCR5 gene. The forward and reverse primers are denoted on the top and bottom of the genomic DNA with short lines. Substrate DNA amplified with F1 and R1 primers is cut into 259 and 440 bp DNA when digested by mgCas12a-1-HsCCR5_crRNA1 enzyme. The 721 bp long DNA is cut into 378 and 343 bp DNA when cut by mgCas12a-1 with HsCCR5_crRNA3. The crRNA sequences for HsCCR5_crRNA1 and 3 are shown. Red-colored font is for spacer RNA. (**b**) T7E1 analysis of the PCR-amplified DNA. The PCR products amplified by using F1–R1 for crRNA1 and F3–R3 for crRNA3 are shown. When RNPs with each crRNA were treated with the HEK293T cells, T7E1 endonuclease successfully cut the DNA into two pieces as indicated. The editing efficiency measured by ImageJ software is indicated at the bottom.

The PCR-amplified DNA treated with mgCas12a effector protein only was not cleaved by the T7E1 (Fig. 7, lane second and fourth from left), whereas the DNAs amplified after treatment with functional RNPs consisting in the mgCas12a-1 effector protein and crRNA1 or 3 (Fig. 7, lane third and fifth from left) were cut into two pieces as expected by T7E1 endonuclease. Determined by the difference of the DNA intensity at the substrate position of 818 bp, RNPs with crRNA1 or crRNA3 edited the genomic DNA more than 90%.

In conclusion, our data suggest that the putative Cas12a sequences identified from the human gut microbiome databases encode functional Cas12a proteins working in vitro and in vivo. Many of the unique features attributable to our mgCas12a might expedite applicability of the Cas12a tools in human therapeutics as well as agricultural trait development.

## Materials and methods

### Searching for and defining new members of the Cas12a protein family from a metagenome database

Metagenome nucleotide sequence data from the open access NCBI FTP BLAST database^36^ (ftp://ftp.ncbi.nlm.nih.gov/blast/db/FASTA/env_nt.gz) were downloaded on November 3, 2017; CRISPR arrays and spacers were identified using MetaCRT with default parameters; and contigs with a CRISPR region were selected. Putative proteins were predicted using Prodigal with metagenome parameters; those within 15 kb upstream and downstream of the CRISPR region were selected, and their predicted protein sequences were saved and uploaded into a local BLASTp database. Amino acid sequences of 16 Cas12a were downloaded from the UniProt database on the same day. Conserved motifs across Cas12a proteins were identified based on protein alignments of structurally identified Cas12a sequences using MAFFT (version 7, https://mafft.cbrc.jp/alignment/server/)^37^ with default parameters and saved for later use as query. A local BLASTp was run with the conserved Cas12a motifs defined above as query, and putative Cas12a proteins with a size between 800 and 1500 amino acids were retained. An online BLASTp was run against non-redundant protein sequences with all candidates for preliminary annotation. Sequences not starting with methionine were excluded. All new putative Cas12a proteins were aligned using MAFFT version 7 online using default parameters, from which a phylogenetic tree was drawn using the neighbor-joining method with pairwise deletion and 100 × bootstrap in MEGA7. Based on the results of the preliminary annotation, candidates annotated as putative Cas12a proteins and whose encoding genes were adjacent to CRISPR arrays were selected. Gene fragments due to low-quality alignments were discarded after structure-based comparison between sequences using ESPript 3.0^38^. A phylogenetic tree of all new Cas12a proteins and 16 previously characterized Cas12a proteins was constructed using FastTree 2.1, 1000 × bootstrap, with default parameters and visualized using iTOL v6 after an alignment with MAFFT online.

### Plasmid construction for protein purification

The coding sequences of *mgCas12a-1*, *mgCas12a-2* (synthesized by Bionics, Seoul, South Korea), *AsCas12a* (Addgene #102565), *FnCas12a* (Addgene #102565), and *LbCas12a* (Addgene #102566) were cloned in-frame between the sequence encoding a polyhistidine tag and a bipartite nuclear localization signal (BPNLS) in a modified pET28a vector. The catalytic residues of each mgCas12a protein were determined by multiple amino acid sequence alignment of AsCas12a, FnCas12a, and LbCas12a. Catalytically inactive mutants were generated by PCR mutagenesis using Gibson Assembly Master Mix (New England Biolabs, Ipswich, MA, USA).

### Protein production and purification

The coding sequences of *Cas12a* were individually cloned into the pET28a vector. An 1-mL aliquot of an overnight-grown culture of Rosetta2 (DE3) pLysS cells containing individual pET28a-*Cas12a* constructs (Supplementary Fig. 12a) was inoculated into 1 L LB growth medium with 50 mg/L kanamycin. The cultures were incubated on a shaking incubator (150 RPM, 37 °C) until OD_600_ reached 0.5–0.6. The temperature and the shaking speed were then lowered to 18 °C and 120 RPM, respectively; isopropyl β-D-1-thiogalactopyranoside (1 mM final concentration) was added to each culture; and cultures were incubated for an additional 20 h at 18 °C to allow protein production. Each culture was harvested by centrifugation (3,700 g, 4 °C, 1 h), and the resulting cell paste was resuspended in 50 mL lysis buffer (20 mM Tris–HCl, pH 8.0, 500 mM NaCl, 5 mM imidazole, 1 mM DTT, and 1 mM PMSF), followed by lysis via sonication (2 s on, 10 s off, 40% amplification, 15-min duration). Each supernatant was then separated from the cell lysate by centrifugation (3700×*g*, 4 °C, 1 h) and filtered through 0.5-µm and 0.45-μm syringe filters (polyethersulfone syringe filter, Satorius, Göttingen, Germany) to remove any remaining cell debris and impurities. The filtered supernatant was then applied to an equilibrated fast protein liquid chromatography (FPLC) system (Äkta, GE Healthcare, Chicago, IL, USA) and flowed through a nickel column (HisTrap HP 5 mL, GE Healthcare). Nickel column–bound proteins were then eluted with elution buffer (20 mM Tris–HCl, pH 8.0, 500 mM NaCl, and 500 mM imidazole) in a fill-in step gradient manner from 14 to 100%. Fractions were separated by SDS-PAGE to identify those with Cas12a protein; positive fractions were combined for concentration using a protein concentrator (Vivaspin Turbo 15 50,000 MWCO; Sartorius). The concentrated proteins were injected into a desalting column (HiPrep 26/10 Desalting; GE Healthcare) equilibrated with desalting buffer (20 mM HEPES–KOH, pH 7.5, 300 mM KCl, 1 mM DTT, and 10% [v/v] glycerol) to ensure the long-term stability of the eluted proteins. The purity of each eluate was analyzed by SDS-PAGE (Supplementary Fig. 12d). Finally, the proteins were concentrated on a Vivaspin Turbo 15 concentrator. Protein concentration was determined with a conventional Bradford assay, and the protein samples were stored at − 80 °C with the addition of 1 mM 1,4-dithiothreitol until further use.

### In-tube DNA cleavage assays and quantification of enzymatic activity

Target DNA #1, #2 and #3 (*HsEMX1*, *HsDNMT1*, and *HsCCR5*, respectively) were initially amplified from genomic DNA extracted from HEK293T cells and individually cloned into a pUC-based cloning vector (Biofact, Daejeon, South Korea) to provide a template for PCR amplification. The target amplicons were purified (PCR purification kit, Qiagen, Westberg, Germany) before in-tube DNA cleavage assays. All crRNAs used in this study were synthesized by IDT (Coralville, IA, USA). Sequences of target DNAs, crRNAs and oligonucleotides are listed in Supplementary Tables 5, 6 and 7, respectively. The Cas12a RNP complex was formed by incubating 4 pmol Cas12a protein and 4.8 pmol crRNA in deionized water with 1 × reaction buffer (NEBuffer 1.1, NEB) at room temperature for 10 min. Then, 0.5 pmol target DNA was added to the tube containing the Cas12a RNP complex to begin in-tube target DNA cleavage in a 37 °C water bath. Reactions were stopped by adding 1 µL of proteinase K (NEB, #P8107S) (30-min incubation at 37 °C), followed by DNA loading buffer containing SDS and EDTA (6 × purple gel loading dye, NEB). Each reaction was then loaded onto a 1% (w/v) agarose gel for DNA electrophoresis before quantification of digested and undigested target DNA bands with ImageJ^39^ to determine the cleavage efficiency of each Cas12a RNP complex. Prism GraphPad was used for statistical analysis and data plotting.

### Nucleofection of mgCas12a-1 RNPs for HsCCR5 knock-out

The purified mgCas12a-1 (126 pmol) and synthetic full-length crRNA1 or crRNA3 (44-mer, 150 pmol; IDT) were mixed and incubated at room temperature for 20 min for RNP assembly. After adding 1 µL Alt-R® Cas12a (Cpf1) Electroporation Enhancer (78 µM, IDT), the RNP complexes were individually transfected into the HEK293T cells (3.5 × 10^5^ cells) using 4D-Nucleofector (Lonza, Basel, Switzerland) with the CM-130 program.

### T7 endonuclease I (T7E1) assay

After harvesting transfected HEK293T cells at 24 h, genomic DNA was isolated using a DNeasy Blood & Tissue Kit (Qiagen) following the manufacturer’s instructions. Using the genomic DNA as a template, PCR amplification was performed with HsCCR5 gene-specific primers to amplify the substrate DNA for T7E1 assay. Purified PCR products (300 ng) in NEBuffer 2 (NEB) were denatured (95 °C for 10 min), and re-annealed by gradually cooling from 95 to 85 °C at − 2 °C/s and 85 to 25 °C at − 0.1 °C/s and then held at 4 °C using a thermocycler, and then the PCR products were treated with 10 U of T7 endonuclease 1 (T7E1; NEB) in a 20 µL final reaction at 37 °C for 15 min. The products were analyzed on a 2.0% agarose gel. The DNA band intensity was determined using ImageJ software.

## Supplementary Information

Supplementary Figures.

Supplementary Tables.

## Data Availability

The datasets used and/or analyzed during the current study available from the corresponding author on reasonable request.

## References

[1] Swarts DC, van der Oost J, Jinek M (2017). Structural basis for guide RNA processing and seed-dependent DNA targeting by CRISPR-Cas12a. Mol. Cell.

[2] Makarova KS (2015). An updated evolutionary classification of CRISPR-Cas systems. Nat. Rev. Microbiol..

[3] Hille F (2018). The biology of CRISPR-Cas: Backward and forward. Cell.

[4] Fonfara I, Richter H, BratoviÄ M, Le Rhun A, Charpentier E (2016). The CRISPR-associated DNA-cleaving enzyme Cpf1 also processes precursor CRISPR RNA. Nature.

[5] Makarova KS (2020). Evolutionary classification of CRISPR–Cas systems: A burst of class 2 and derived variants. Nat. Rev. Microbiol..

[6] Deltcheva E (2011). CRISPR RNA maturation by trans-encoded small RNA and host factor RNase III. Nature.

[7] Jinek M (2012). A Programmable dual-RNA-guided DNA endonuclease in adaptive bacterial immunity. Science.

[8] Jiang F, Zhou K, Ma L, Gressel S, Doudna JA (2015). A Cas9-guide RNA complex preorganized for target DNA recognition. Science.

[9] Zetsche B (2015). Cpf1 is a single RNA-guided endonuclease of a class 2 CRISPR-cas system. Cell.

[10] Yamano T (2016). Crystal structure of Cpf1 in complex with guide RNA and target DNA. Cell.

[11] Gier RA (2020). High-performance CRISPR-Cas12a genome editing for combinatorial genetic screening. Nat. Commun..

[12] Tóth E (2020). Improved LbCas12a variants with altered PAM specificities further broaden the genome targeting range of Cas12a nucleases. Nucleic Acids Res..

[13] Kleinstiver BP (2019). Engineered CRISPR–Cas12a variants with increased activities and improved targeting ranges for gene, epigenetic and base editing. Nat. Biotechnol..

[14] Slaymaker IM (2016). Rationally engineered Cas9 nucleases with improved specificity. Science.

[15] Chen JS (2017). Enhanced proofreading governs CRISPR-Cas9 targeting accuracy. Nature.

[16] Kleinstiver BP (2016). High-fidelity CRISPR-Cas9 nucleases with no detectable genome-wide off-target effects. Nature.

[17] Gaudelli NM (2017). Programmable base editing of A·T to G·C in genomic DNA without DNA cleavage. Nature.

[18] Li X (2018). Base editing with a Cpf1-cytidine deaminase fusion. Nat. Biotechnol..

[19] Anzalone AV (2019). Search-and-replace genome editing without double-strand breaks or donor DNA. Nature.

[20] Xu X, Qi LS (2019). A CRISPR–dCas toolbox for genetic engineering and synthetic biology. J. Mol. Biol..

[21] Kim SK (2017). Efficient transcriptional gene repression by type V-A CRISPR-Cpf1 from *Eubacterium eligens*. ACS Synth. Biol..

[22] Zhang X (2017). Multiplex gene regulation by CRISPR-ddCpf1. Cell Discov..

[23] Zhong G, Wang H, Li Y, Tran MH, Farzan M (2017). Cpf1 proteins excise CRISPR RNAs from mRNA transcripts in mammalian cells. Nat. Chem. Biol..

[24] Chen JS (2018). CRISPR-Cas12a target binding unleashes indiscriminate single-stranded DNase activity. Science.

[25] Gootenberg JS (2018). Multiplexed and portable nucleic acid detection platform with Cas13, Cas12a, and Csm6. Science.

[26] Broughton JP (2020). CRISPR–Cas12-based detection of SARS-CoV-2. Nat. Biotechnol..

[27] Li B (2020). CRISPR-Cas12a possesses unconventional DNase activity that can be inactivated by synthetic oligonucleotides. Mol. Ther. Nucleic Acids.

[28] Raymond F (2016). The initial state of the human gut microbiome determines its reshaping by antibiotics. ISME J..

[29] Ahn WC (2019). In vivo genome editing using the Cpf1 ortholog derived from *Eubacterium eligens*. Sci. Rep..

[30] Stella S, Alcón P, Montoya G (2017). Structure of the Cpf1 endonuclease R-loop complex after target DNA cleavage. Nature.

[31] Schunder E, Rydzewski K, Grunow R, Heuner K (2013). First indication for a functional CRISPR/Cas system in *Francisella tularensis*. Int. J. Med. Microbiol..

[32] Vestergaard G, Garrett RA, Shah SA (2014). CRISPR adaptive immune systems of Archaea. RNA Biol..

[33] Sundaresan R, Parameshwaran HP, Yogesha SD, Keilbarth MW, Rajan R (2017). RNA-independent DNA cleavage activities of Cas9 and Cas12a. Cell Rep..

[34] Mohanraju P, Oost J, Jinek M, Swarts D (2018). Heterologous expression and purification of the CRISPR-Cas12a/Cpf1 protein. Bio-Protoc..

[35] Reyon D (2012). FLASH assembly of TALENs for high-throughput genome editing. Nat. Biotechnol..

[36] Altschul SF, Gish W, Miller W, Myers EW, Lipman DJ (1990). Basic local alignment search tool. J. Mol. Biol..

[37] Katoh K, Rozewicki J, Yamada KD (2018). MAFFT online service: Multiple sequence alignment, interactive sequence choice and visualization. Brief. Bioinform..

[38] Robert X, Gouet P (2014). Deciphering key features in protein structures with the new ENDscript server. Nucleic Acids Res..

[39] Schneider CA, Rasband WS, Eliceiri KW (2012). NIH Image to ImageJ: 25 years of image analysis. Nat. Methods.

