# Supplementary material for: Biochemical characterization of the two novel mgCas12a proteins from the human gut metagenome
Source: Sci Rep. 2022 Dec 2;12:20857. doi: 10.1038/s41598-022-25227-w (PMC9718762; doi:10.1038/s41598-022-25227-w)
Supplement: Supplementary file 1 — Supplementary Figures. [file 41598_2022_25227_MOESM1_ESM.pptx]

## Slide 1
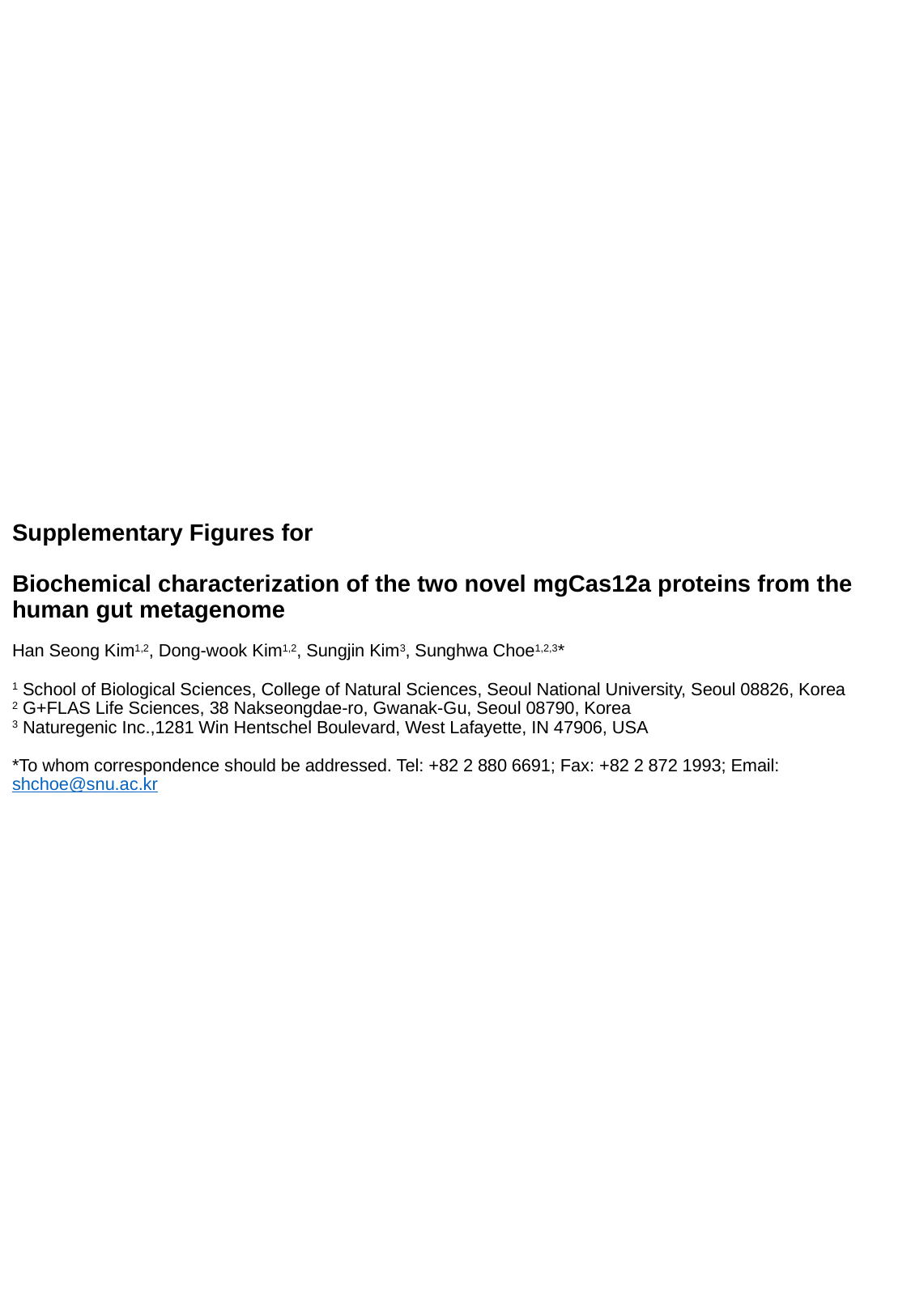

# Supplementary Figures for Biochemical characterization of the two novel mgCas12a proteins from the human gut metagenome Han Seong Kim1,2, Dong-wook Kim1,2, Sungjin Kim3, Sunghwa Choe1,2,3* 1 School of Biological Sciences, College of Natural Sciences, Seoul National University, Seoul 08826, Korea 2 G+FLAS Life Sciences, 38 Nakseongdae-ro, Gwanak-Gu, Seoul 08790, Korea 3 Naturegenic Inc.,1281 Win Hentschel Boulevard, West Lafayette, IN 47906, USA *To whom correspondence should be addressed. Tel: +82 2 880 6691; Fax: +82 2 872 1993; Email: shchoe@snu.ac.kr

## Slide 2
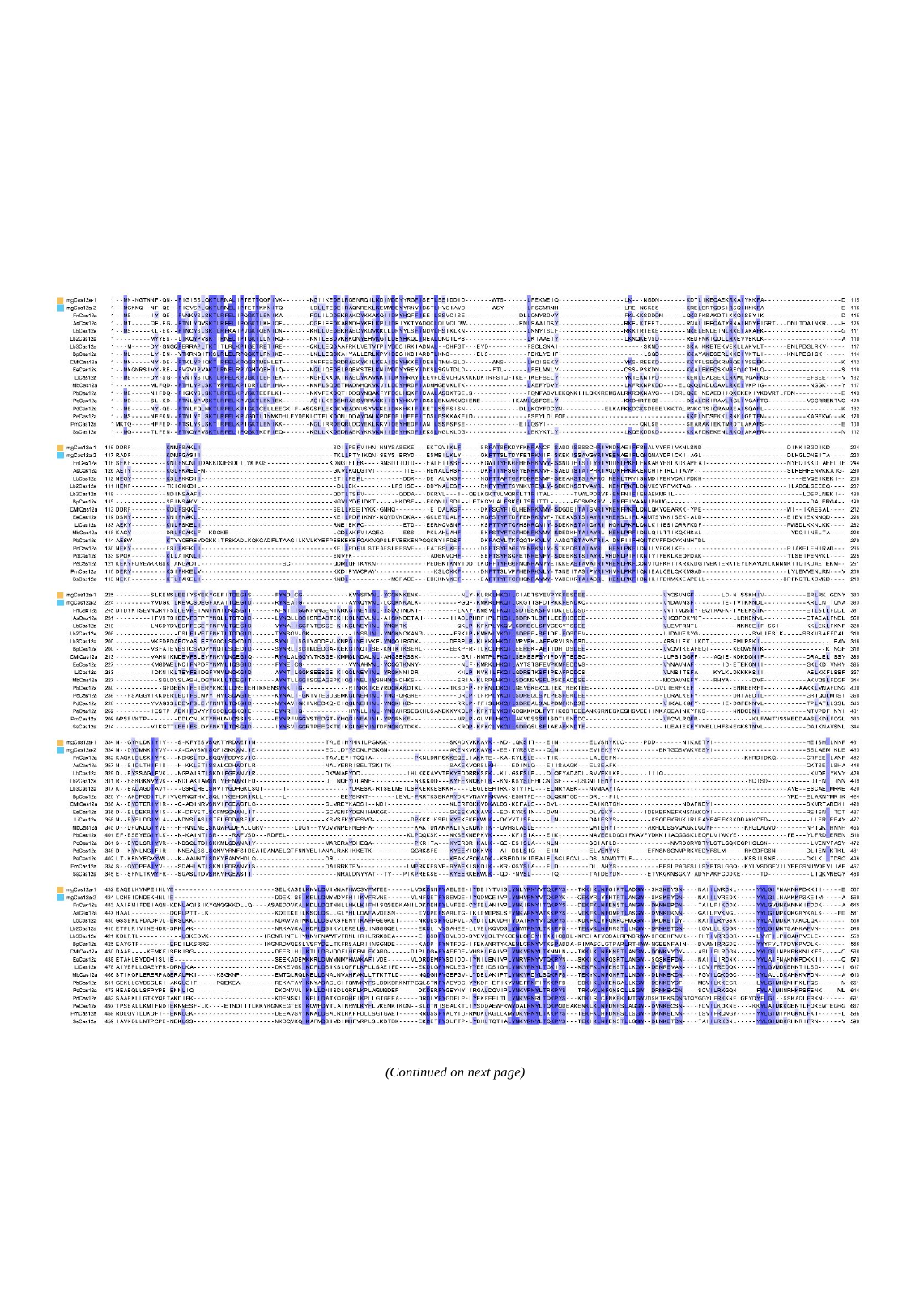

(Continued on next page)

## Slide 3
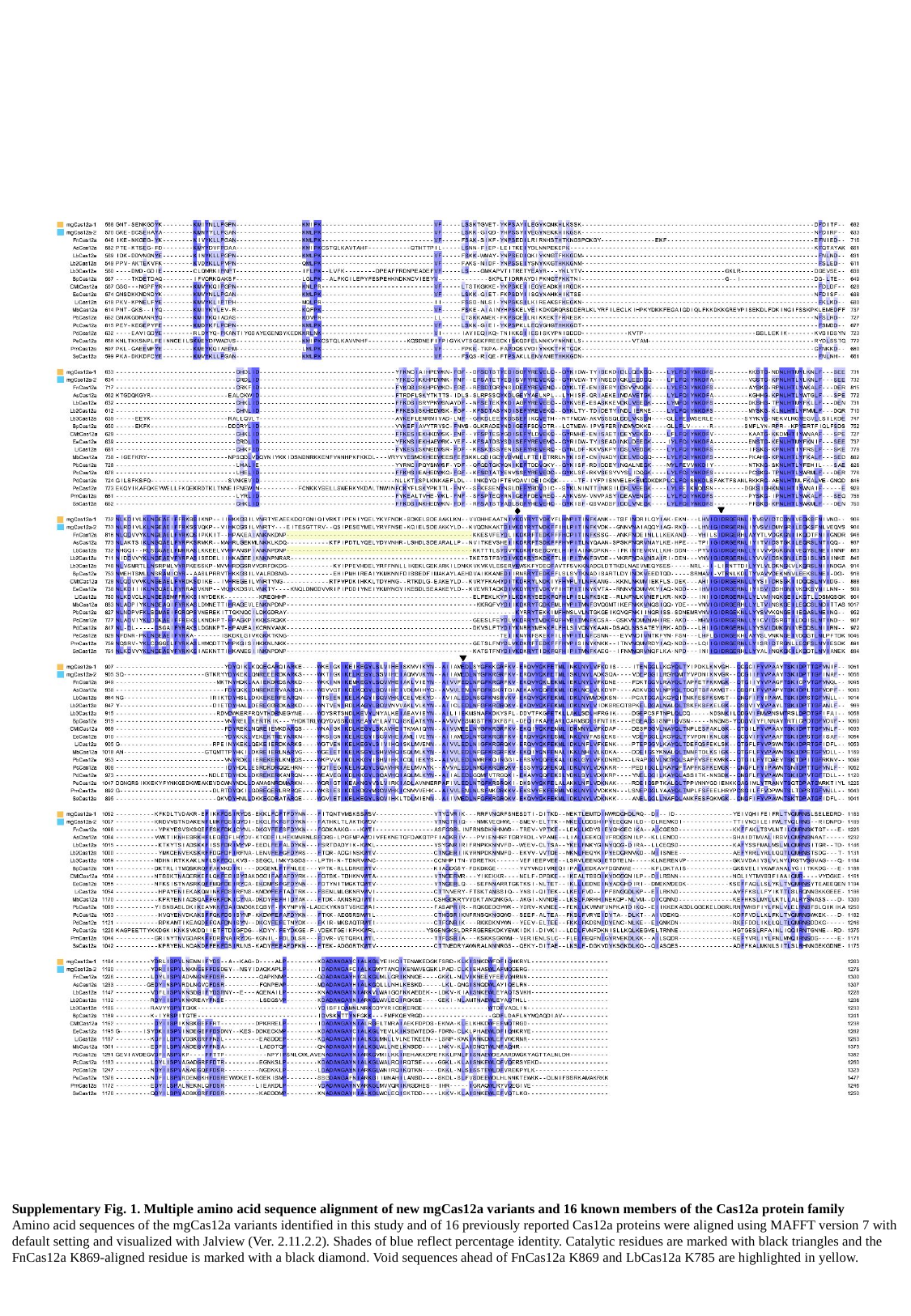

Supplementary Fig. 1. Multiple amino acid sequence alignment of new mgCas12a variants and 16 known members of the Cas12a protein family
Amino acid sequences of the mgCas12a variants identified in this study and of 16 previously reported Cas12a proteins were aligned using MAFFT version 7 with default setting and visualized with Jalview (Ver. 2.11.2.2). Shades of blue reflect percentage identity. Catalytic residues are marked with black triangles and the FnCas12a K869-aligned residue is marked with a black diamond. Void sequences ahead of FnCas12a K869 and LbCas12a K785 are highlighted in yellow.

## Slide 4
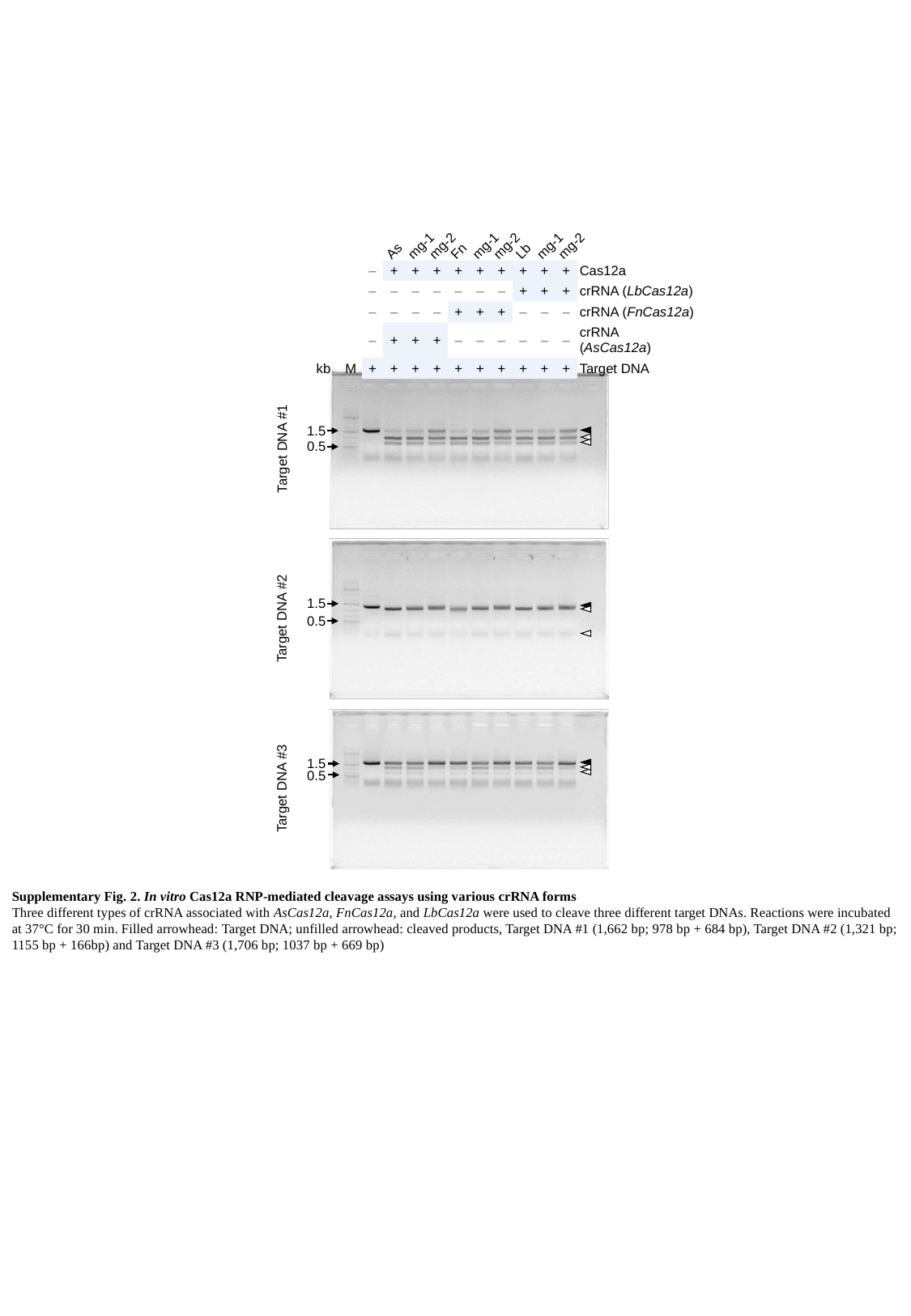

As
mg-1
mg-2
Fn
mg-1
mg-2
Lb
mg-1
mg-2
| | | – | + | + | + | + | + | + | + | + | + | Cas12a |
| --- | --- | --- | --- | --- | --- | --- | --- | --- | --- | --- | --- | --- |
| | | – | – | – | – | – | – | – | + | + | + | crRNA (LbCas12a) |
| | | – | – | – | – | + | + | + | – | – | – | crRNA (FnCas12a) |
| | | – | + | + | + | – | – | – | – | – | – | crRNA (AsCas12a) |
| kb | M | + | + | + | + | + | + | + | + | + | + | Target DNA |
1.5
0.5
Target DNA #1
1.5
Target DNA #2
0.5
1.5
0.5
Target DNA #3
Supplementary Fig. 2. In vitro Cas12a RNP-mediated cleavage assays using various crRNA forms
Three different types of crRNA associated with AsCas12a, FnCas12a, and LbCas12a were used to cleave three different target DNAs. Reactions were incubated at 37°C for 30 min. Filled arrowhead: Target DNA; unfilled arrowhead: cleaved products, Target DNA #1 (1,662 bp; 978 bp + 684 bp), Target DNA #2 (1,321 bp; 1155 bp + 166bp) and Target DNA #3 (1,706 bp; 1037 bp + 669 bp)

## Slide 5
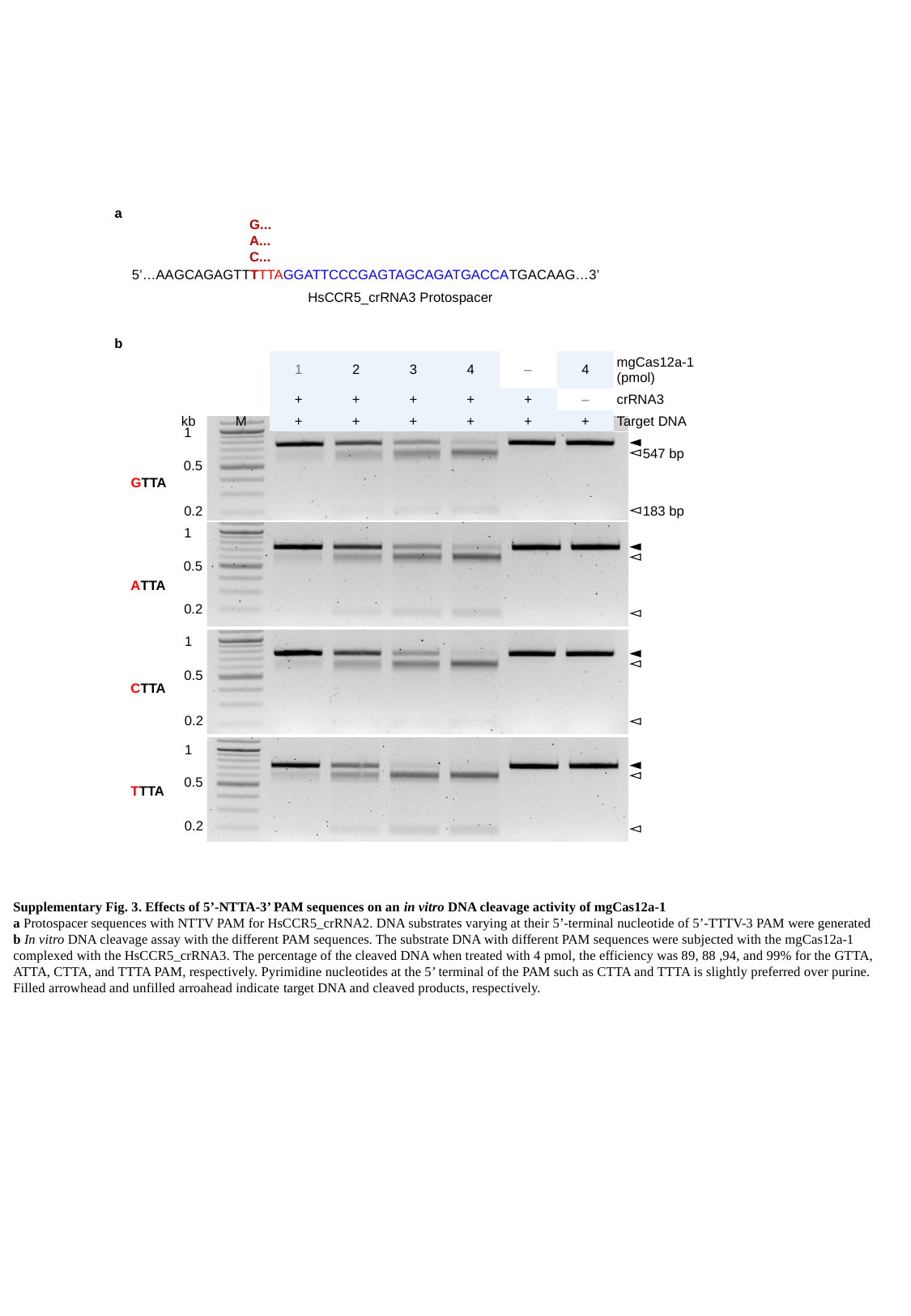

a
G...
A...
C...
5’…AAGCAGAGTTTTTAGGATTCCCGAGTAGCAGATGACCATGACAAG…3’
HsCCR5_crRNA3 Protospacer
b
| | | 1 | 2 | 3 | 4 | – | 4 | mgCas12a-1 (pmol) |
| --- | --- | --- | --- | --- | --- | --- | --- | --- |
| | | + | + | + | + | + | – | crRNA3 |
| kb | M | + | + | + | + | + | + | Target DNA |
1
547 bp
0.5
GTTA
183 bp
0.2
1
0.5
ATTA
0.2
1
0.5
CTTA
0.2
1
0.5
TTTA
0.2
Supplementary Fig. 3. Effects of 5’-NTTA-3’ PAM sequences on an in vitro DNA cleavage activity of mgCas12a-1
a Protospacer sequences with NTTV PAM for HsCCR5_crRNA2. DNA substrates varying at their 5’-terminal nucleotide of 5’-TTTV-3 PAM were generated
b In vitro DNA cleavage assay with the different PAM sequences. The substrate DNA with different PAM sequences were subjected with the mgCas12a-1 complexed with the HsCCR5_crRNA3. The percentage of the cleaved DNA when treated with 4 pmol, the efficiency was 89, 88 ,94, and 99% for the GTTA, ATTA, CTTA, and TTTA PAM, respectively. Pyrimidine nucleotides at the 5’ terminal of the PAM such as CTTA and TTTA is slightly preferred over purine. Filled arrowhead and unfilled arroahead indicate target DNA and cleaved products, respectively.

## Slide 6
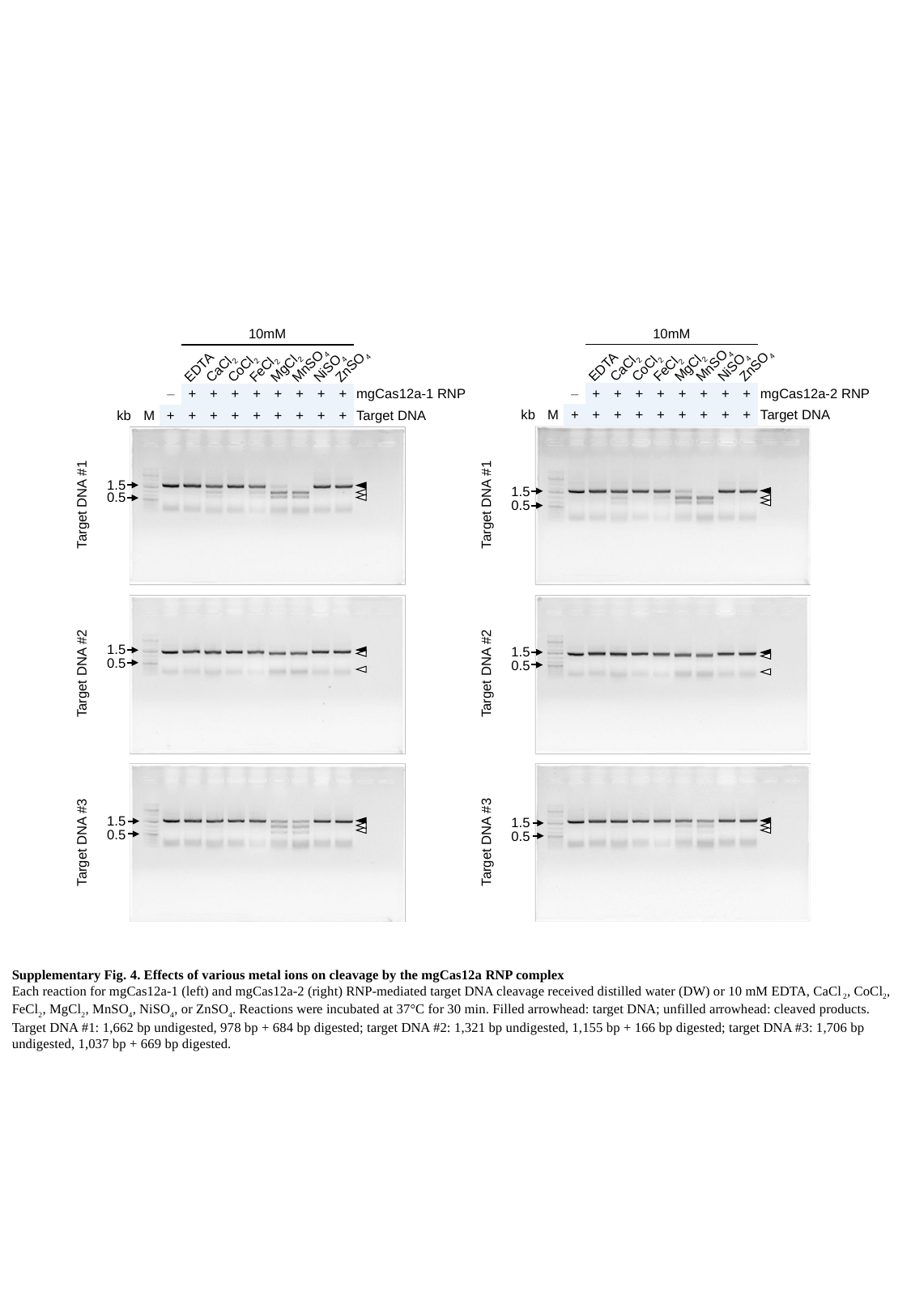

| | | | 10mM | | | | | | | | |
| --- | --- | --- | --- | --- | --- | --- | --- | --- | --- | --- | --- |
| | | | | | | | | | | | |
| | | – | + | + | + | + | + | + | + | + | mgCas12a-2 RNP |
| kb | M | + | + | + | + | + | + | + | + | + | Target DNA |
| | | | 10mM | | | | | | | | |
| --- | --- | --- | --- | --- | --- | --- | --- | --- | --- | --- | --- |
| | | | | | | | | | | | |
| | | – | + | + | + | + | + | + | + | + | mgCas12a-1 RNP |
| kb | M | + | + | + | + | + | + | + | + | + | Target DNA |
EDTA
CaCl2
CoCl2
FeCl2
MgCl2
MnSO4
NiSO4
ZnSO4
EDTA
CaCl2
CoCl2
FeCl2
MgCl2
MnSO4
NiSO4
ZnSO4
1.5
1.5
0.5
Target DNA #1
Target DNA #1
0.5
1.5
1.5
0.5
0.5
Target DNA #2
Target DNA #2
1.5
1.5
0.5
0.5
Target DNA #3
Target DNA #3
Supplementary Fig. 4. Effects of various metal ions on cleavage by the mgCas12a RNP complex
Each reaction for mgCas12a-1 (left) and mgCas12a-2 (right) RNP-mediated target DNA cleavage received distilled water (DW) or 10 mM EDTA, CaCl2, CoCl2, FeCl2, MgCl2, MnSO4, NiSO4, or ZnSO4. Reactions were incubated at 37°C for 30 min. Filled arrowhead: target DNA; unfilled arrowhead: cleaved products. Target DNA #1: 1,662 bp undigested, 978 bp + 684 bp digested; target DNA #2: 1,321 bp undigested, 1,155 bp + 166 bp digested; target DNA #3: 1,706 bp undigested, 1,037 bp + 669 bp digested.

## Slide 7
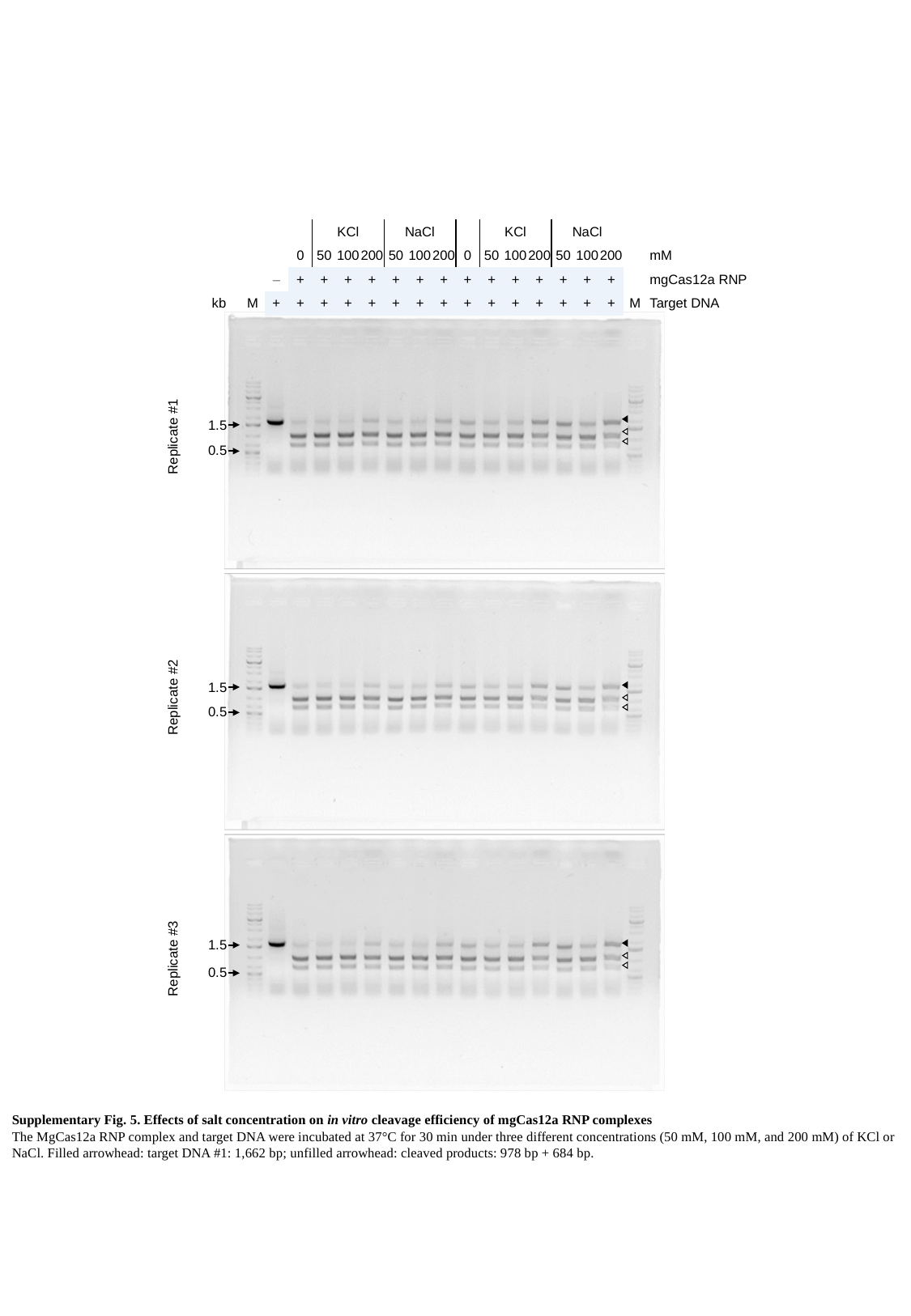

| | | | | KCl | | | NaCl | | | | KCl | | | NaCl | | | | |
| --- | --- | --- | --- | --- | --- | --- | --- | --- | --- | --- | --- | --- | --- | --- | --- | --- | --- | --- |
| | | | 0 | 50 | 100 | 200 | 50 | 100 | 200 | 0 | 50 | 100 | 200 | 50 | 100 | 200 | | mM |
| | | – | + | + | + | + | + | + | + | + | + | + | + | + | + | + | | mgCas12a RNP |
| kb | M | + | + | + | + | + | + | + | + | + | + | + | + | + | + | + | M | Target DNA |
1.5
Replicate #1
0.5
1.5
Replicate #2
0.5
1.5
Replicate #3
0.5
Supplementary Fig. 5. Effects of salt concentration on in vitro cleavage efficiency of mgCas12a RNP complexes
The MgCas12a RNP complex and target DNA were incubated at 37°C for 30 min under three different concentrations (50 mM, 100 mM, and 200 mM) of KCl or NaCl. Filled arrowhead: target DNA #1: 1,662 bp; unfilled arrowhead: cleaved products: 978 bp + 684 bp.

## Slide 8
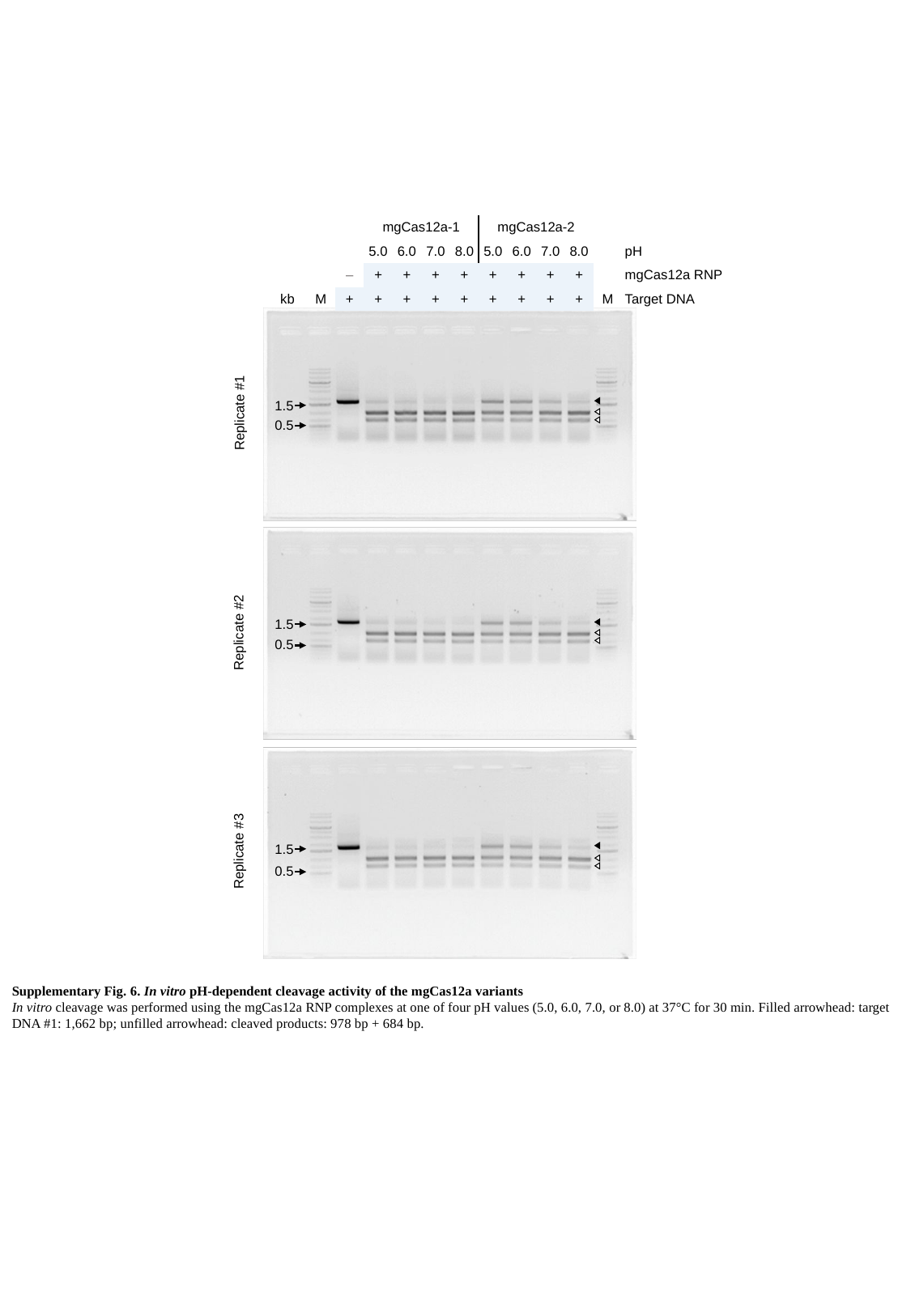

| | | | mgCas12a-1 | | | | mgCas12a-2 | | | | | |
| --- | --- | --- | --- | --- | --- | --- | --- | --- | --- | --- | --- | --- |
| | | | 5.0 | 6.0 | 7.0 | 8.0 | 5.0 | 6.0 | 7.0 | 8.0 | | pH |
| | | – | + | + | + | + | + | + | + | + | | mgCas12a RNP |
| kb | M | + | + | + | + | + | + | + | + | + | M | Target DNA |
1.5
Replicate #1
0.5
1.5
Replicate #2
0.5
1.5
Replicate #3
0.5
Supplementary Fig. 6. In vitro pH-dependent cleavage activity of the mgCas12a variants
In vitro cleavage was performed using the mgCas12a RNP complexes at one of four pH values (5.0, 6.0, 7.0, or 8.0) at 37°C for 30 min. Filled arrowhead: target DNA #1: 1,662 bp; unfilled arrowhead: cleaved products: 978 bp + 684 bp.

## Slide 9
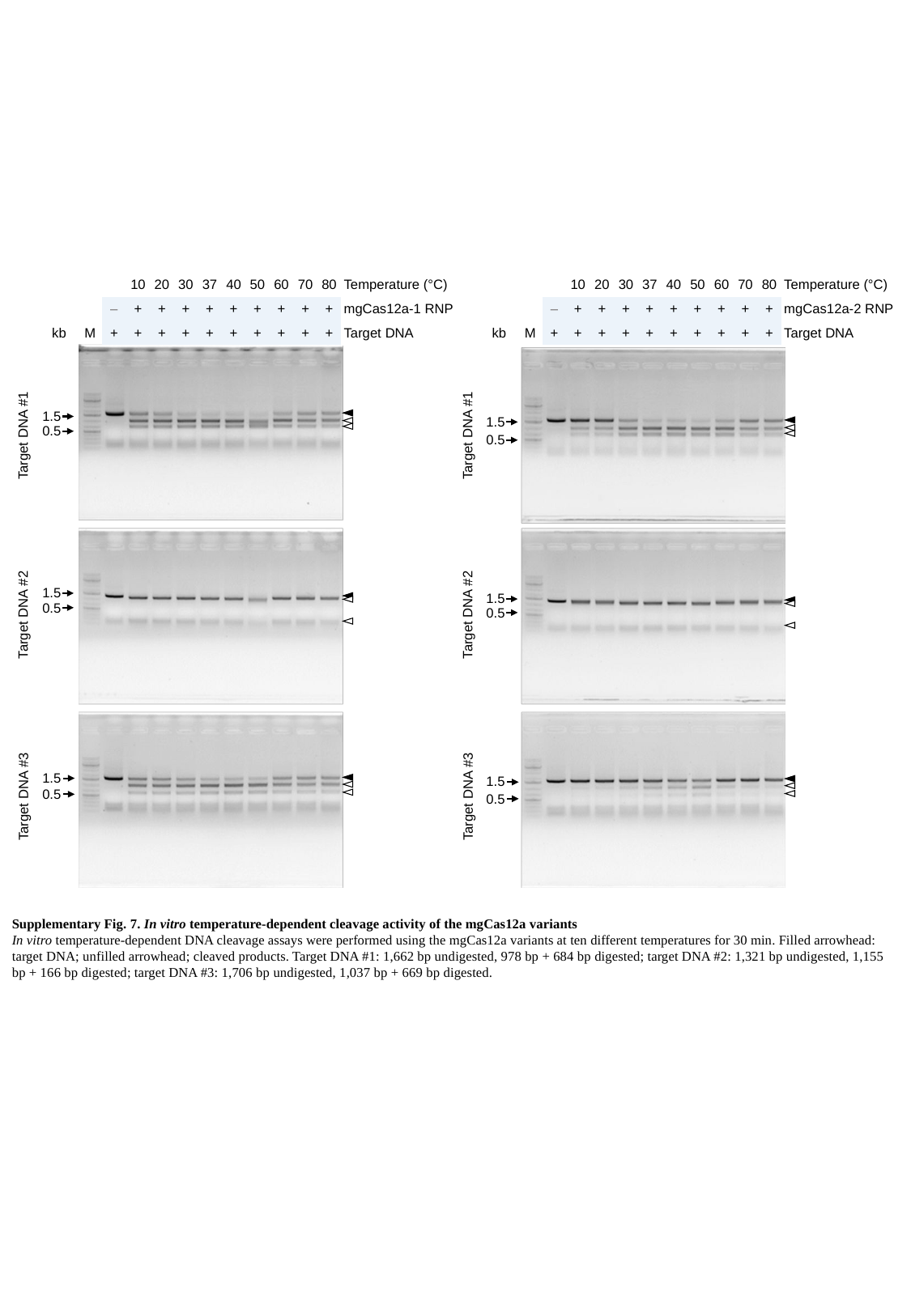

| | | | 10 | 20 | 30 | 37 | 40 | 50 | 60 | 70 | 80 | Temperature (°C) |
| --- | --- | --- | --- | --- | --- | --- | --- | --- | --- | --- | --- | --- |
| | | – | + | + | + | + | + | + | + | + | + | mgCas12a-1 RNP |
| kb | M | + | + | + | + | + | + | + | + | + | + | Target DNA |
| | | | 10 | 20 | 30 | 37 | 40 | 50 | 60 | 70 | 80 | Temperature (°C) |
| --- | --- | --- | --- | --- | --- | --- | --- | --- | --- | --- | --- | --- |
| | | – | + | + | + | + | + | + | + | + | + | mgCas12a-2 RNP |
| kb | M | + | + | + | + | + | + | + | + | + | + | Target DNA |
1.5
1.5
0.5
Target DNA #1
Target DNA #1
0.5
1.5
1.5
0.5
0.5
Target DNA #2
Target DNA #2
1.5
1.5
0.5
Target DNA #3
Target DNA #3
0.5
Supplementary Fig. 7. In vitro temperature-dependent cleavage activity of the mgCas12a variants
In vitro temperature-dependent DNA cleavage assays were performed using the mgCas12a variants at ten different temperatures for 30 min. Filled arrowhead: target DNA; unfilled arrowhead; cleaved products. Target DNA #1: 1,662 bp undigested, 978 bp + 684 bp digested; target DNA #2: 1,321 bp undigested, 1,155 bp + 166 bp digested; target DNA #3: 1,706 bp undigested, 1,037 bp + 669 bp digested.

## Slide 10
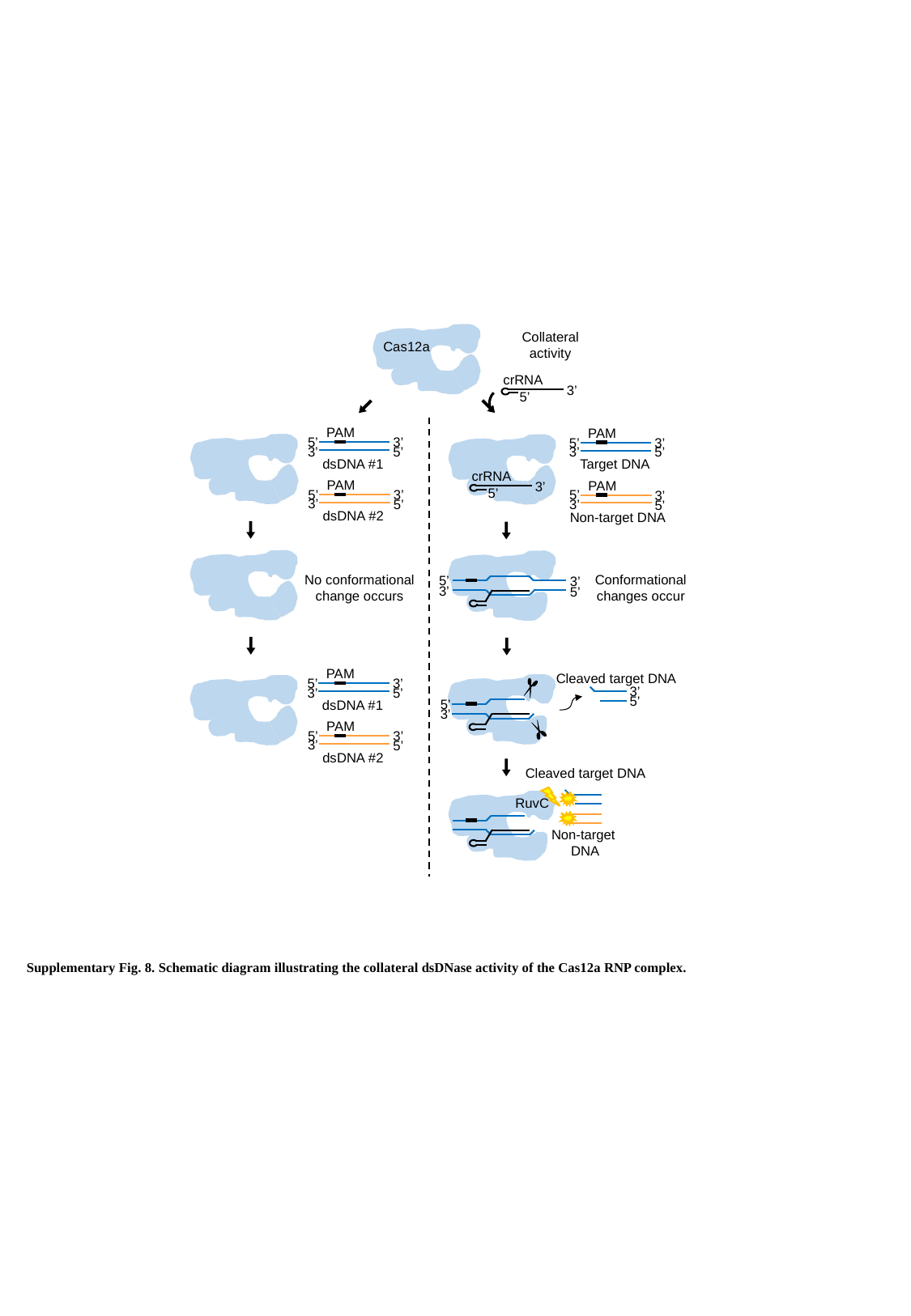

Collateral activity
Cas12a
crRNA
3’
5’
PAM
PAM
5’
3’
5’
3’
3’
5’
3’
5’
dsDNA #1
Target DNA
crRNA
PAM
PAM
3’
5’
5’
3’
5’
3’
3’
5’
3’
5’
dsDNA #2
Non-target DNA
No conformational change occurs
Conformational changes occur
5’
3’
3’
5’
Cleaved target DNA
PAM
5’
3’
3’
3’
5’
5’
dsDNA #1
5’
3’
PAM
5’
3’
3’
5’
dsDNA #2
Cleaved target DNA
RuvC
Non-target
DNA
Supplementary Fig. 8. Schematic diagram illustrating the collateral dsDNase activity of the Cas12a RNP complex.

## Slide 11
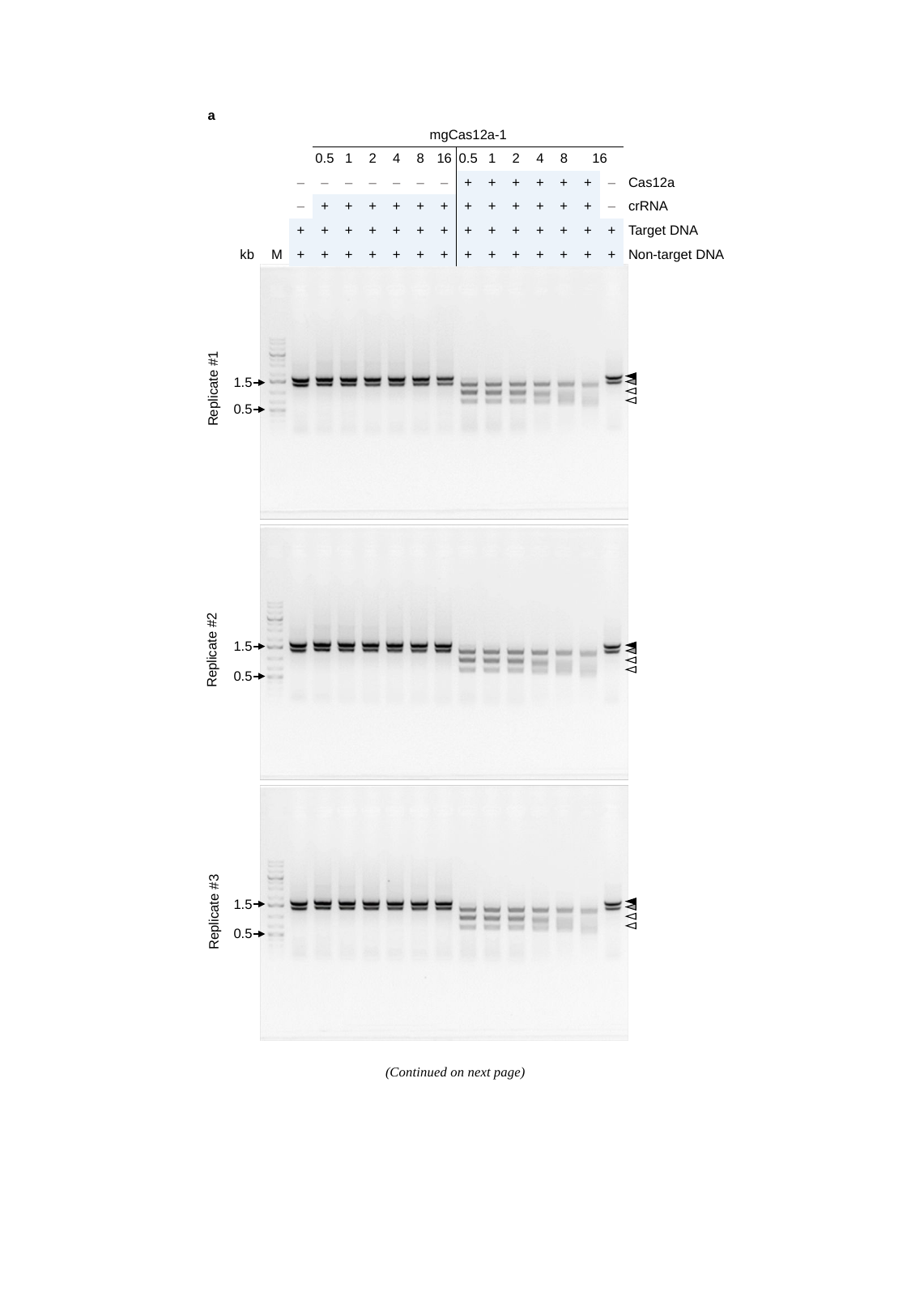

a
| | | | mgCas12a-1 | | | | | | | | | | | | | |
| --- | --- | --- | --- | --- | --- | --- | --- | --- | --- | --- | --- | --- | --- | --- | --- | --- |
| | | | 0.5 | 1 | 2 | 4 | 8 | 16 | 0.5 | 1 | 2 | 4 | 8 | 16 | | |
| | | – | – | – | – | – | – | – | + | + | + | + | + | + | – | Cas12a |
| | | – | + | + | + | + | + | + | + | + | + | + | + | + | – | crRNA |
| | | + | + | + | + | + | + | + | + | + | + | + | + | + | + | Target DNA |
| kb | M | + | + | + | + | + | + | + | + | + | + | + | + | + | + | Non-target DNA |
1.5
Replicate #1
0.5
1.5
Replicate #2
0.5
1.5
Replicate #3
0.5
(Continued on next page)

## Slide 12
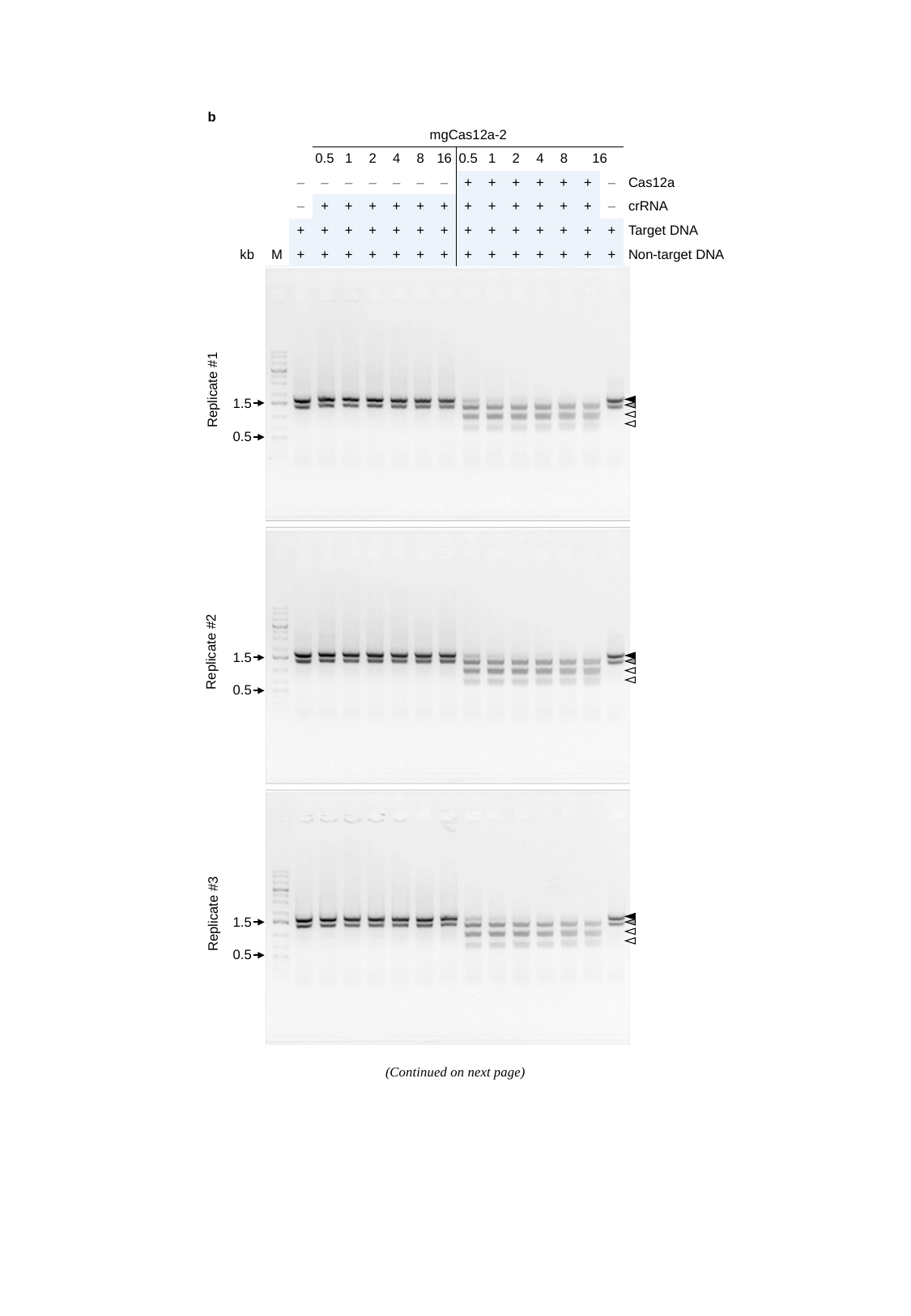

b
| | | | mgCas12a-2 | | | | | | | | | | | | | |
| --- | --- | --- | --- | --- | --- | --- | --- | --- | --- | --- | --- | --- | --- | --- | --- | --- |
| | | | 0.5 | 1 | 2 | 4 | 8 | 16 | 0.5 | 1 | 2 | 4 | 8 | 16 | | |
| | | – | – | – | – | – | – | – | + | + | + | + | + | + | – | Cas12a |
| | | – | + | + | + | + | + | + | + | + | + | + | + | + | – | crRNA |
| | | + | + | + | + | + | + | + | + | + | + | + | + | + | + | Target DNA |
| kb | M | + | + | + | + | + | + | + | + | + | + | + | + | + | + | Non-target DNA |
Replicate #1
1.5
0.5
Replicate #2
1.5
0.5
Replicate #3
1.5
0.5
(Continued on next page)

## Slide 13
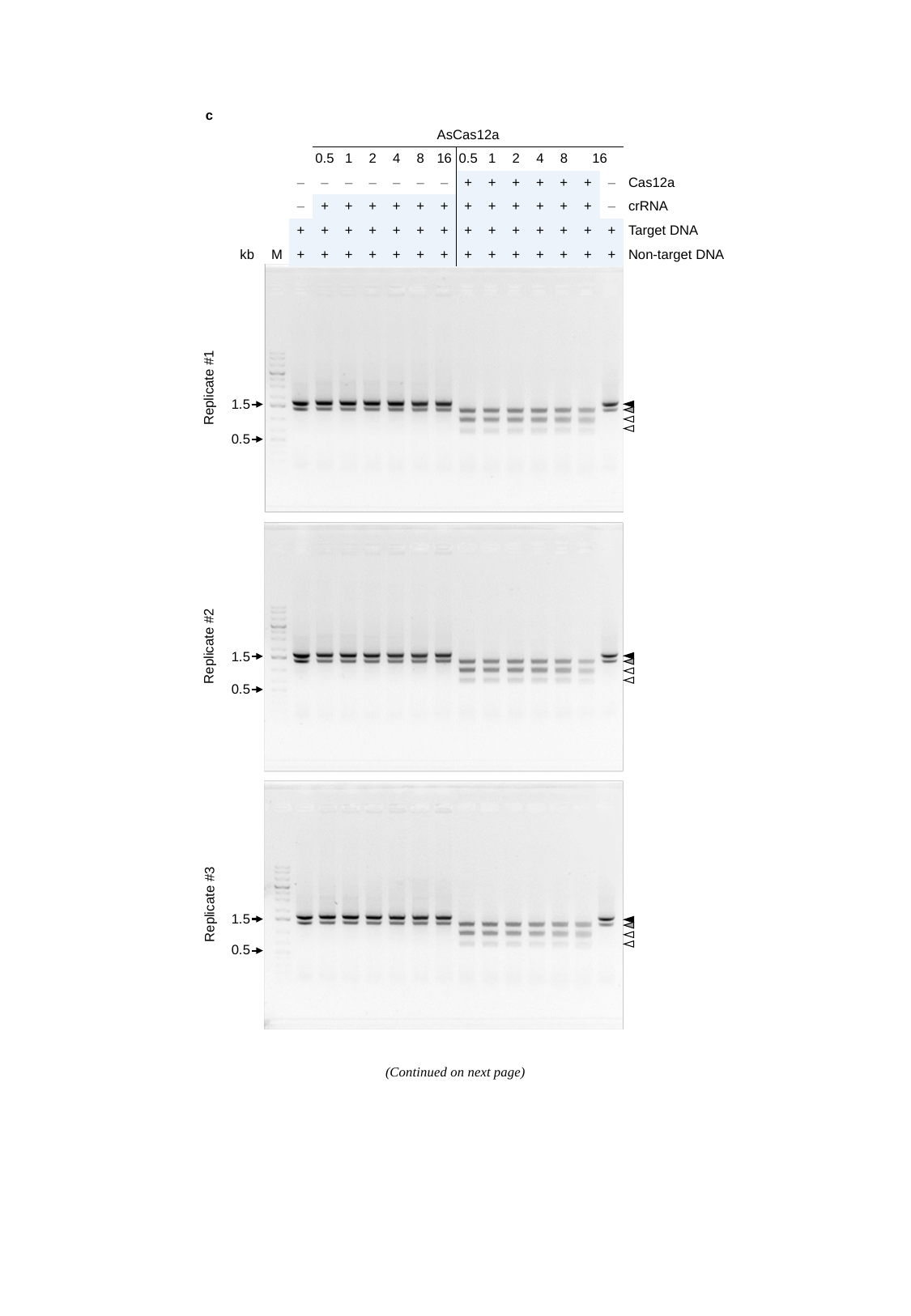

c
| | | | AsCas12a | | | | | | | | | | | | | |
| --- | --- | --- | --- | --- | --- | --- | --- | --- | --- | --- | --- | --- | --- | --- | --- | --- |
| | | | 0.5 | 1 | 2 | 4 | 8 | 16 | 0.5 | 1 | 2 | 4 | 8 | 16 | | |
| | | – | – | – | – | – | – | – | + | + | + | + | + | + | – | Cas12a |
| | | – | + | + | + | + | + | + | + | + | + | + | + | + | – | crRNA |
| | | + | + | + | + | + | + | + | + | + | + | + | + | + | + | Target DNA |
| kb | M | + | + | + | + | + | + | + | + | + | + | + | + | + | + | Non-target DNA |
Replicate #1
1.5
0.5
Replicate #2
1.5
0.5
Replicate #3
1.5
0.5
(Continued on next page)

## Slide 14
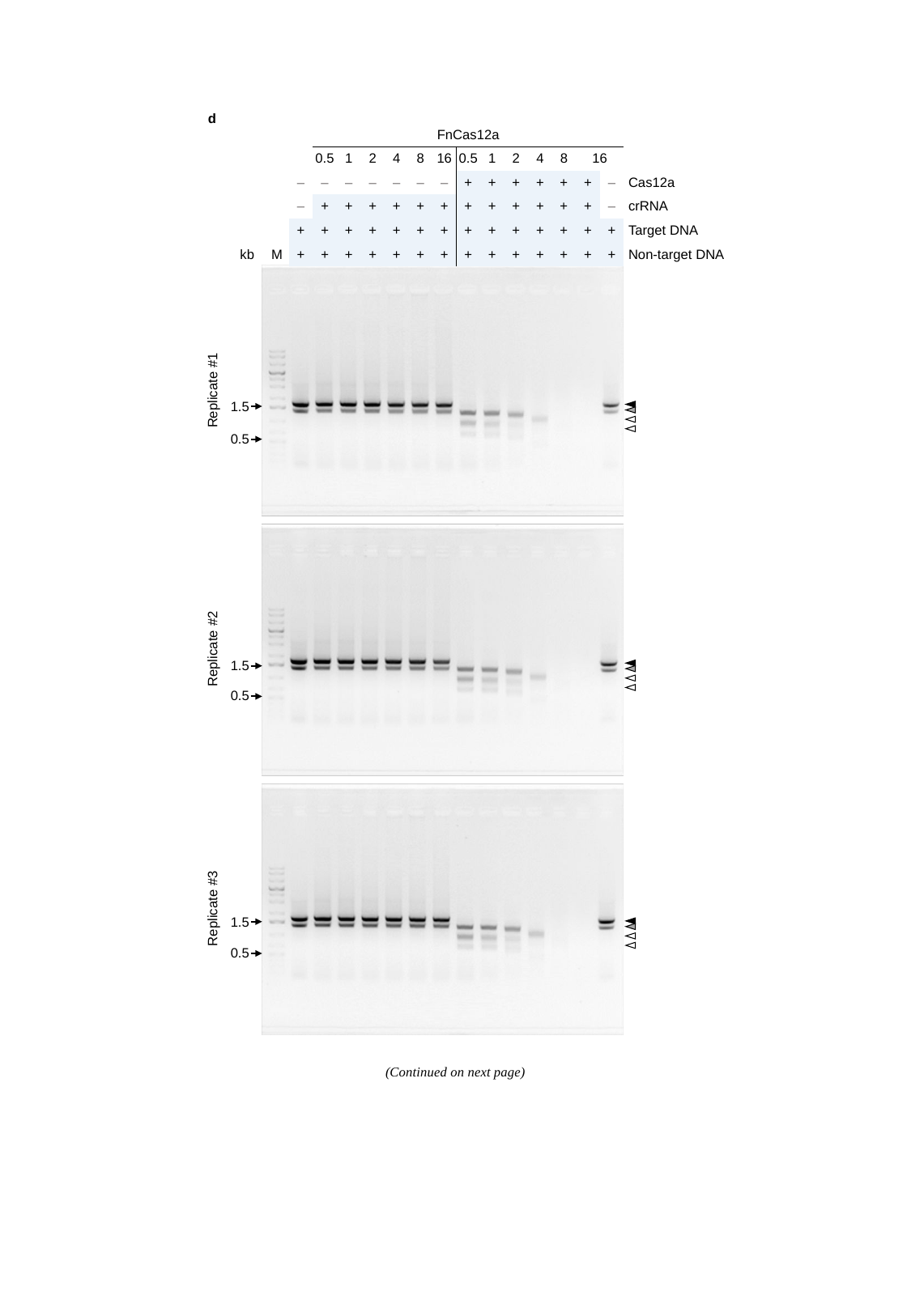

d
| | | | FnCas12a | | | | | | | | | | | | | |
| --- | --- | --- | --- | --- | --- | --- | --- | --- | --- | --- | --- | --- | --- | --- | --- | --- |
| | | | 0.5 | 1 | 2 | 4 | 8 | 16 | 0.5 | 1 | 2 | 4 | 8 | 16 | | |
| | | – | – | – | – | – | – | – | + | + | + | + | + | + | – | Cas12a |
| | | – | + | + | + | + | + | + | + | + | + | + | + | + | – | crRNA |
| | | + | + | + | + | + | + | + | + | + | + | + | + | + | + | Target DNA |
| kb | M | + | + | + | + | + | + | + | + | + | + | + | + | + | + | Non-target DNA |
Replicate #1
1.5
0.5
Replicate #2
1.5
0.5
Replicate #3
1.5
0.5
(Continued on next page)

## Slide 15
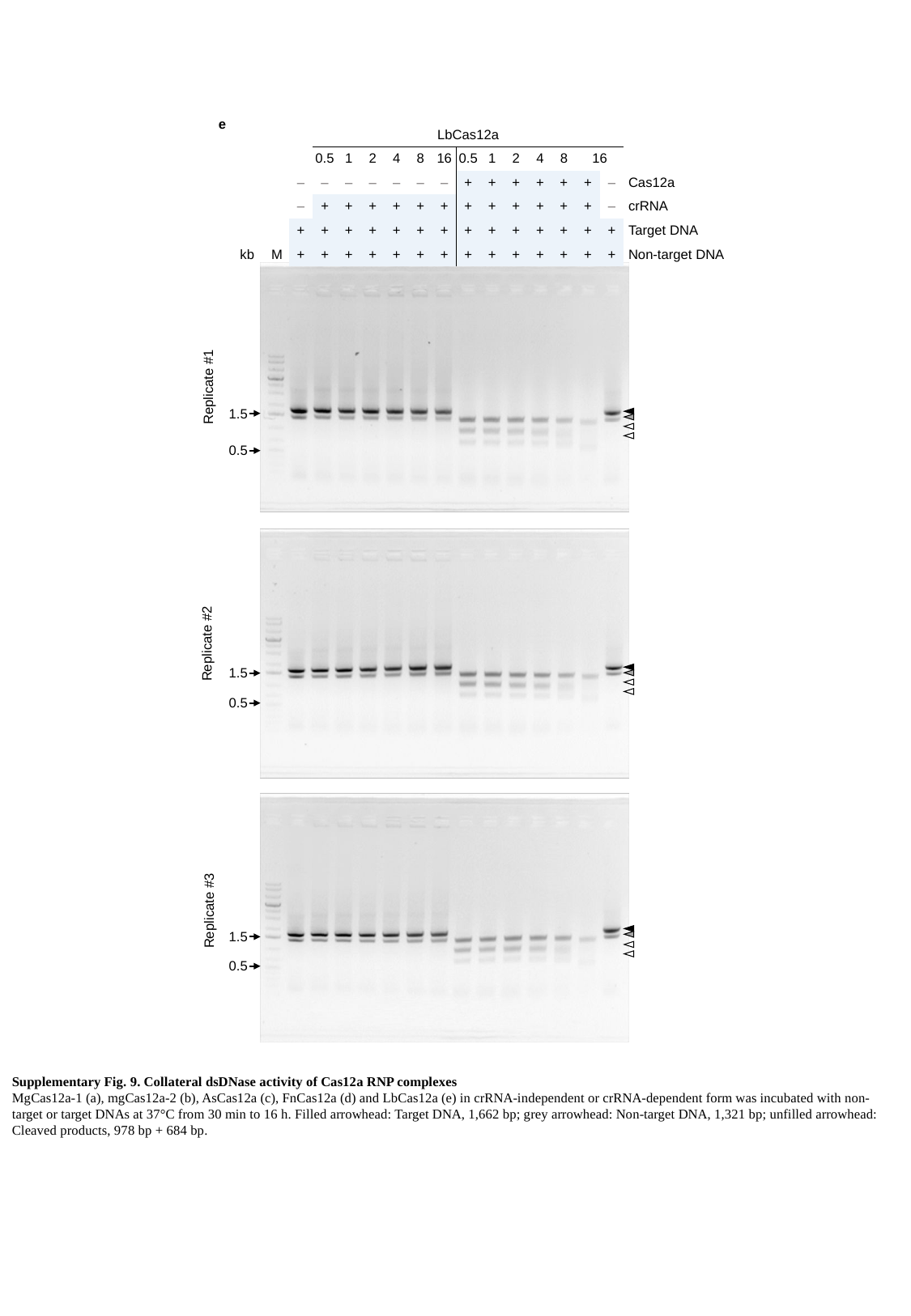

e
| | | | LbCas12a | | | | | | | | | | | | | |
| --- | --- | --- | --- | --- | --- | --- | --- | --- | --- | --- | --- | --- | --- | --- | --- | --- |
| | | | 0.5 | 1 | 2 | 4 | 8 | 16 | 0.5 | 1 | 2 | 4 | 8 | 16 | | |
| | | – | – | – | – | – | – | – | + | + | + | + | + | + | – | Cas12a |
| | | – | + | + | + | + | + | + | + | + | + | + | + | + | – | crRNA |
| | | + | + | + | + | + | + | + | + | + | + | + | + | + | + | Target DNA |
| kb | M | + | + | + | + | + | + | + | + | + | + | + | + | + | + | Non-target DNA |
Replicate #1
1.5
0.5
Replicate #2
1.5
0.5
Replicate #3
1.5
0.5
Supplementary Fig. 9. Collateral dsDNase activity of Cas12a RNP complexes
MgCas12a-1 (a), mgCas12a-2 (b), AsCas12a (c), FnCas12a (d) and LbCas12a (e) in crRNA-independent or crRNA-dependent form was incubated with non-target or target DNAs at 37°C from 30 min to 16 h. Filled arrowhead: Target DNA, 1,662 bp; grey arrowhead: Non-target DNA, 1,321 bp; unfilled arrowhead: Cleaved products, 978 bp + 684 bp.

## Slide 16
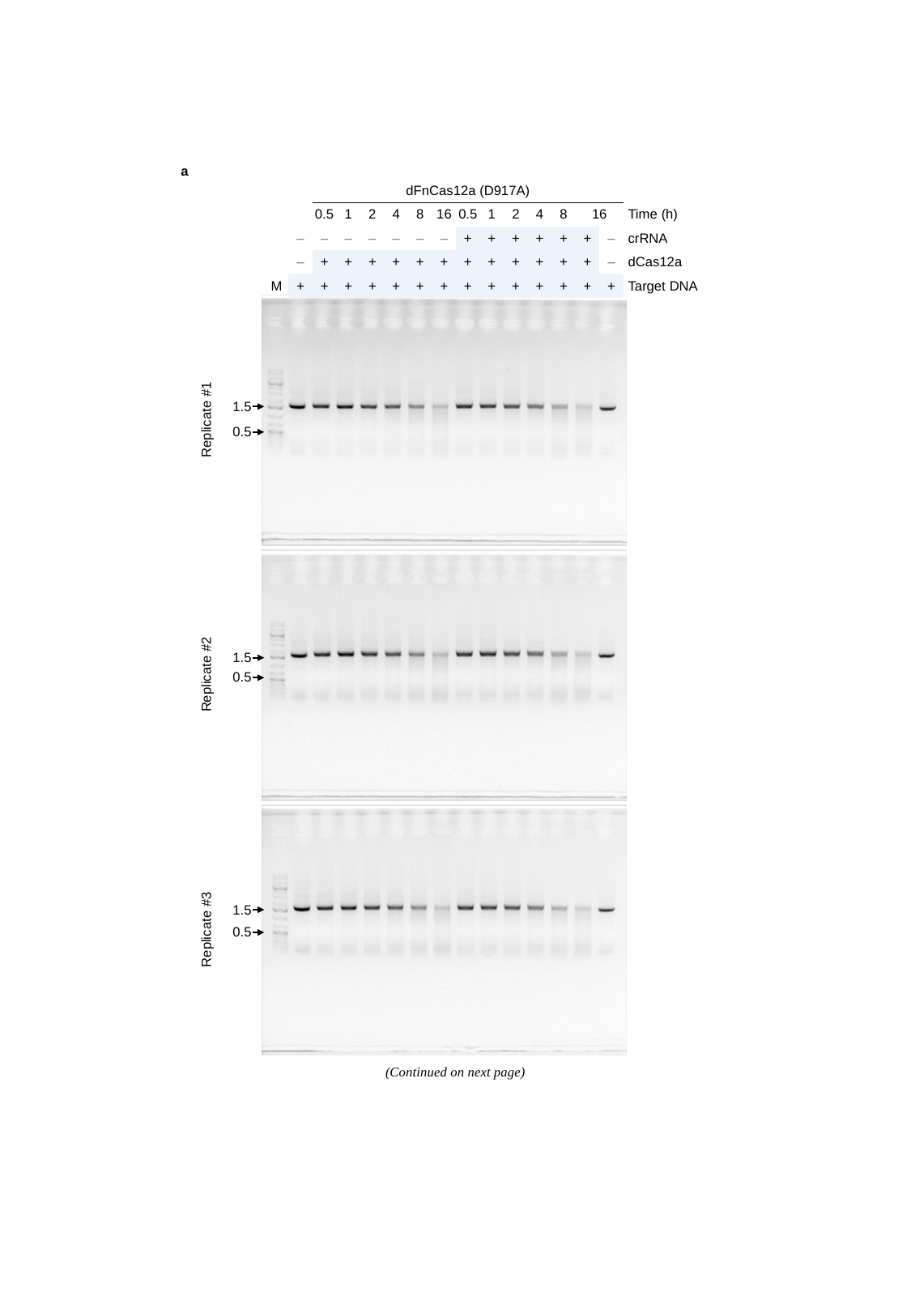

a
| | | dFnCas12a (D917A) | | | | | | | | | | | | | |
| --- | --- | --- | --- | --- | --- | --- | --- | --- | --- | --- | --- | --- | --- | --- | --- |
| | | 0.5 | 1 | 2 | 4 | 8 | 16 | 0.5 | 1 | 2 | 4 | 8 | 16 | | Time (h) |
| | – | – | – | – | – | – | – | + | + | + | + | + | + | – | crRNA |
| | – | + | + | + | + | + | + | + | + | + | + | + | + | – | dCas12a |
| M | + | + | + | + | + | + | + | + | + | + | + | + | + | + | Target DNA |
1.5
Replicate #1
0.5
1.5
Replicate #2
0.5
1.5
Replicate #3
0.5
(Continued on next page)

## Slide 17
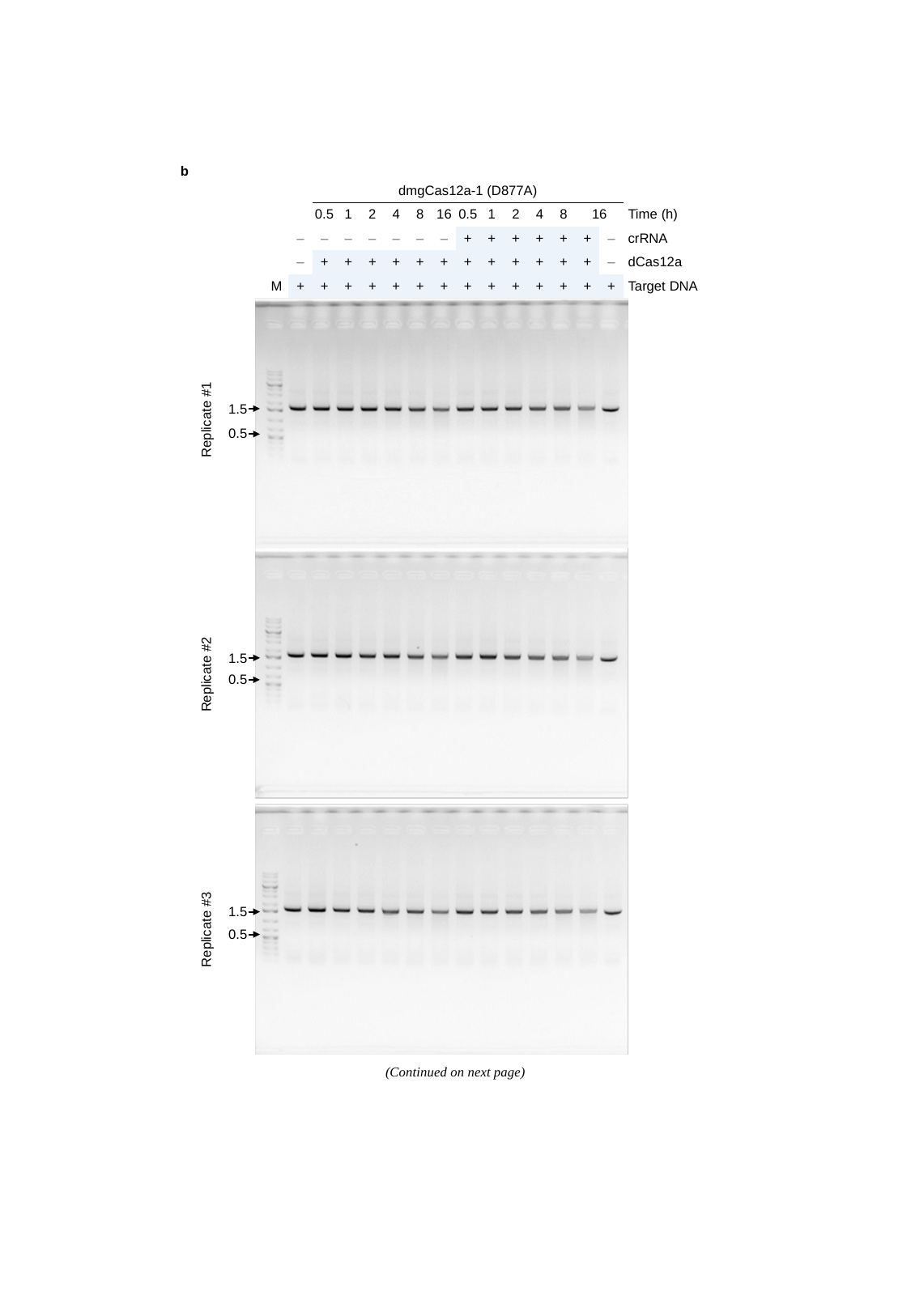

b
| | | dmgCas12a-1 (D877A) | | | | | | | | | | | | | |
| --- | --- | --- | --- | --- | --- | --- | --- | --- | --- | --- | --- | --- | --- | --- | --- |
| | | 0.5 | 1 | 2 | 4 | 8 | 16 | 0.5 | 1 | 2 | 4 | 8 | 16 | | Time (h) |
| | – | – | – | – | – | – | – | + | + | + | + | + | + | – | crRNA |
| | – | + | + | + | + | + | + | + | + | + | + | + | + | – | dCas12a |
| M | + | + | + | + | + | + | + | + | + | + | + | + | + | + | Target DNA |
1.5
Replicate #1
0.5
1.5
Replicate #2
0.5
1.5
Replicate #3
0.5
(Continued on next page)

## Slide 18
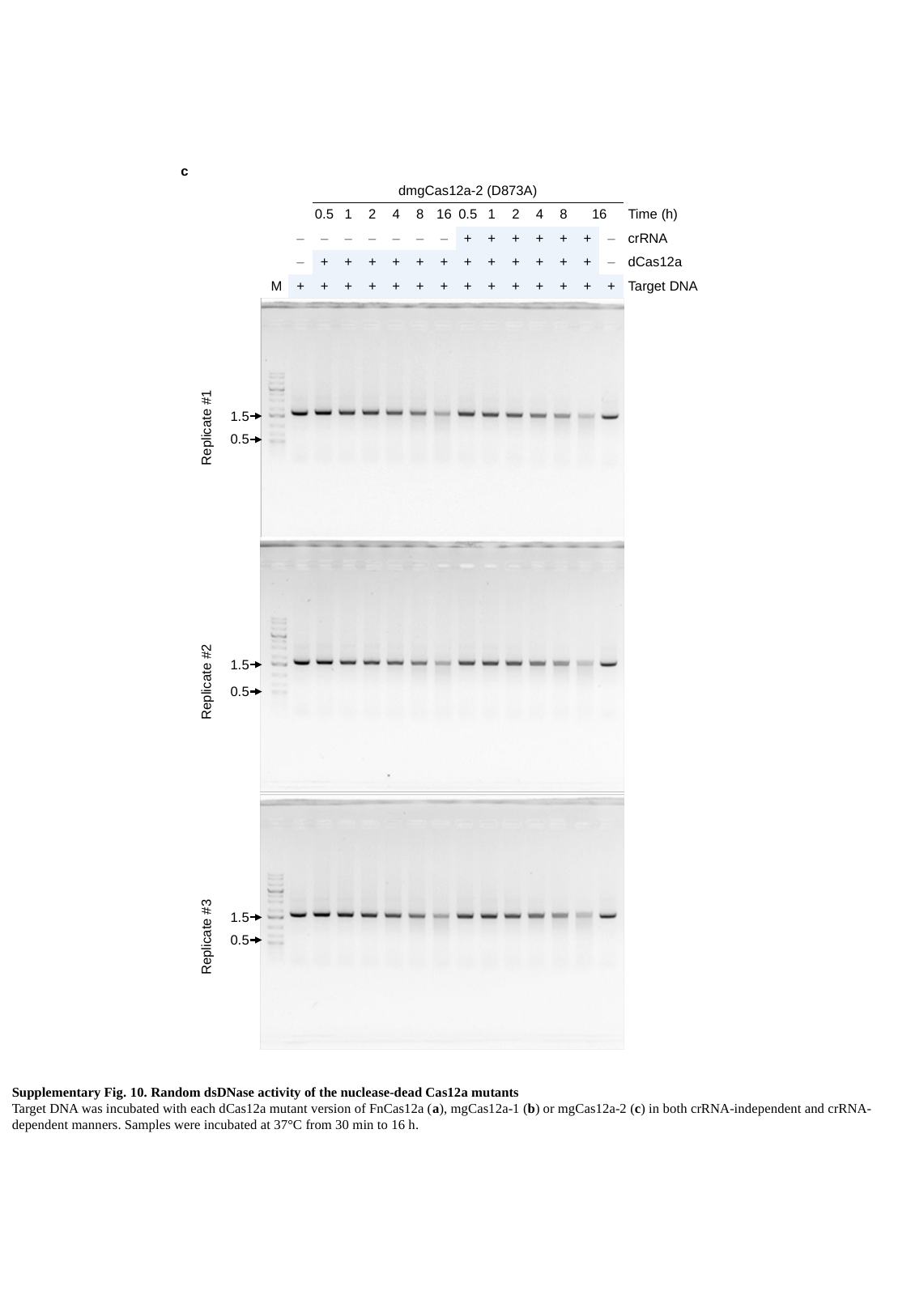

c
| | | dmgCas12a-2 (D873A) | | | | | | | | | | | | | |
| --- | --- | --- | --- | --- | --- | --- | --- | --- | --- | --- | --- | --- | --- | --- | --- |
| | | 0.5 | 1 | 2 | 4 | 8 | 16 | 0.5 | 1 | 2 | 4 | 8 | 16 | | Time (h) |
| | – | – | – | – | – | – | – | + | + | + | + | + | + | – | crRNA |
| | – | + | + | + | + | + | + | + | + | + | + | + | + | – | dCas12a |
| M | + | + | + | + | + | + | + | + | + | + | + | + | + | + | Target DNA |
1.5
Replicate #1
0.5
1.5
Replicate #2
0.5
1.5
Replicate #3
0.5
Supplementary Fig. 10. Random dsDNase activity of the nuclease-dead Cas12a mutants
Target DNA was incubated with each dCas12a mutant version of FnCas12a (a), mgCas12a-1 (b) or mgCas12a-2 (c) in both crRNA-independent and crRNA-dependent manners. Samples were incubated at 37°C from 30 min to 16 h.

## Slide 19
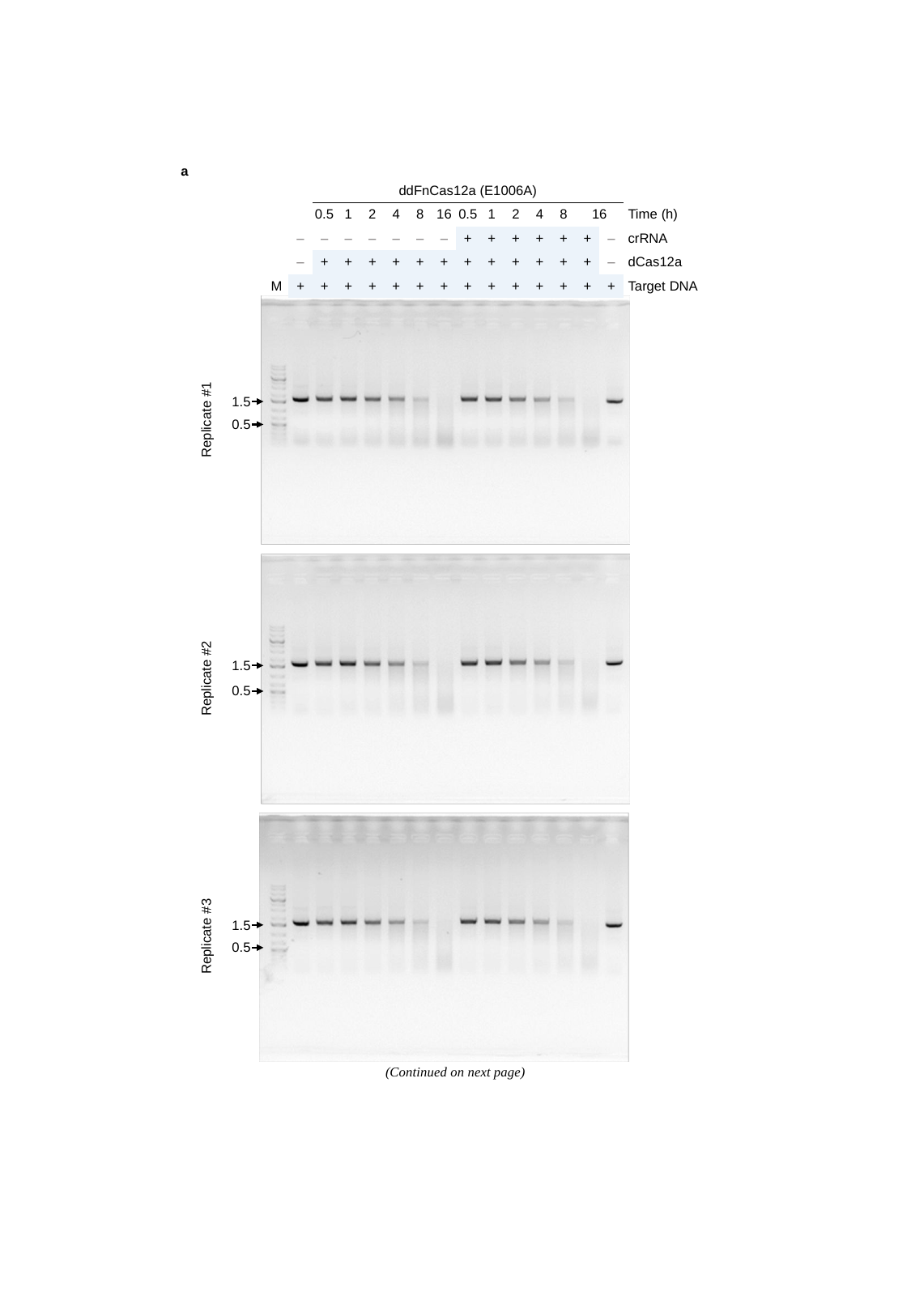

a
| | | ddFnCas12a (E1006A) | | | | | | | | | | | | | |
| --- | --- | --- | --- | --- | --- | --- | --- | --- | --- | --- | --- | --- | --- | --- | --- |
| | | 0.5 | 1 | 2 | 4 | 8 | 16 | 0.5 | 1 | 2 | 4 | 8 | 16 | | Time (h) |
| | – | – | – | – | – | – | – | + | + | + | + | + | + | – | crRNA |
| | – | + | + | + | + | + | + | + | + | + | + | + | + | – | dCas12a |
| M | + | + | + | + | + | + | + | + | + | + | + | + | + | + | Target DNA |
1.5
Replicate #1
0.5
1.5
Replicate #2
0.5
1.5
Replicate #3
0.5
(Continued on next page)

## Slide 20
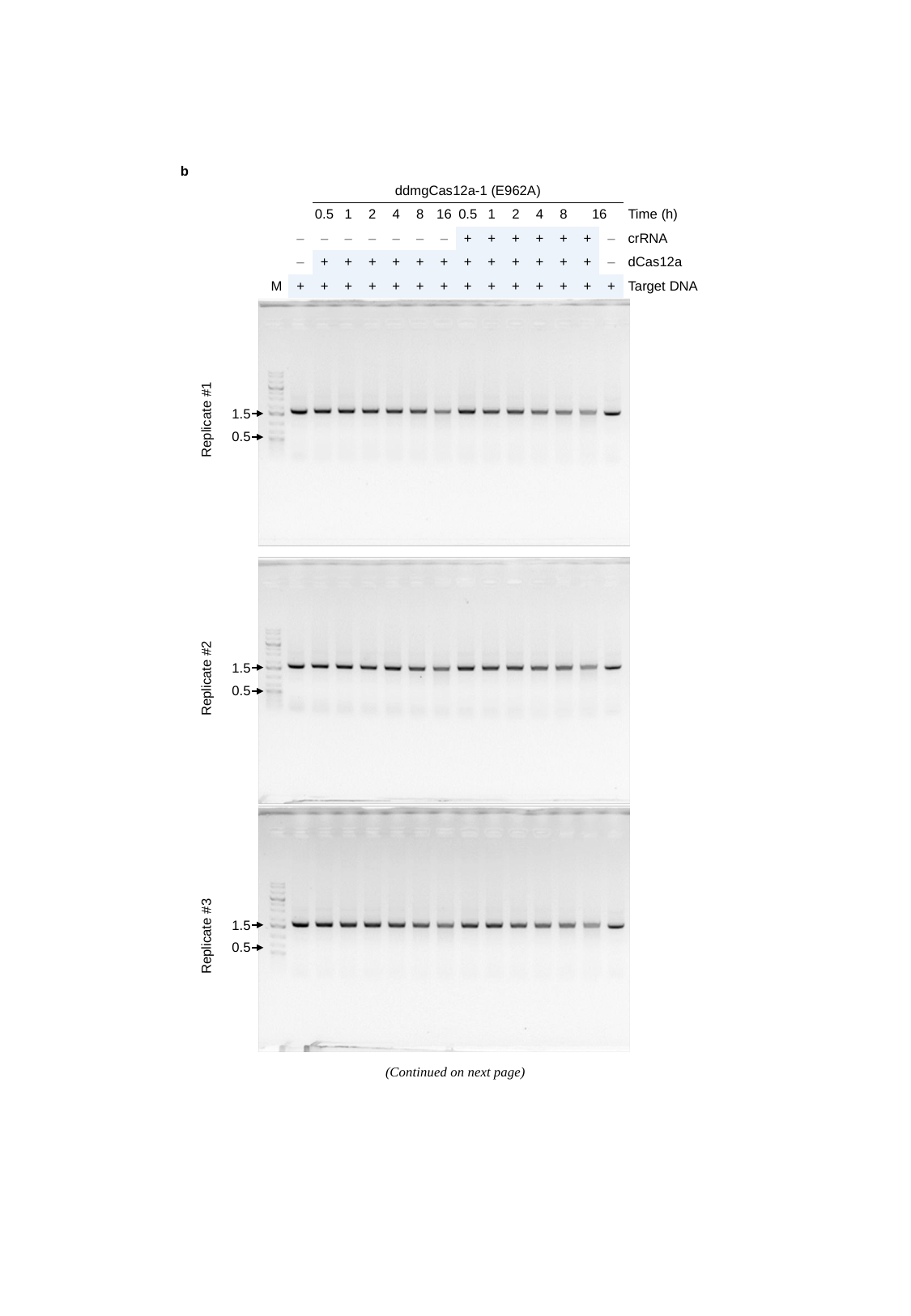

b
| | | ddmgCas12a-1 (E962A) | | | | | | | | | | | | | |
| --- | --- | --- | --- | --- | --- | --- | --- | --- | --- | --- | --- | --- | --- | --- | --- |
| | | 0.5 | 1 | 2 | 4 | 8 | 16 | 0.5 | 1 | 2 | 4 | 8 | 16 | | Time (h) |
| | – | – | – | – | – | – | – | + | + | + | + | + | + | – | crRNA |
| | – | + | + | + | + | + | + | + | + | + | + | + | + | – | dCas12a |
| M | + | + | + | + | + | + | + | + | + | + | + | + | + | + | Target DNA |
1.5
Replicate #1
0.5
1.5
Replicate #2
0.5
1.5
Replicate #3
0.5
(Continued on next page)

## Slide 21
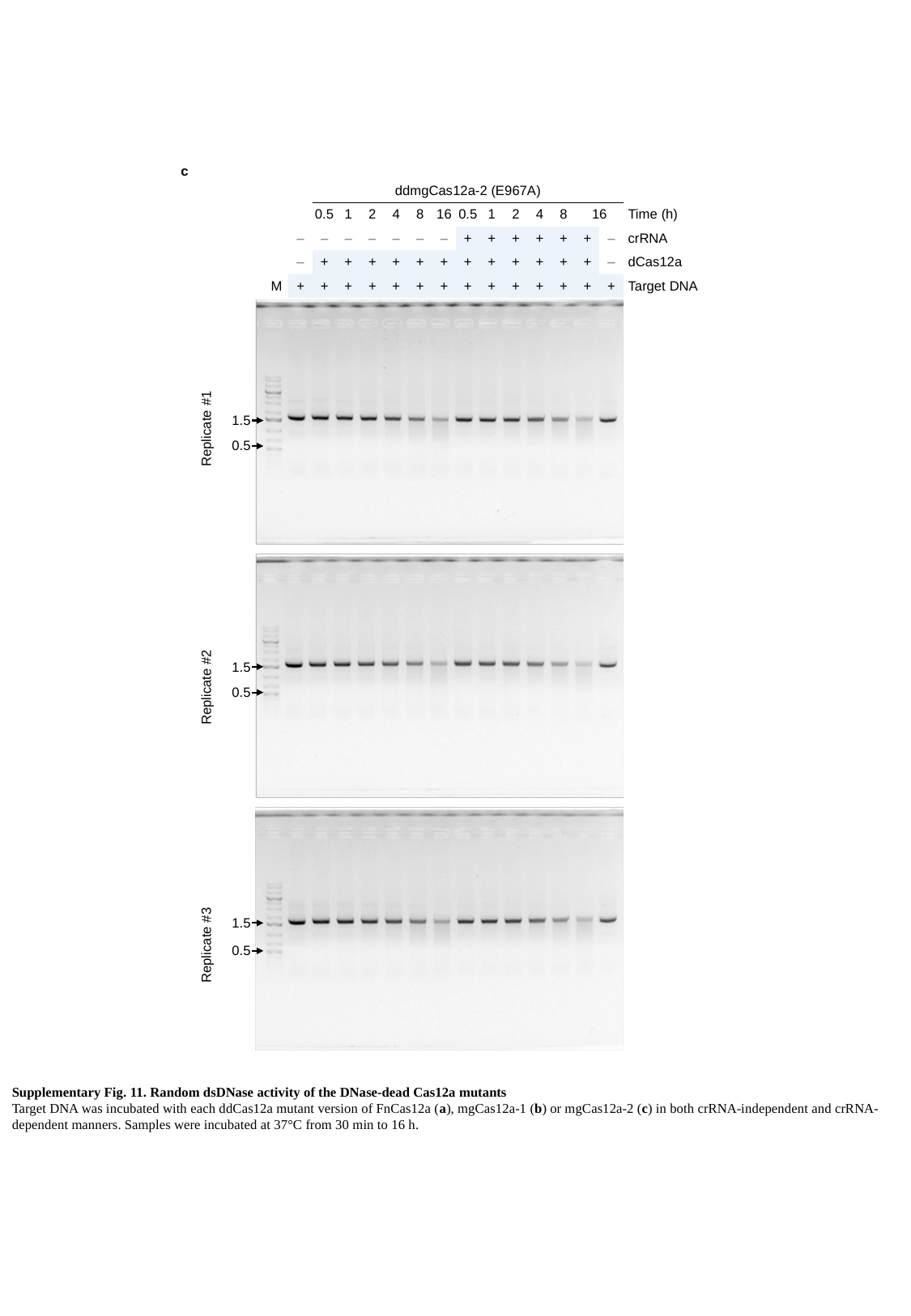

c
| | | ddmgCas12a-2 (E967A) | | | | | | | | | | | | | |
| --- | --- | --- | --- | --- | --- | --- | --- | --- | --- | --- | --- | --- | --- | --- | --- |
| | | 0.5 | 1 | 2 | 4 | 8 | 16 | 0.5 | 1 | 2 | 4 | 8 | 16 | | Time (h) |
| | – | – | – | – | – | – | – | + | + | + | + | + | + | – | crRNA |
| | – | + | + | + | + | + | + | + | + | + | + | + | + | – | dCas12a |
| M | + | + | + | + | + | + | + | + | + | + | + | + | + | + | Target DNA |
1.5
Replicate #1
0.5
1.5
Replicate #2
0.5
1.5
Replicate #3
0.5
Supplementary Fig. 11. Random dsDNase activity of the DNase-dead Cas12a mutants
Target DNA was incubated with each ddCas12a mutant version of FnCas12a (a), mgCas12a-1 (b) or mgCas12a-2 (c) in both crRNA-independent and crRNA-dependent manners. Samples were incubated at 37°C from 30 min to 16 h.

## Slide 22
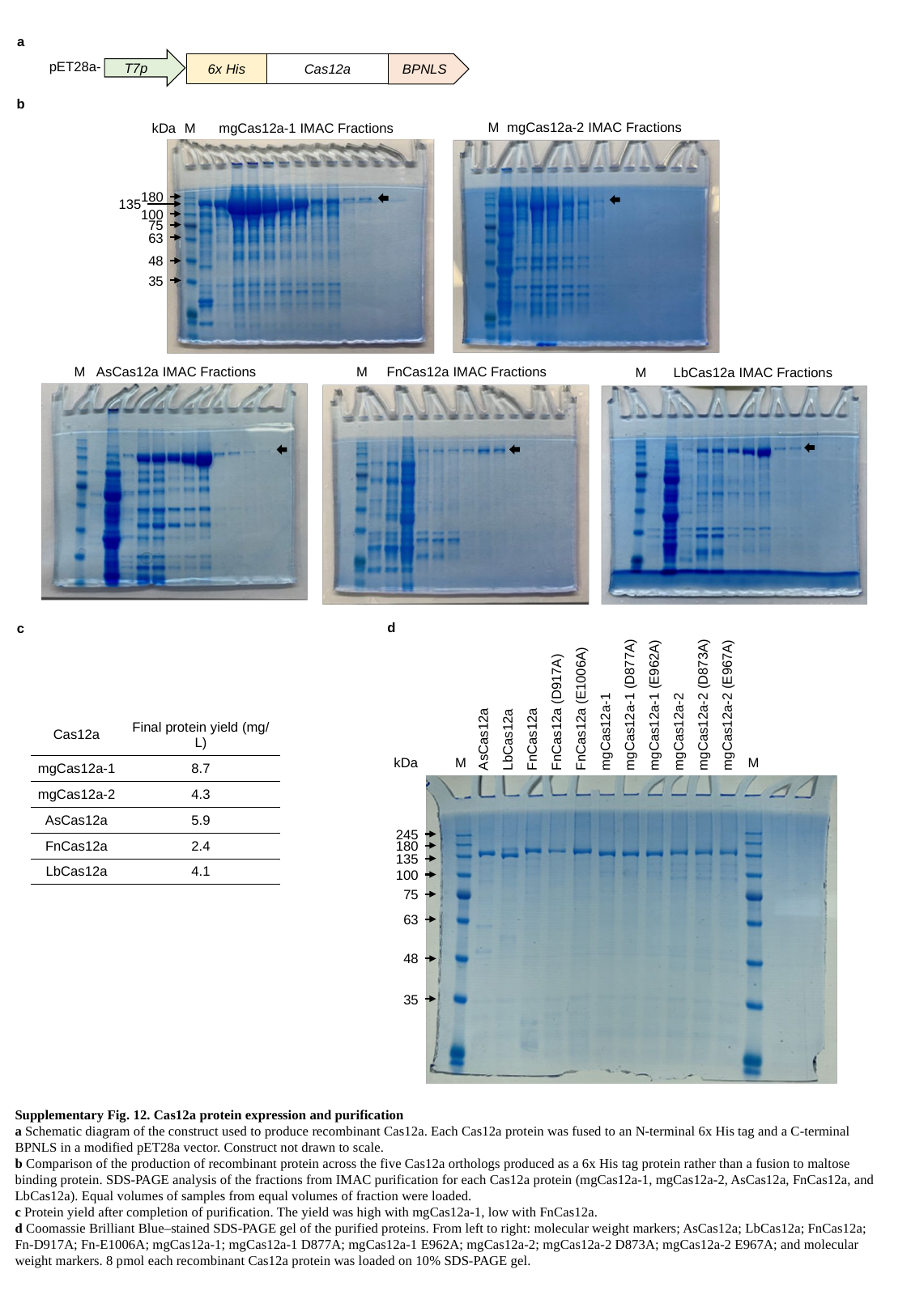

a
T7p
6x His
Cas12a
BPNLS
pET28a-
b
 M mgCas12a-2 IMAC Fractions
kDa
 M mgCas12a-1 IMAC Fractions
180
135
100
75
63
48
35
 M AsCas12a IMAC Fractions
 M FnCas12a IMAC Fractions
 M LbCas12a IMAC Fractions
d
c
| kDa | M | AsCas12a | LbCas12a | FnCas12a | FnCas12a (D917A) | FnCas12a (E1006A) | mgCas12a-1 | mgCas12a-1 (D877A) | mgCas12a-1 (E962A) | mgCas12a-2 | mgCas12a-2 (D873A) | mgCas12a-2 (E967A) | M |
| --- | --- | --- | --- | --- | --- | --- | --- | --- | --- | --- | --- | --- | --- |
| Cas12a | Final protein yield (mg/L) |
| --- | --- |
| mgCas12a-1 | 8.7 |
| mgCas12a-2 | 4.3 |
| AsCas12a | 5.9 |
| FnCas12a | 2.4 |
| LbCas12a | 4.1 |
245
180
135
100
75
63
48
35
Supplementary Fig. 12. Cas12a protein expression and purification
a Schematic diagram of the construct used to produce recombinant Cas12a. Each Cas12a protein was fused to an N-terminal 6x His tag and a C-terminal BPNLS in a modified pET28a vector. Construct not drawn to scale.
b Comparison of the production of recombinant protein across the five Cas12a orthologs produced as a 6x His tag protein rather than a fusion to maltose binding protein. SDS-PAGE analysis of the fractions from IMAC purification for each Cas12a protein (mgCas12a-1, mgCas12a-2, AsCas12a, FnCas12a, and LbCas12a). Equal volumes of samples from equal volumes of fraction were loaded.
c Protein yield after completion of purification. The yield was high with mgCas12a-1, low with FnCas12a.
d Coomassie Brilliant Blue–stained SDS-PAGE gel of the purified proteins. From left to right: molecular weight markers; AsCas12a; LbCas12a; FnCas12a; Fn-D917A; Fn-E1006A; mgCas12a-1; mgCas12a-1 D877A; mgCas12a-1 E962A; mgCas12a-2; mgCas12a-2 D873A; mgCas12a-2 E967A; and molecular weight markers. 8 pmol each recombinant Cas12a protein was loaded on 10% SDS-PAGE gel.
