# Supplementary material for: Biochemical characterization of the two novel mgCas12a proteins from the human gut metagenome
Source: Sci Rep. 2022 Dec 2;12:20857. doi: 10.1038/s41598-022-25227-w (PMC9718762; doi:10.1038/s41598-022-25227-w)
Supplement: Supplementary file 2 — Supplementary Tables. [file 41598_2022_25227_MOESM2_ESM.docx]

**Supplementary Tables for****Biochemical characterization of the two novel mgCas12a proteins from the human gut metagenome**Han Seong Kim^1,2^, Dong-wook Kim^1,2^, Sungjin Kim^3^, Sunghwa Choe^1,2,3,^*

^1^ School of Biological Sciences, College of Natural Sciences, Seoul National University, Seoul 08826, Korea
^2^ G+FLAS Life Sciences, 38 Nakseongdae-ro, Gwanak-Gu, Seoul 08790, Korea

^3^ Naturegenic Inc.,1281 Win Hentschel Boulevard, West Lafayette, IN 47906, USA

*To whom correspondence should be addressed. Tel: +82 2 880 6691; Fax: +82 2 872 1993; Email: [shchoe@snu.ac.kr](mailto:shchoe@snu.ac.kr)

**Supplementary Table 1.** Amino acid sequences of reference Cas12a variants used in this paper.

|  | Amino acid sequence (N to C terminus) |
| --- | --- |
| AsCas12a (WP_021736722.1) | MTQFEGFTNLYQVSKTLRFELIPQGKTLKHIQEQGFIEEDKARNDHYKELKPIIDRIYKTYADQCLQLVQLDWENLSAAIDSYRKEKTEETRNALIEEQATYRNAIHDYFIGRTDNLTDAINKRHAEIYKGLFKAELFNGKVLKQLGTVTTTEHENALLRSFDKFTTYFSGFYENRKNVFSAEDISTAIPHRIVQDNFPKFKENCHIFTRLITAVPSLREHFENVKKAIGIFVSTSIEEVFSFPFYNQLLTQTQIDLYNQLLGGISREAGTEKIKGLNEVLNLAIQKNDETAHIIASLPHRFIPLFKQILSDRNTLSFILEEFKSDEEVIQSFCKYKTLLRNENVLETAEALFNELNSIDLTHIFISHKKLETISSALCDHWDTLRNALYERRISELTGKITKSAKEKVQRSLKHEDINLQEIISAAGKELSEAFKQKTSEILSHAHAALDQPLPTTLKKQEEKEILKSQLDSLLGLYHLLDWFAVDESNEVDPEFSARLTGIKLEMEPSLSFYNKARNYATKKPYSVEKFKLNFQMPTLASGWDVNKEKNNGAILFVKNGLYYLGIMPKQKGRYKALSFEPTEKTSEGFDKMYYDYFPDAAKMIPKCSTQLKAVTAHFQTHTTPILLSNNFIEPLEITKEIYDLNNPEKEPKKFQTAYAKKTGDQKGYREALCKWIDFTRDFLSKYTKTTSIDLSSLRPSSQYKDLGEYYAELNPLLYHISFQRIAEKEIMDAVETGKLYLFQIYNKDFAKGHHGKPNLHTLYWTGLFSPENLAKTSIKLNGQAELFYRPKSRMKRMAHRLGEKMLNKKLKDQKTPIPDTLYQELYDYVNHRLSHDLSDEARALLPNVITKEVSHEIIKDRRFTSDKFFFHVPITLNYQAANSPSKFNQRVNAYLKEHPETPIIGIDRGERNLIYITVIDSTGKILEQRSLNTIQQFDYQKKLDNREKERVAARQAWSVVGTIKDLKQGYLSQVIHEIVDLMIHYQAVVVLENLNFGFKSKRTGIAEKAVYQQFEKMLIDKLNCLVLKDYPAEKVGGVLNPYQLTDQFTSFAKMGTQSGFLFYVPAPYTSKIDPLTGFVDPFVWKTIKNHESRKHFLEGFDFLHYDVKTGDFILHFKMNRNLSFQRGLPGFMPAWDIVFEKNETQFDAKGTPFIAGKRIVPVIENHRFTGRYRDLYPANELIALLEEKGIVFRDGSNILPKLLENDDSHAIDTMVALIRSVLQMRNSNAATGEDYINSPVRDLNGVCFDSRFQNPEWPMDADANGAYHIALKGQLLLNHLKESKDLKLQNGISNQDWLAYIQELRN* |
| LbCas12a (WP_035635841.1) | MSKLEKFTNCYSLSKTLRFKAIPVGKTQENIDNKRLLVEDEKRAEDYKGVKKLLDRYYLSFINDVLHSIKLKNLNNYISLFRKKTRTEKENKELENLEINLRKEIAKAFKGNEGYKSLFKKDIIETILPEFLDDKDEIALVNSFNGFTTAFTGFFDNRENMFSEEAKSTSIAFRCINENLTRYISNMDIFEKVDAIFDKHEVQEIKEKILNSDYDVEDFFEGEFFNFVLTQEGIDVYNAIIGGFVTESGEKIKGLNEYINLYNQKTKQKLPKFKPLYKQVLSDRESLSFYGEGYTSDEEVLEVFRNTLNKNSEIFSSIKKLEKLFKNFDEYSSAGIFVKNGPAISTISKDIFGEWNVIRDKWNAEYDDIHLKKKAVVTEKYEDDRRKSFKKIGSFSLEQLQEYADADLSVVEKLKEIIIQKVDEIYKVYGSSEKLFDADFVLEKSLKKNDAVVAIMKDLLDSVKSFENYIKAFFGEGKETNRDESFYGDFVLAYDILLKVDHIYDAIRNYVTQKPYSKDKFKLYFQNPQFMGGWDKDKETDYRATILRYGSKYYLAIMDKKYAKCLQKIDKDDVNGNYEKINYKLLPGPNKMLPKVFFSKKWMAYYNPSEDIQKIYKNGTFKKGDMFNLNDCHKLIDFFKDSISRYPKWSNAYDFNFSETEKYKDIAGFYREVEEQGYKVSFESASKKEVDKLVEEGKLYMFQIYNKDFSDKSHGTPNLHTMYFKLLFDENNHGQIRLSGGAELFMRRASLKKEELVVHPANSPIANKNPDNPKKTTTLSYDVYKDKRFSEDQYELHIPIAINKCPKNIFKINTEVRVLLKHDDNPYVIGIDRGERNLLYIVVVDGKGNIVEQYSLNEIINNFNGIRIKTDYHSLLDKKEKERFEARQNWTSIENIKELKAGYISQVVHKICELVEKYDAVIALEDLNSGFKNSRVKVEKQVYQKFEKMLIDKLNYMVDKKSNPCATGGALKGYQITNKFESFKSMSTQNGFIFYIPAWLTSKIDPSTGFVNLLKTKYTSIADSKKFISSFDRIMYVPEEDLFEFALDYKNFSRTDADYIKKWKLYSYGNRIRIFRNPKKNNVFDWEEVCLTSAYKELFNKYGINYQQGDIRALLCEQSDKAFYSSFMALMSLMLQMRNSITGRTDVDFLISPVKNSDGIFYDSRNYEAQENAILPKNADANGAYNIARKVLWAIGQFKKAEDEKLDKVKIAISNKEWLEYAQTSVKH* |
| FnCas12a (ABK90267.1) | MSIYQEFVNKYSLSKTLRFELIPQGKTLENIKARGLILDDEKRAKDYKKAKQIIDKYHQFFIEEILSSVCISEDLLQNYSDVYFKLKKSDDDNLQKDFKSAKDTIKKQISEYIKDSEKFKNLFNQNLIDAKKGQESDLILWLKQSKDNGIELFKANSDITDIDEALEIIKSFKGWTTYFKGFHENRKNVYSSNDIPTSIIYRIVDDNLPKFLENKAKYESLKDKAPEAINYEQIKKDLAEELTFDIDYKTSEVNQRVFSLDEVFEIANFNNYLNQSGITKFNTIIGGKFVNGENTKRKGINEYINLYSQQINDKTLKKYKMSVLFKQILSDTESKSFVIDKLEDDSDVVTTMQSFYEQIAAFKTVEEKSIKETLSLLFDDLKAQKLDLSKIYFKNDKSLTDLSQQVFDDYSVIGTAVLEYITQQIAPKNLDNPSKKEQELIAKKTEKAKYLSLETIKLALEEFNKHRDIDKQCRFEEILANFAAIPMIFDEIAQNKDNLAQISIKYQNQGKKDLLQASAEDDVKAIKDLLDQTNNLLHKLKIFHISQSEDKANILDKDEHFYLVFEECYFELANIVPLYNKIRNYITQKPYSDEKFKLNFENSTLANGWDKNKEPDNTAILFIKDDKYYLGVMNKKNNKIFDDKAIKENKGEGYKKIVYKLLPGANKMLPKVFFSAKSIKFYNPSEDILRIRNHSTHTKNGSPQKGYEKFEFNIEDCRKFIDFYKQSISKHPEWKDFGFRFSDTQRYNSIDEFYREVENQGYKLTFENISESYIDSVVNQGKLYLFQIYNKDFSAYSKGRPNLHTLYWKALFDERNLQDVVYKLNGEAELFYRKQSIPKKITHPAKEAIANKNKDNPKKESVFEYDLIKDKRFTEDKFFFHCPITINFKSSGANKFNDEINLLLKEKANDVHILSIDRGERHLAYYTLVDGKGNIIKQDTFNIIGNDRMKTNYHDKLAAIEKDRDSARKDWKKINNIKEMKEGYLSQVVHEIAKLVIEYNAIVVFEDLNFGFKRGRFKVEKQVYQKLEKMLIEKLNYLVFKDNEFDKTGGVLRAYQLTAPFETFKKMGKQTGIIYYVPAGFTSKICPVTGFVNQLYPKYESVSKSQEFFSKFDKICYNLDKGYFEFSFDYKNFGDKAAKGKWTIASFGSRLINFRNSDKNHNWDTREVYPTKELEKLLKDYSIEYGHGECIKAAICGESDKKFFAKLTSVLNTILQMRNSKTGTELDYLISPVADVNGNFFDSRQAPKNMPQDADANGAYHIGLKGLMLLGRIKNNQEGKKLNLVIKNEEYFEFVQNRNN* |
| Lb3Cas12a (WP_044910712.1) | MDYGNGQFERRAPLTKTITLRLKPIGETRETIREQKLLEQDAAFRKLVETVTPIVDDCIRKIADNALCHFGTEYDFSCLGNAISKNDSKAIKKETEKVEKLLAKVLTENLPDGLRKVNDINSAAFIQDTLTSFVQDDADKRVLIQELKGKTVLMQRFLTTRITALTVWLPDRVFENFNIFIENAEKMRILLDSPLNEKIMKFDPDAEQYASLEFYGQCLSQKDIDSYNLIISGIYADDEVKNPGINEIVKEYNQQIRGDKDESPLPKLKKLHKQILMPVEKAFFVRVLSNDSDARSILEKILKDTEMLPSKIIEAMKEADAGDIAVYGSRLHELSHVIYGDHGKLSQIIYDKESKRISELMETLSPKERKESKKRLEGLEEHIRKSTYTFDELNRYAEKNVMAAYIAAVEESCAEIMRKEKDLRTLLSKEDVKIRGNRHNTLIVKNYFNAWTVFRNLIRILRRKSEAEIDSDFYDVLDDSVEVLSLTYKGENLCRSYITKKIGSDLKPEIATYGSALRPNSRWWSPGEKFNVKFHTIVRRDGRLYYFILPKGAKPVELEDMDGDIECLQMRKIPNPTIFLPKLVFKDPEAFFRDNPEADEFVFLSGMKAPVTITRETYEAYRYKLYTVGKLRDGEVSEEEYKRALLQVLTAYKEFLENRMIYADLNFGFKDLEEYKDSSEFIKQVETHNTFMCWAKVSSSQLDDLVKSGNGLLFEIWSERLESYYKYGNEKVLRGYEGVLLSILKDENLVSMRTLLNSRPMLVYRPKESSKPMVVHRDGSRVVDRFDKDGKYIPPEVHDELYRFFNNLLIKEKLGEKARKILDNKKVKVKVLESERVKWSKFYDEQFAVTFSVKKNADCLDTTKDLNAEVMEQYSESNRLILIRNTTDILYYLVLDKNGKVLKQRSLNIINDGARDVDWKERFRQVTKDRNEGYNEWDYSRTSNDLKEVYLNYALKEIAEAVIEYNAILIIEKMSNAFKDKYSFLDDVTFKGFETKLLAKLSDLHFRGIKDGEPCSFTNPLQLCQNDSNKILQDGVIFMVPNSMTRSLDPDTGFIFAINDHNIRTKKAKLNFLSKFDQLKVSSEGCLIMKYSGDSLPTHNTDNRVWNCCCNHPITNYDRETKKVEFIEEPVEELSRVLEENGIETDTELNKLNERENVPGKVVDAIYSLVLNYLRGTVSGVAGQRAVYYSPVTGKKYDISFIQAMNLNRKCDYYRIGSKERGEWTDFVAQLIN* |
| BpCas12a (WP_013282991.1) | MLLYENYTKRNQITKSLRLELRPQGKTLRNIKELNLLEQDKAIYALLERLKPVIDEGIKDIARDTLKNCELSFEKLYEHFLSGDKKAYAKESERLKKEIVKTLIKNLPEGIGKISEINSAKYLNGVLYDFIDKTHKDSEEKQNILSDILETKGYLALFSKFLTSRITTLEQSMPKRVIENFEIYAANIPKMQDALERGAVSFAIEYESICSVDYYNQILSQEDIDSYNRLISGIMDEDGAKEKGINQTISEKNIKIKSEHLEEKPFRILKQLHKQILEEREKAFTIDHIDSDEEVVQVTKEAFEQTKEQWENIKKINGFYAKDPGDITLFIVVGPNQTHVLSQLIYGEHDRIRLLLEEYEKNTLEVLPRRTKSEKARYDKFVNAVPKKVAKESHTFDGLQKMTGDDRLFILYRDELARNYMRIKEAYGTFERDILKSRRGIKGNRDVQESLVSFYDELTKFRSALRIINSGNDEKADPIFYNTFDGIFEKANRTYKAENLCRNYVTKSPADDARIMASCLGTPARLRTHWWNGEENFAINDVAMIRRGDEYYYFVLTPDVKPVDLKTKDETDAQIFVQRKGAKSFLGLPKALFKCILEPYFESPEHKNDKNCVIEEYVSKPLTIDRRAYDIFKNGTFKKTNIGIDGLTEEKFKDDCRYLIDVYKEFIAVYTRYSCFNMSGLKRADEYNDIGEFFSDVDTRLCTMEWIPVSFERINDMVDKKEGLLFLVRSMFLYNRPRKPYERTFIQLFSDSNMEHTSMLLNSRAMIQYRAASLPRRVTHKKGSILVALRDSNGEHIPMHIREAIYKMKNNFDISSEDFIMAKAYLAEHDVAIKKANEDIIRNRRYTEDKFFLSLSYTKNADISARTLDYINDKVEEDTQDSRMAVIVTRNLKDLTYVAVVDEKNNVLEEKSLNEIDGVNYRELLKERTKIKYHDKTRLWQYDVSSKGLKEAYVELAVTQISKLATKYNAVVVVESMSSTFKDKFSFLDEQIFKAFEARLCARMSDLSFNTIKEGEAGSISNPIQVSNNNGNSYQDGVIYFLNNAYTRTLCPDTGFVDVFDKTRLITMQSKRQFFAKMKDIRIDDGEMLFTFNLEEYPTKRLLDRKEWTVKIAGDGSYFDKDKGEYVYVNDIVREQIIPALLEDKAVFDGNMAEKFLDKTAISGKSVELIYKWFANALYGIITKKDGEKIYRSPITGTEIDVSKNTTYNFGKKFMFKQEYRGDGDFLDAFLNYMQAQDIAV* |
| PeCas12a (KKP36646.1) | MSNFFKNFTNLYELSKTLRFELKPVGDTLTNMKDHLEYDEKLQTFLKDQNIDDAYQALKPQFDEIHEEFITDSLESKKAKEIDFSEYLDLFQEKKELNDSEKKLRNKIGETFNKAGEKWKKEKYPQYEWKKGSKIANGADILSCQDMLQFIKYKNPEDEKIKNYIDDTLKGFFTYFGGFNQNRANYYETKKEASTAVATRIVHENLPKFCDNVIQFKHIIKRKKDGTVEKTERKTEYLNAYQYLKNNNKITQIKDAETEKMIESTPIAEKIFDVYYFSSCLSQKQIEEYNRIIGHYNLLINLYNQAKRSEGKHLSANEKKYKDLPKFKTLYKQIGCGKKKDLFYTIKCDTEEEANKSRNEGKESHSVEEIINKAQEAINKYFKSNNDCENINTVPDFINYILTKENYEGVYWSKAAMNTISDKYFANYHDLQDRLKEAKVFQKADKKSEDDIKIPEAIELSGLFGVLDSLADWQTTLFKSSILSNEDKLKIITDSQTPSEALLKMIFNDIEKNMESFLKETNDIITLKKYKGNKEGTEKIKQWFDYTLAINRMLKYFLVKENKIKGNSLDTNISEALKTLIYSDDAEWFKWYDALRNYLTQKPQDEAKENKLKLNFDNPSLAGGWDVNKECSNFCVILKDKNEKKYLAIMKKGENTLFQKEWTEGRGKNLTKKSNPLFEINNCEILSKMEYDFWADVSKMIPKCSTQLKAVVNHFKQSDNEFIFPIGYKVTSGEKFREECKISKQDFELNNKVFNKNELSVTAMRYDLSSTQEKQYIKAFQKEYWELLFKQEKRDTKLTNNEIFNEWINFCNKKYSELLSWERKYKDALTNWINFCKYFLSKYPKTTLFNYSFKESENYNSLDEFYRDVDICSYKLNINTTINKSILDRLVEEGKLYLFEIKNQDSNDGKSIGHKNNLHTIYWNAIFENFDNRPKLNGEAEIFYRKAISKDKLGIVKGKKTKNGTEIIKNYRFSKEKFILHVPITLNFCSNNEYVNDIVNTKFYNFSNLHFLGIDRGEKHLAYYSLVNKNGEIVDQGTLNLPFTDKDGNQRSIKKEKYFYNKQEDKWEAKEVDCWNYNDLLDAMASNRDMARKNWQRIGTIKEAKNGYVSLVIRKIADLAVNNERPAFIVLEDLNTGFKRSRQKIDKSVYQKFELALAKKLNFLVDKNAKRDEIGSPTKALQLTPPVNNYGDIENKKQAGIMLYTRANYTSQTDPATGWRKTIYLKAGPEETTYKKDGKIKNKSVKDQIIETFTDIGFDGKDYYFEYDKGEFVDEKTGEIKPKKWRLYSGENGKSLDRFRGEREKDKYEWKIDKIDIVKILDDLFVNFDKNISLLKQLKEGVELTRNNEHGTGESLRFAINLIQQIRNTGNNERDNDFILSPVRDENGKHFDSREYWDKETKGEKISMPSSGDANGAFNIARKGIIMNAHILANSDSKDLSLFVSDEEWDLHLNNKTEWKKQLNIFSSRKAMAKRKK* |
| PbCas12a (KKT48220.1) | MENIFDQFIGKYSLSKTLRFELKPVGKTEDFLKINKVFEKDQTIDDSYNQAKFYFDSLHQKFIDAALASDKTSELSFQNFADVLEKQNKIILDKKREMGALRKRDKNAVGIDRLQKEINDAEDIIQKEKEKIYKDVRTLFDNEAESWKTYYQEREVDGKKITFSKADLKQKGADFLTAAGILKVLKYEFPEEKEKEFQAKNQPSLFVEEKENPGQKRYIFDSFDKFAGYLTKFQQTKKNLYAADGTSTAVATRIADNFIIFHQNTKVFRDKYKNNHTDLGFDEENIFEIERYKNCLLQREIEHIKNENSYNKIIGRINKKIKEYRDQKAKDTKLTKSDFPFFKNLDKQILGEVEKEKQLIEKTREKTEEDVLIERFKEFIENNEERFTAAKKLMNAFCNGEFESEYEGIYLKNKAINTISRRWFVSDRDFELKLPQQKSKNKSEKNEPKVKKFISIAEIKNAVEELDGDIFKAVFYDKKIIAQGGSKLEQFLVIWKYEFEYLFRDIERENGEKLLGYDSCLKIAKQLGIFPQEKEAREKATAVIKNYADAGLGIFQMMKYFSLDDKDRKNTPGQLSTNFYAEYDGYYKDFEFIKYYNEFRNFITKKPFDEDKIKLNFENGALLKGWDENKEYDFMGVILKKEGRLYLGIMHKNHRKLFQSMGNAKGDNANRYQKMIYKQIADASKDVPRLLLTSKKAMEKFKPSQEILRIKKEKTFKRESKNFSLRDLHALIEYYRNCIPQYSNWSFYDFQFQDTGKYQNIKEFTDDVQKYGYKISFRDIDDEYINQALNEGKMYLFEVVNKDIYNTKNGSKNLHTLYFEHILSAENLNDPVFKLSGMAEIFQRQPSVNEREKITTQKNQCILDKGDRAYKYRRYTEKKIMFHMSLVLNTGKGEIKQVQFNKIINQRISSSDNEMRVNVIGIDRGEKNLLYYSVVKQNGEIIEQASLNEINGVNYRDKLIEREKERLKNRQSWKPVVKIKDLKKGYISHVIHKICQLIEKYSAIVVLEDLNMRFKQIRGGIERSVYQQFEKALIDKLGYLVFKDNRDLRAPGGVLNGYQLSAPFVSFEKMRKQTGILFYTQAEYTSKTDPITGFRKNVYISNSASLDKIKEAVKKFDAIGWDGKEQSYFFKYNPYNLADEKYKNSTVSKEWAIFASAPRIRRQKGEDGYWKYDRVKVNEEFEKLLKVWNFVNPKATDIKQEIIKKEKAGDLQGEKELDGRLRNFWHSFIYLFNLVLELRNSFSLQIKIKAGEVIAVDEGVDFIASPVKPFFTTPNPYIPSNLCWLAVENADANGAYNIARKGVMILKKIREHAKKDPEFKKLPNLFISNAEWDEAARDWGKYAGTTALNLDH* |
| SsCas12a (WP_039658684.1) | MQTLFENFTNQYPVSKTLRFELIPQGKTKDFIEQKGLLKKDEDRAEKYKKVKNIIDEYHKDFIEKSLNGLKLDGLEKYKTLYLKQEKDDKDKKAFDKEKENLRKQIANAFRNNEKFKTLFAKELIKNDLMSFACEEDKKNVKEFEAFTTYFTGFHQNRANMYVADEKRTAIASRLIHENLPKFIDNIKIFEKMKKEAPELLSPFNQTLKDMKDVIKGTTLEEIFSLDYFNKTLTQSGIDIYNSVIGGRTPEEGKTKIKGLNEYINTDFNQKQTDKKKRQPKFKQLYKQILSDRQSLSFIAEAFKNDTEILEAIEKFYVNELLHFSNEGKSTNVLDAIKNAVSNLESFNLTKMYFRSGASLTDVSRKVFGEWSIINRALDNYYATTYPIKPREKSEKYEERKEKWLKQDFNVSLIQTAIDEYDNETVKGKNSGKVIADYFAKFCDDKETDLIQKVNEGYIAVKDLLNTPCPENEKLGSNKDQVKQIKAFMDSIMDIMHFVRPLSLKDTDKEKDETFYSLFTPLYDHLTQTIALYNKVRNYLTQKPYSTEKIKLNFENSTLLGGWDLNKETDNTAIILRKDNLYYLGIMDKRHNRIFRNVPKADKKDFCYEKMVYKLLPGANKMLPKVFFSQSRIQEFTPSAKLLENYANETHKKGDNFNLNHCHKLIDFFKDSINKHEDWKNFDFRFSATSTYADLSGFYHEVEHQGYKISFQSVADSFIDDLVNEGKLYLFQIYNKDFSPFSKGKPNLHTLYWKMLFDENNLKDVVYKLNGEAEVFYRKKSIAEKNTTIHKANESIINKNPDNPKATSTFNYDIVKDKRYTIDKFQFHIPITMNFKAEGIFNMNQRVNQFLKANPDINIIGIDRGERHLLYYALINQKGKILKQDTLNVIANEKQKVDYHNLLDKKEGDRATARQEWGVIETIKELKEGYLSQVIHKLTDLMIENNAIIVMEDLNFGFKRGRQKVEKQVYQKFEKMLIDKLNYLVDKNKKANELGGLLNAFQLANKFESFQKMGKQNGFIFYVPAWNTSKTDPATGFIDFLKPRYENLNQAKDFFEKFDSIRLNSKADYFEFAFDFKNFTEKADGGRTKWTVCTTNEDRYAWNRALNNNRGSQEKYDITAELKSLFDGKVDYKSGKDLKQQIASQESADFFKALMKNLSITLSLRHNNGEKGDNEQDYILSPVADSKGRFFDSRKADDDMPKNADANGAYHIALKGLWCLEQISKTDDLKKVKLAISNKEWLEFVQTLKG* |
| CMtCas12a (AIZ56868.1) | MNNYDEFTKLYPIQKTIRFELKPQGRTMEHLETFNFFEEDRDRAEKYKILKEAIDEYHKKFIDEHLTNMSLDWNSLKQISEKYYKSREEKDKKVFLSEQKRMRQEIVSEFKKDDRFKDLFSKKLFSELLKEEIYKKGNHQEIDALKSFDKFSGYFIGLHENRKNMYSDGDEITAISNRIVNENFPKFLDNLQKYQEARKKYPEWIIKAESALVAHNIKMDEVFSLEYFNKVLNQEGIQRYNLALGGYVTKSGEKMMGLNDALNLAHQSEKSSKGRIHMTPLFKQILSEKESFSYIPDVFTEDSQLLPSIGGFFAQIENDKDGNIFDRALELISSYAEYDTERIYIRQADINRVSNVIFGEWGTLGGLMREYKADSINDINLERTCKKVDKWLDSKEFALSDVLEAIKRTGNNDAFNEYISKMRTAREKIDAARKEMKFISEKISGDEESIHIIKTLLDSVQQFLHFFNLFKARQDIPLDGAFYAEFDEVHSKLFAIVPLYNKVRNYLTKNNLNTKKIKLNFKNPTLANGWDQNKVYDYASLIFLRDGNYYLGIINPKRKKNIKFEQGSGNGPFYRKMVYKQIPGPNKNLPRVFLTSTKGKKEYKPSKEIIEGYEADKHIRGDKFDLDFCHKLIDFFKESIEKHKDWSKFNFYFSPTESYGDISEFYLDVEKQGYRMHFENISAETIDEYVEKGDLFLFQIYNKDFVKAATGKKDMHTIYWNAAFSPENLQDVVVKLNGEAELFYRDKSDIKEIVHREGEILVNRTYNGRTPVPDKIHKKLTDYHNGRTKDLGEAKEYLDKVRYFKAHYDITKDRRYLNDKIYFHVPLTLNFKANGKKNLNKMVIEKFLSDEKAHIIGIDRGERNLLYYSIIDRSGKIIDQQSLNVIDGFDYREKLNQREIEMKDARQSWNAIGKIKDLKEGYLSKAVHEITKMAIQYNAIVVMEELNYGFKRGRFKVEKQIYQKFENMLIDKMNYLVFKDAPDESPGGVLNAYQLTNPLESFAKLGKQTGILFYVPAAYTSKIDPTTGFVNLFNTSSKTNAQERKEFLQKFESISYSAKDGGIFAFAFDYRKFGTSKTDHKNVWTAYTNGERMRYIKEKKRNELFDPSKEIKEALTSSGIKYDGGQNILPDILRSNNNGLIYTMYSSFIAAIQMRVYDGKEDYIISPIKNSKGEFFRTDPKRRELPIDADANGAYNIALRGELTMRAIAEKFDPDSEKMAKLELKHKDWFEFMQTRGD* |
| EeCas12a (WP_012739647.1) | MNGNRSIVYREFVGVIPVAKTLRNELRPVGHTQEHIIQNGLIQEDELRQEKSTELKNIMDDYYREYIDKSLSGVTDLDFTLLFELMNLVQSSPSKDNKKALEKEQSKMREQICTHLQSDSNYKNIFNAKLLKEILPDFIKNYNQYDVKDKAGKLETLALFNGFSTYFTDFFEKRKNVFTKEAVSTSIAYRIVHENSLIFLANMTSYKKISEKALDEIEVIEKNNQDKMGDWELNQIFNPDFYNMVLIQSGIDFYNEICGVVNAHMNLYCQQTKNNYNLFKMRKLHKQILAYTSTSFEVPKMFEDDMSVYNAVNAFIDETEKGNIIGKLKDIVNKYDELDEKRIYISKDFYETLSCFMSGNWNLITGCVENFYDENIHAKGKSKEEKVKKAVKEDKYKSINDVNDLVEKYIDEKERNEFKNSNAKQYIREISNIITDTETAHLEYDDHISLIESEEKADEMKKRLDMYMNMYHWAKAFIVDEVLDRDEMFYSDIDDIYNILENIVPLYNRVRNYVTQKPYNSKKIKLNFQSPTLANGWSQSKEFDNNAIILIRDNKYYLAIFNAKNKPDKKIIQGNSDKKNDNDYKKMVYNLLPGANKMLPKVFLSKKGIETFKPSDYIISGYNAHKHIKTSENFDISFCRDLIDYFKNSIEKHAEWRKYEFKFSATDSYSDISEFYREVEMQGYRIDWTYISEADINKLDEEGKIYLFQIYNKDFAENSTGKENLHTMYFKNIFSEENLKDIIIKLNGQAELFYRRASVKNPVKHKKDSVLVNKTYKNQLDNGDVVRIPIPDDIYNEIYKMYNGYIKESDLSEAAKEYLDKVEVRTAQKDIVKDYRYTVDKYFIHTPITINYKVTARNNVNDMVVKYIAQNDDIHVIGIDRGERNLIYISVIDSHGNIVKQKSYNILNNYDYKKKLVEKEKTREYARKNWKSIGNIKELKEGYISGVVHEIAMLIVEYNAIIAMEDLNYGFKRGRFKVERQVYQKFESMLINKLNYFASKEKSVDEPGGLLKGYQLTYVPDNIKNLGKQCGVIFYVPAAFTSKIDPSTGFISAFNFKSISTNASRKQFFMQFDEIRYCAEKDMFSFGFDYNNFDTYNITMGKTQWTVYTNGERLQSEFNNARRTGKTKSINLTETIKLLLEDNEINYADGHDIRIDMEKMDEDKKSEFFAQLLSLYKLTVQMRNSYTEAEEQENGISYDKIISPVINDEGEFFDSDNYKESDDKECKMPKDADANGAYCIALKGLYEVLKIKSEWTEDGFDRNCLKLPHAEWLDFIQNKRYE* |
| MbCas12a (KDN25524.1) | MLFQDFTHLYPLSKTVRFELKPIDRTLEHIHAKNFLSQDETMADMHQKVKVILDDYHRDFIADMMGEVKLTKLAEFYDVYLKFRKNPKDDELQKQLKDLQAVLRKEIVKPIGNGGKYKAGYDRLFGAKLFKDGKELGDLAKFVIAQEGESSPKLAHLAHFEKFSTYFTGFHDNRKNMYSDEDKHTAIAYRLIHENLPRFIDNLQILTTIKQKHSALYDQIINELTASGLDVSLASHLDGYHKLLTQEGITAYNTLLGGISGEAGSPKIQGINELINSHHNQHCHKSERIAKLRPLHKQILSDGMSVSFLPSKFADDSEMCQAVNEFYRHYADVFAKVQSLFDGFDDHQKDGIYVEHKNLNELSKQAFGDFALLGRVLDGYYVDVVNPEFNERFAKAKTDNAKAKLTKEKDKFIKGVHSLASLEQAIEHYTARHDDESVQAGKLGQYFKHGLAGVDNPIQKIHNNHSTIKGFLERERPAGERALPKIKSGKNPEMTQLRQLKELLDNALNVAHFAKLLTTKTTLDNQDGNFYGEFGVLYDELAKIPTLYNKVRDYLSQKPFSTEKYKLNFGNPTLLNGWDLNKEKDNFGVILQKDGCYYLALLDKAHKKVFDNAPNTGKSIYQKMIYKYLEVRKQFPKVFFSKEAIAINYHPSKELVEIKDKGRQRSDDERLKLYRFILECLKIHPKYDKKFEGAIGDIQLFKKDKKGREVPISEKDLFDKINGIFSSKPKLEMEDFFIGEFKRYNPSQDLVDQYNIYKKIDSNDNRKKENFYNNHPKFKKDLVRYYYESMCKHEEWEESFEFSKKLQDIGCYVDVNELFTEIETRRLNYKISFCNINADYIDELVEQGQLYLFQIYNKDFSPKAHGKPNLHTLYFKALFSEDNLADPIYKLNGEAQIFYRKASLDMNETTIHRAGEVLENKNPDNPKKRQFVYDIIKDKRYTQDKFMLHVPITMNFGVQGMTIKEFNKKVNQSIQQYDEVNVIGIDRGERHLLYLTVINSKGEILEQCSLNDITTASANGTQMTTPYHKILDKREIERLNARVGWGEIETIKELKSGYLSHVVHQISQLMLKYNAIVVLEDLNFGFKRGRFKVEKQIYQNFENALIKKLNHLVLKDKADDEIGSYKNALQLTNNFTDLKSIGKQTGFLFYVPAWNTSKIDPETGFVDLLKPRYENIAQSQAFFGKFDKICYNADKDYFEFHIDYAKFTDKAKNSRQIWTICSHGDKRYVYDKTANQNKGAAKGINVNDELKSLFARHHINEKQPNLVMDICQNNDKEFHKSLMYLLKTLLALRYSNASSDEDFILSPVANDEGVFFNSALADDTQPQNADANGAYHIALKGLWLLNELKNSDDLNKVKLAIDNQTWLNFAQNR* |
| LiCas12a (WP_020988726.1) | MEDYSGFVNIYSIQKTLRFELKPVGKTLEHIEKKGFLKKDKIRAEDYKAVKKIIDKYHRAYIEEVFDSVLHQKKKKDKTRFSTQFIKEIKEFSELYYKTEKNIPDKERLEALSEKLRKMLVGAFKGEFSEEVAEKYKNLFSKELIRNEIEKFCETDEERKQVSNFKSFTTYFTGFHSNRQNIYSDEKKSTAIGYRIIHQNLPKFLDNLKIIESIQRRFKDFPWSDLKKNLKKIDKNIKLTEYFSIDGFVNVLNQKGIDAYNTILGGKSEESGEKIQGLNEYINLYRQKNNIDRKNLPNVKILFKQILGDRETKSFIPEAFPDDQSVLNSITEFAKYLKLDKKKKSIIAELKKFLSSFNRYELDGIYLANDNSLASISTFLFDDWSFIKKSVSFKYDESVGDPKKKIKSPLKYEKEKEKWLKQKYYTISFLNDAIESYSKSQDEKRVKIRLEAYFAEFKSKDDAKKQFDLLERIEEAYAIVEPLLGAEYPRDRNLKADKKEVGKIKDFLDSIKSLQFFLKPLLSAEIFDEKDLGFYNQLEGYYEEIDSIGHLYNKVRNYLTGKIYSKEKFKLNFENSTLLKGWDENREVANLCVIFREDQKYYLGVMDKENNTILSDIPKVKPNELFYEKMVYKLIPTPHMQLPRIIFSSDNLSIYNPSKSILKIREAKSFKEGKNFKLKDCHKFIDFYKESISKNEDWSRFDFKFSKTSSYENISEFYREVERQGYNLDFKKVSKFYIDSLVEDGKLYLFQIYNKDFSIFSKGKPNLHTIYFRSLFSKENLKDVCLKLNGEAEMFFRKKSINYDEKKKREGHHPELFEKLKYPILKDKRYSEDKFQFHLPISLNFKSKERLNFNLKVNEFLKRNKDINIIGIDRGERNLLYLVMINQKGEILKQTLLDSMQSGKGRPEINYKEKLQEKEIERDKARKSWGTVENIKELKEGYLSIVIHQISKLMVENNAIVVLEDLNIGFKRGRQKVERQVYQKFEKMLIDKLNFLVFKENKPTEPGGVLKAYQLTDEFQSFEKLSKQTGFLFYVPSWNTSKIDPRTGFIDFLHPAYENIEKAKQWINKFDSIRFNSKMDWFEFTADTRKFSENLMLGKNRVWVICTTNVERYFTSKTANSSIQYNSIQITEKLKELFVDIPFSNGQDLKPEILRKNDAVFFKSLLFYIKTTLSLRQNNGKKGEEEKDFILSPVVDSKGRFFNSLEASDDEPKDADANGAYHIALKGLMNLLVLNETKEENLSRPKWKIKNKDWLEFVWERNR* |
| PcCas12a (WP_036890108.1) | MDSLKDFTNLYPVSKTLRFELKPVGKTLENIEKAGILKEDEHRAESYRRVKKIIDTYHKVFIDSSLENMAKMGIENEIKAMLQSFCELYKKDHRTEGEDKALDKIRAVLRGLIVGAFTGVCGRRENTVQNEKYESLFKEKLIKEILPDFVLSTEAESLPFSVEEATRSLKEFDSFTSYFAGFYENRKNIYSTKPQSTAIAYRLIHENLPKFIDNILVFQKIKEPIAKELEHIRADFSAGGYIKKDERLEDIFSLNYYIHVLSQAGIEKYNALIGKIVTEGDGEMKGLNEHINLYNQQRGREDRLPLFRPLYKQILSDREQLSYLPESFEKDEELLRALKEFYDHIAEDILGRTQQLMTSISEYDLSRIYVRNDSQLTDISKKMLGDWNAIYMARERAYDHEQAPKRITAKYERDRIKALKGEESISLANLNSCIAFLDNVRDCRVDTYLSTLGQKEGPHGLSNLVENVFASYHEAEQLLSFPYPEENNLIQDKDNVVLIKNLLDNISDLQRFLKPLWGMGDEPDKDERFYGEYNYIRGALDQVIPLYNKVRNYLTRKPYSTRKVKLNFGNSQLLSGWDRNKEKDNSCVILRKGQNFYLAIMNNRHKRSFENKMLPEYKEGEPYFEKMDYKFLPDPNKMLPKVFLSKKGIEIYKPSPKLLEQYGHGTHKKGDTFSMDDLHELIDFFKHSIEAHEDWKQFGFKFSDTATYENVSSFYREVEDQGYKLSFRKVSESYVYSLIDQGKLYLFQIYNKDFSPCSKGTPNLHTLYWRMLFDERNLADVIYKLDGKAEIFFREKSLKNDHPTHPAGKPIKKKSRQKKGEESLFEYDLVKDRRYTMDKFQFHVPITMNFKCSAGSKVNDMVNAHIREAKDMHVIGIDRGERNLLYICVIDSRGTILDQISLNTINDIDYHDLLESRDKDRQQEHRNWQTIEGIKELKQGYLSQAVHRIAELMVAYKAVVALEDLNMGFKRGRQKVESSVYQQFEKQLIDKLNYLVDKKKRPEDIGGLLRAYQFTAPFKSFKEMGKQNGFLFYIPAWNTSNIDPTTGFVNLFHVQYENVDKAKSFFQKFDSISYNPKKDWFEFAFDYKNFTKKAEGSRSMWILCTHGSRIKNFRNSQKNGQWDSEEFALTEAFKSLFVRYEIDYTADLKTAIVDEKQKDFFVDLLKLFKLTVQMRNSWKEKDLDYLISPVAGADGRFFDTREGNKSLPKDADANGAYNIALKGLWALRQIRQTSEGGKLKLAISNKEWLQFVQERSYEKD* |
| PdCas12a (WP_004356401.1) | MENYQEFTNLFQLNKTLRFELKPIGKTCELLEEGKIFASGSFLEKDKVRADNVSYVKKEIDKKHKIFIEETLSSFSISNDLLKQYFDCYNELKAFKKDCKSDEEEVKKTALRNKCTSIQRAMREAISQAFLKSPQKKLLAIKNLIENVFKADENVQHFSEFTSYFSGFETNRENFYSDEEKSTSIAYRLVHDNLPIFIKNIYIFEKLKEQFDAKTLSEIFENYKLYVAGSSLDEVFSLEYFNNTLTQKGIDNYNAVIGKIVKEDKQEIQGLNEHINLYNQKHKDRRLPFFISLKKQILSDREALSWLPDMFKNDSEVIKALKGFYIEDGFENNVLTPLATLLSSLDKYNLNGIFIRNNEALSSLSQNVYRNFSIDEAIDANAELQTFNNYELIANALRAKIKKETKQGRKSFEKYEEYIDKKVKAIDSLSIQEINELVENYVSEFNSNSGNMPRKVEDYFSLMRKGDFGSNDLIENIKTKLSAAEKLLGTKYQETAKDIFKKDENSKLIKELLDATKQFQHFIKPLLGTGEEADRDLVFYGDFLPLYEKFEELTLLYNKVRNRLTQKPYSKDKIRLCFNKPKLMTGWVDSKTEKSDNGTQYGGYLFRKKNEIGEYDYFLGISSKAQLFRKNEAVIGDYERLDYYQPKANTIYGSAYEGENSYKEDKKRLNKVIIAYIEQIKQTNIKKSIIESISKYPNISDDDKVTPSSLLEKIKKVSIDSYNGILSFKSFQSVNKEVIDNLLKTISPLKNKAEFLDLINKDYQIFTEVQAVIDEICKQKTFIYFPISNVELEKEMGDKDKPLCLFQISNKDLSFAKTFSANLRKKRGAENLHTMLFKALMEGNQDNLDLGSGAIFYRAKSLDGNKPTHPANEAIKCRNVANKDKVSLFTYDIYKNRRYMENKFLFHLSIVQNYKAANDSAQLNSSATEYIRKADDLHIIGIDRGERNLLYYSVIDMKGNIVEQDSLNIIRNNDLETDYHDLLDKREKERKANRQNWEAVEGIKDLKKGYLSQAVHQIAQLMLKYNAIIALEDLGQMFVTRGQKIEKAVYQQFEKSLVDKLSYLVDKKRPYNELGGILKAYQLASSITKNNSDKQNGFLFYVPAWNTSKIDPVTGFTDLLRPKAMTIKEAQDFFGAFDNISYNDKGYFEFETNYDKFKIRMKSAQTRWTICTFGNRIKRKKDKNYWNYEEVELTEEFKKLFKDSNIDYENCNLKEEIQNKDNRKFFDDLIKLLQLTLQMRNSDDKGNDYIISPVANAEGQFFDSRNGDKKLPLDADANGAYNIARKGLWNIRQIKQTKNDKKLNLSISSTEWLDFVREKPYLK* |
| PmCas12a (WP_018359861.1) | MKTQHFFEDFTSLYSLSKTIRFELKPIGKTLENIKKNGLIRRDEQRLDDYEKLKKVIDEYHEDFIANILSSFSFSEEILQSYIQNLSESEARAKIEKTMRDTLAKAFSEDERYKSIFKKELVKKDIPVWCPAYKSLCKKFDNFTTSLVPFHENRKNLYTSNEITASIPYRIVHVNLPKFIQNIEALCELQKKMGADLYLEMMENLRNVWPSFVKTPDDLCNLKTYNHLMVQSSISEYNRFVGGYSTEDGTKHQGINEWINIYRQRNKEMRLPGLVFLHKQILAKVDSSSFISDTLENDDQVFCVLRQFRKLFWNTVSSKEDDAASLKDLFCGLSGYDPEAIYVSDAHLATISKNIFDRWNYISDAIRRKTEVLMPRKKESVERYAEKISKQIKKRQSYSLAELDDLLAHYSEESLPAGFSLLSYFTSLGGQKYLVSDGEVILYEEGSNIWDEVLIAFRDLQVILDKDFTEKKLGKDEEAVSVIKKALDSALRLRKFFDLLSGTGAEIRRDSSFYALYTDRMDKLKGLLKMYDKVRNYLTKKPYSIEKFKLHFDNPSLLSGWDKNKELNNLSVIFRQNGYYYLGIMTPKGKNLFKTLPKLGAEEMFYEKMEYKQIAEPMLMLPKVFFPKKTKPAFAPDQSVVDIYNKKTFKTGQKGFNKKDLYRLIDFYKEALTVHEWKLFNFSFSPTEQYRNIGEFFDEVREQAYKVSMVNVPASYIDEAVENGKLYLFQIYNKDFSPYSKGIPNLHTLYWKALFSEQNQSRVYKLCGGGELFYRKASLHMQDTTVHPKGISIHKKNLNKKGETSLFNYDLVKDKRFTEDKFFFHVPISINYKNKKITNVNQMVRDYIAQNDDLQIIGIDRGERNLLYISRIDTRGNLLEQFSLNVIESDKGDLRTDYQKILGDREQERLRRRQEWKSIESIKDLKDGYMSQVVHKICNMVVEHKAIVVLENLNLSFMKGRKKVEKSVYEKFERMLVDKLNYLVVDKKNLSNEPGGLYAAYQLTNPLFSFEELHRYPQSGILFFVDPWNTSLTDPSTGFVNLLGRINYTNVGDARKFFDRFNAIRYDGKGNILFDLDLSRFDVRVETQRKLWTLTTFGSRIAKSKKSGKWMVERIENLSLCFLELFEQFNIGYRVEKDLKKAILSQDRKEFYVRLIYLFNLMMQIRNSDGEEDYILSPALNEKNLQFDSRLIEAKDLPVDADANGAYNVARKGLMVVQRIKRGDHESIHRIGRAQWLRYVQEGIVE* |
| Lb2Cas12a (WP_044919442.1) | MYYESLTKQYPVSKTIRNELIPIGKTLDNIRQNNILESDVKRKQNYEHVKGILDEYHKQLINEALDNCTLPSLKIAAEIYLKNQKEVSDREDFNKTQDLLRKEVVEKLKAHENFTKIGKKDILDLLEKLPSISEDDYNALESFRNFYTYFTSYNKVRENLYSDKEKSSTVAYRLINENFPKFLDNVKSYRFVKTAGILADGLGEEEQDSLFIVETFNKTLTQDGIDTYNSQVGKINSSINLYNQKNQKANGFRKIPKMKMLYKQILSDREESFIDEFQSDEVLIDNVESYGSVLIESLKSSKVSAFFDALRESKGKNVYVKNDLAKTAMSNIVFENWRTFDDLLNQEYDLANENKKKDDKYFEKRQKELKKNKSYSLEHLCNLSEDSCNLIENYIHQISDDIENIIINNETFLRIVINEHDRSRKLAKNRKAVKAIKDFLDSIKVLERELKLINSSGQELEKDLIVYSAHEELLVELKQVDSLYNMTRNYLTKKPFSTEKVKLNFNRSTLLNGWDRNKETDNLGVLLLKDGKYYLGIMNTSANKAFVNPPVAKTEKVFKKVDYKLLPVPNQMLPKVFFAKSNIDFYNPSSEIYSNYKKGTHKKGNMFSLEDCHNLIDFFKESISKHEDWSKFGFKFSDTASYNDISEFYREVEKQGYKLTYTDIDETYINDLIERNELYLFQIYNKDFSMYSKGKLNLHTLYFMMLFDQRNIDDVVYKLNGEAEVFYRPASISEDELIIHKAGEEIKNKNPNRARTKETSTFSYDIVKDKRYSKDKFTLHIPITMNFGVDEVKRFNDAVNSAIRIDENVNVIGIDRGERNLLYVVVIDSKGNILEQISLNSIINKEYDIETDYHALLDEREGGRDKARKDWNTVENIRDLKAGYLSQVVNVVAKLVLKYNAIICLEDLNFGFKRGRQKVEKQVYQKFEKMLIDKLNYLVIDKSREQTSPKELGGALNALQLTSKFKSFKELGKQSGVIYYVPAYLTSKIDPTTGFANLFYMKCENVEKSKRFFDGFDFIRFNALENVFEFGFDYRSFTQRACGINSKWTVCTNGERIIKYRNPDKNNMFDEKVVVVTDEMKNLFEQYKIPYEDGRNVKDMIISNEEAEFYRRLYRLLQQTLQMRNSTSDGTRDYIISPVKNKREAYFNSELSDGSVPKDADANGAYNIARKGLWVLEQIRQKSEGEKINLAMTNAEWLEYAQTHLL* |

**Supplementary Table 2.** Amino acid sequences of new Cas12a candidates derived from the human metagenome.

|  | Amino acid sequence (N to C terminus) |
| --- | --- |
| mgCas12a-1 | MNNGTNNFQNFIGISSLQKTLRNALIPTETTQQFIVKNGIIKEDELRGENRQILKDIMDDYYRGFISETLSSIDDIDWTSLFEKMEIQLKNGDNKDTLIKEQAEKRKAIYKKFADDDRFKNMFSAKLISDILPEFVIHNNNYSASEKEEKTQVIKLFSRFATSFKDYFKNRANCFSADDISSSSCHRIVNDNAEIFFSNALVYRRIVKNLSNDDINKISGDIKDSLKEMSLEEIYSYEKYGEFITQEGISFYNDICGKVNSFMNLYCQKNKENKNLYKLRKLHKQILCIADTSYEVPYKFESDEEVYQSVNGFLDNISSKHIVERLRKIGDNYNGYNLDKIYIVSKFYESVSQKTYRDWETINTALEIHYNNILPGNGKSKADKVKKAVKNDLQKSITEINELVSNYKLCPDDNIKAETYIHEISHILNNFEAQELKYNPEIHLVESELKASELKNVLDVIMNAFHWCSVFMTEELVDKDNNFYAELEEIYDEIYTVISLYNLVRNYVTQKPYSTKKIKLNFGIPTLADGWSKSKEYSNNAIILMRDNLYYLGIFNAKNKPDKKIIEGNTSENKGDYKKMIYNLLPGPNKMIPKVFLSSKTGVETYKPSAYILEGYKQNKHLKSSKDFDITFCHDLIDYFKNCIAIHPEWKNFGFDFSDTSTYEDISGFYREVELQGYKIDWTYISEKDIDLLQEKGQLYLFQIYNKDFSKKSTGNDNLHTMYLKNLFSEENLKDIVLKLNGEAEIFFRKSSIKNPIIHKKGSILVNRTYEAEEKDQFGNIQIVRKTIPENIYQELYKYFNDKSDKELSDEAAKLKNVVGHHEAATNIVKDYRYTYDKYFLHMPITINFKANKTSFINDRILQYIAKEKNLHVIGIDRGERNLIYVSVIDTCGNIVEQKSFNIVNGYDYQIKLKQQEGARQIARKEWKEIGKIKEIKEGYLSLVIHEISKMVIKYNAIIAMEDLSYGFKKGRFKVERQVYQKFETMLINKLNYLVFKDISITENGGLLKGYQLTYIPDKLKNVGHQCGCIFYVPAAYTSKIDPTTGFVNIFKFKDLTVDAKREFIKKFDSIRYDSEKKLFCFTFDYNNFITQNTVMSKSSWSVYTYGVRIKRRFVNGRFSNESDTIDITKDMEKTLEMTDINWRDGHDLRQDIIDYEIVQHIFEIFRLTVQMRNSLSELEDRDYDRLISPVLNENNIFYDSAKAGDALPKDADANGAYCIALKGLYEIKQITENWKEDGKFSRDKLKISNKDWFDFIQNKRYL* |
| mgCas12a-2 | MGKNQNFQEFIGVSPLQKTLRNELIPTETTKKNITQLDLLTEDEIRAQNREKLKEMMDDYYRNVIDSTLHVGIAVDWSYLFSCMRNHLRENSKESKRELERTQDSIRSQIHNKFAERADFKDMFGASIITKLLPTYIKQNSEYSERYDESMEILKLYGKFTTSLTDYFETRKNIFSKEKISSAVGYRIVEENAEIFLQNQNAYDRICKIAGLDLHGLDNEITAYVDGKTLKEVCSDEGFAKAITQEGIDRYNEAIGAVNQYMNLLCQKNKALKPGQFKMKRLHKQILCKGTTSFDIPKKFENDKQVYDAVNSFTEIVTKNNDLKRLLNITQNANDYDMNKIYVVADAYSMISQFISKKWNLIEECLLDYYSDNLPGKGNAKENKVKKAVKEETYRSVSQLNEVIEKYYVEKTGQSVWKVESYISSLAEMIKLELCHEIDNDEKHNLIEDDEKISEIKELLDMYMDVFHIIKVFRVNEVLNFDETFYSEMDEIYQDMQEIVPLYNHVRNYVTQKPYKQEKYRLYFHTPTLANGWSKSKEYDNNAIILVREDKYYLGILNAKKKPSKEIMAGKEDCSEHAYAKMNYYLLPGANKMLPKVFLSKKGIQDYHPSSYIVEGYNEKKHIKGSKNFDIRFCRDLIDYFKECIKKHPDWNKFNFEFSATETYEDISVFYREVEKQGYRVEWTYINSEDIQKLEEDGQLFLFQIYNKDFAVGSTGKPNLHTLYLKNLFSEENLRDIVLKLNGEAEIFFRKSSVQKPVIHKCGSILVNRTYEITESGTTRVQSIPESEYMELYRYFNSEKQIELSDEAKKYLDKVQCNKAKTDIVKDYRYTMDKFFIHLPITINFKVDKGNNVNAIAQQYIAGRKDLHVIGIDRGERNLIYVSVIDMYGRILEQKSFNLVEQVSSQGTKRYYDYKEKLQNREEERDKARKSWKTIGKIKELKEGYLSSVIHEIAQMVVKYNAIIAMEDLNYGFKRGRFKVERQVYQKFETMLISKLNYLADKSQAVDEPGGILRGYQMTYVPDNIKNVGRQCGIIFYVPAAYTSKIDPTTGFINAFKRDVVSTNDAKENFLMKFDSIQYDIEKGLFKFSFDYKNFATHKLTLAKTKWDVYTNGTRIQNMKVEGHWLSMEVELTTKMKELLDDSHIPYEEGQNILDDLREMKDITTIVNGILEIFWLTVQLRNSRIDNPDYDRIISPVLNKNGEFFDSDEYNSYIDAQKAPLPIDADANGAFCIALKGMYTANQIKENWVEGEKLPADCLKIEHASWLAFMQGERG* |
| CEST01022924.1_4 | MKHIILAAGEGTKSFERSEQIPKCLLKFSSLTTIDRAIAFSKDNNLNEICLVGGFEILKIMKKYPQLRYYFNKNWAKTGNLKSLIIALETLKDDLIITYSDVIYDKSNLKKTKSINNISIQFDSKWVSRYERSGKSSVEILNDKDGQELGEFTGALYIPKALIQFVINLINEIIETDVNGSLFQLAQILIKKSPVKFIDVFGNWAELDSNHDANRLVFGTKSETLDALKNTVRKSKILDQYTFQVGDYEKNKDQIINYIQKTFKDEHLVFRSSALNEDTLHSSMAGSYKSVLKVLRSDKSSIESSIASVVNSYLKSGQKQNLKNQVLVQRYLSGVSSSGVVLTKDLQTDSPYFKINYTDGSDTETVTSGSAGSLKVFILYRGFSEKIKDQKIEKLIDAIRELEIIISYDAIDVEYAFVGGKLYILQVRPIAAKKNYIKVSETDINNEVNSIKQYIESAKDFPGLYGKSIAYGVMPDWNPAEIIGINPKPLAYDLYKYLITDSVWAKSRSFLGYKDVSSNSGLVLFAGRPYVDIRMSFSSFIPKNIQDSVANKLVDGFISKLKSNPEDHDKVEFKTVLTAFDFNFDDKLSELSSYGISESEQKDISNSYRDLTQNIVLENTVKIDEELSYSVILSSRRDEILNSGLSSFDKVYHLLEDCKKYGTLPFANLARMSFIGSILIKSLQTNNVIEPKVVSNFLNSIHSVATDFSNDCNLLLTKHISKEDFLKKYGHLRPGTYEITSQTYNEGYKDYINLNSKKTDQIFDNTYSFSEHDLNKITKKIIHNGFNFSTFTLLKFTKKAMEAREKSKFEFTKNLSAVLDLIIDMGAKYGISKDDLSYLNLEDILRNKKSSSRLDFETNARTSIANNKMKYLISSAIQLPELIFNNRDLEMFHYPESKPNYITFKSVFEEVVFLSKDNNEPLYNKIVMIESADPGFDWIFSHDIKGLITKYGGAASHMAIRCAEFDLPAAIGCGNLFDDLIGCSKVEINCANQIIKGYK* |
| CESD01057036.1_3 | MPNITLGKVKFMHRGTYSASTVYSKGDIVDYDNRMYIFNNDTPKNHAPIFLNTINGTVSSLGIQTNKIRMDFSGFNPQTQKWGLVTSHQRYSGADGSMPGVYSEGGEEFAPPTGLMVYSEYFDPYVGITSINVVDSNTADLYLNQVGINTATISNASFTLGPRRMCGMYEHSVNWLDWDILSEGYNPVGEWNDDTVYLPGDIVQKSNSSYVCGVGHSDVDPKFDYPGCWRIFSRGDDLLDHQSVIGFTNKQPWKWKGHPWIDLPQWGTNNRWNGNIPWNTSLGIGSTSPHAWRWNSGWNDGHMAYRKSNQFLTNNKGNNIVAEGGTPNEYYTQGTSTTHLAVREADVSNAINAKIGVQPDIGNLPRTRTDHDPNIIQAFHGWTSNRANLHSNGTLTMSGVFGNGYLGYGNDHSTDQGINLHKALFKGRSIVKVLTSCHQARDSDSHIMALDEYGEVHLWGRNDTGQVGISSERNRGFGDSSPDILYFSNGSMGVNNRTYLVHSMNKDLFFGGKKIVDIWIGHRWSMCMDEDGELWSWGYNTRGNLGYPTNSGFGDSDRSYSPQKINVNWSSYGGIQKCVIASSEANIDTLYVLDGQGHIWSQGYNGYGQLGSQNTTTGTNSSSITRRTGLQSAGSIVNFWADACNEYGHLWWRNTSGDNYGCGYDGHYNMTGDSSAPSSNTANSPTYMPGSSSNQLLKNCVAMCASGRSGGICQYFLTDKGHVYATGWNGYGEGGTGHSSTLTNNQIRYQQNGQRQYALGRQLHAPFYSAGGSNPVDNTSSSSTVSKGPSNISVDCAGVTCIDIWATGDYDGSSSHTPWSMTLFDNGELTGRGRNYDWGAASNMGNIWGSIHHHGVG* |
| CDYK01036676.1_3 | MNKAADNYTGGNYDEFIALSKVQKTLRNELKPTPFTAEHIKQRGIISEDEYRAQQSLELKKIADEYYRNYITHKLNDINNLDFYNLFDAIEEKYKKNDKDNRDKLDLVEKSKRGEIAKMLSADDNFKSMFEAKLITKLLPDYVERNYTGEDKEKALETLALFKGFTTYFKGYFKTRKNMFSGEGGASSICHRIVNVNASIFYDNLKTFMRIQEKAGDEIALIEEELTEKLDGWRLEHIFSRDYYNEVLAQKGIDYYNQICGDINKHMNLYCQQNKFKANIFKMMKLQKQIMGISEKVFEIPPMYQNDEEVYASFNEFISRLEEVKLTDRLRNILQNINIYNTAKIYINARYYTNVSTYVYGGWGVIESAIERYLCNTIAGKGQSKVKKIENAKKDNKFMSVKELDSIVAEYEPDYFNAPYIDDDDNAVKVFGGQGVLGYFNKMSELLADVSLYTIDYNSDDSLIENKESALRIKKQLDDIMSLYHWLQTFIIDEVVEKDNAFYAELEDICCELENVVTLYDRIRNYVTRKPYSTQKFKLNFASPTLASGWSRSKEFDNNAIILLRNNKYYIAIFNVNNKPDKQIIKGSEEQRLSTDYKKMVYNLLPGPNKMLPKVFIKSDTGKRDYNPSSYILEGYEKNRHIKSSGNFDINYCHDLIDYYKACINKHPEWKNYGFKFKETNQYNDIGQFYKDVEKQGYSISWAYISEEDINKLDEEGKIYLFEIYNKDLSAHSTGRDNLHTMYLKNIFSEDNLKNICIELNGEAELFYRKSSMKSNITHKKDTILVNKTYINETGVRVSLSDEDYMKVYNYYNNNYVIDTENDKNLIDIIEKIGHRKSKIDIVKDKRYTEDKYFLYLPITINYGIEDENVNSKIIEYIAKQDNMNVIGIDRGERNLIYISVIDNKGNIIEQKSFNLVNNYDYKNKLKNMEKTRDNARKNWQEIGKIKDVKSGYLSGVISKIARMVIDYNAIIVMEDLNKGFKRGRFKVERQVYQKFENMLISKLNYLVFKERKADENGGILRGYQLTYIPKSIKNVGKQCGCIFYVPAAYTSKIDPATGFINIFDFKKYSGSGINAKVKDKKEFLMSMNSIRYINEGSEEYEKIGHRELFAFSFDYNNFKTYNVSSPVNEWTAYTYGERIKKLYKDGRWLRSEVLNLTENLIKLMEQYNIEYKDGHDIREDISHMDETRNADFICSLFEELKYTVQLRNSKSEAEDENYDRLVSPILNSSNGFYDSSDYMENENNTTHTMPKDADANGAYCIALKGLYEINKIKQNWSDDKKFKENELYINVTEWLDYIQNRRFE* |
| CDYL01005663.1_6 | MNGNRSIVYREFVGVTPVAKTLRNELRPVGHTQEHIIQNGLIQEDELRQEKSTELKNIMDDYYREYIDKSLSGLTDLDFTLLFELMNSVQSSLSKDNKKALEKEQSKMREQICTHLQSDSNYKNIFNAKLLKEILPDFIKNYNQYDAKDKAGKLETLALFNGFSTYFTDFFEKRKNVFTKEAVSTSIAYRIVHENSLTFLANMTSYKKISEKALDEIEVIEKNNQDKMGDWELNQIFNPDFYNMVLIQSGIDFYNEICGVVNAHMNLYCQQTKNNYNLFKMRKLHKQILAYTSTSFEVPKMFEDDMSVYNAVNAFIDETEKGNIIGKLKDIVNKYDELDEKRIYISKDFYETLSCFMSGNWNLITGCVENFYDENIHAKGKSKEEKVKKAVKEDKYKSINDVNDLVEKYIDEKERNEFKNSNAKQYIREISNIITDTETAHLEYNEHISLIESEEKADEIKKRLDMYMNMYHWIKAFIVDEVLDRDEMFYSDIDDIYNILENIVPLYNRVRNYVTQKPYNSKKIKLNFQSPTLDNGWSQSKEFDNNAIILIRDNKYYLAIFNAKNKPDKKIIQGNSDKKNDNDYKKMVYNLLPGANKMLPKVFLSKKGIETFKPSDYIISGYNAHKHIKTSENFDISFCRDLIDYFKNSIEKHAEWRKYEFKFSATDSYNDISEFYREVEMQGYRIDWTYISEADINKLDEEGKIYLFQIYNKDFAENSTGKENLHTMYFKNIFSEENLKDIIIKLNGQAELFYRRASVKNPVKHKKDSVLVNKTYKNQLDNGDVVRIPIPDDIYNEIYKMYNGYIKENDLSEAAKEYLDKVEVRTAQKDIVKDYRYTVDKYFIHTPITINYKVTARNNVNDMAVKYIAQNDDIHVIGIDRGERNLIYISVIDSHGNIVKQKSYNILNNYDYKKKLVEKEKTREYARKNWKSIGNIKELKEGYISGVVHEIAMLMVEYNAIIAMEDLNYGFKRGRFKVERQVYQKFESMLINKLNYFASKGKSVDEPGGLLKGYQLTYVPDNIKNLGKQCGVIFYVPAAFTSKIDPSTGFISAFNFKSISTNASRKQFFMQFDEIRYCAEKDMFSFGFDYNNFDTYNITMGKTQWTVYTNGERLQSEFNNARRTGKTKSINLTETIKLLLEDNEINYADGHDVRIDMEKMDEDKNSEFFAQLLSLYKLTVQMRNSYTEAEEQEKGISYDKIISPVINDEGEFFDSDNYKESDDKECKMPKDADANGAYCIALKGLYEVLKIKSEWTEDGFDRNCLKLPHAEWLDFIQNKRYE* |
| CDYK01004246.1_121 | MNGNRSIVYREFVGVIPVAKTLRNELRPVGHTQEHIIQNGLIQEDELRQEKSTELKNIMDDYYREYIDKSLSGVTDLDFTLLFELMNLVQSSPSKDNKKALEKEQSKMREQICTHLQSDSNYKNIFNAKLLKEILPNFIKNYNQYDVKDKACKLETLALFNGFSTYFTDFFEKRKNVFTKEAVSTSIAYRIVHENSLIFLANMTSYKKISEKALDEIEVIEKNNQDKMGDWELNQIFNPDFYNMVLIQSGIDFYNEICGVVNAHMNLYCQQTKNNYNLFKMRKLHKQILAYTSTSFEVPKMFEDDMSVYNAVNAFIDETEKGNIIGKLKDIANKYDELDEKRIYISKDFYETLSCFMSGNWNLITGCVENFYDENIHAKGKSEEEKVKKAVKEDKYKSINDVNDLVEKYIDEKERNEFKNSNAKQYIREISNIITDTETAHLEYDEHISLIESEEKADEMKKRLDMYMNMYHWAKAFIVDEVLDRDEMFYSDIDDIYNILENIVPLYNRVRNYVTQKPYNSKKIKLNFQSPTLANGWSQSKEFDNNAIILIRDNKYYLAIFNAKNKPDKKIIQGNSDKKNDNDYKKMVYNLLPGANKMLPKVFLSKKGIETFKPSDYIISGYNAHKHIKTSENFDISFCRDLIDYFKNSIEKHAEWRKYGFKFSATDSYNDISEFYREVEMQGYRIDWAYIGEADINKLDEEGKIYLFQIYNKDFAENSTGKENLHTMYFKNIFSEENLKNIVIKLNGQAELFYRKASVKNPVKHKKDSVLVNKTYKNQLDNGDVVRIPIPDDIYNEIYKMYNGYIKESDLSGAAKEYLDKVEVRTAQKEIVKDYRYTVDKYFIHTPITINYKVAARNNVNDMAVKYIAQNDDIHVIGIDRGERNLIYISVIDSHGNIVKQKSYNILNNYDYKKKLVEKEKTREYARKNWKSIGNIKELKEGYISGVVHEIAMLMVEYNAIIAMEDLNYGFKRGRFKVERQVYQKFESMLINKLNYFASKGKSVDEPGGLLKGYQLTYVPDNIKNLGKQCGVIFYVPAAFTSKIDPSTGFISAFNFKSISTNDSRKQFFMQFDEIRYCAEKDMFSFGFDYNNFDTYNITMGKTQWTVYTNGERLQSEFNNARRTGKTKSINLTETIKLLLEDNEINYADGHDVRIDMEKMDEDKNSEFFAQLLSLYKLTVQMRNSYTEAEEQEKGISYDKIISPVINDEGEFFDSDNYKESDDKECKMPKDADANGAYCIALKGLYEVLKIKSEWTEDGFDRNCLKLPHAEWLDFIQNKRYE* |
| CDYL01025564.1_3 | MNKAADNYTGGNYDEFIALSKVQKTLRNELKPTPFTAEHIKQRGIISEDEYRAQQSLELKKIADEYYRNYITHKLNGINNLDFYNLFDAIEEKYKKNDKENRDKLDLVEKSKRGEIAKLLSADDNFKSMFEAKLITKLLPDYVEQNYIGEDKEKALETIALFKGFTTYFTDYFNIRKNMFKENGGASSICYRIVNVNASIFYDNLKTFMCIKEKAETEIALIEEELTELLDSWRLEHIFSEDYYNELLAQKGIDYYNQICGDVNKHMNLYCQQNKLKANVFKMTKLQKQIMGISEKAFEIPPMYQNDEEVYASFNEFISRLEEVKLTDRLRNILQNINIYNTAKIYINARYYTNVSTYVYGGWGVIDSAIERYLYNTIAGKGQSKVKKIENAKKDNKFMSVKELDSIVAEYEPDYFNAPYIDDDDNAVKAFGGQGVLGYFNKMSELLADVSLYTIDYNSDDSLIENKESALRIKKQLDDIMSLYHWLQTFIIDEVVEKDNAFYAELEDICCELENVVTLYDRIRNYVTKKPYSTQKFKLNFASPTLASGWSRSKEFDNNAIILLRNNKYYIAIFNVNNKPDKQIIKGSEEQRLSTDYKKMVYNLLPGPNKMLPKVFIKSDTGKRDYNPSSYILEGYGKNRHIKSSGNFDINYCHDLIDYFKACINKHPEWKNYGFKFEETTQYNDIGQFYKDVEKQGYSISWVYISEADINRLDEEGKIYLFEIYNKDLSSHSTGKDNLHTMYLKNIFSEDNLKNICIELNGNAELFYRKSSMKRNITHKKDTVLVNKTYINEAGVRVSLTDEDYIKVYNYYNNDYVIDVEKDKKLVEILERIGHRKNPIDIIKDKRYTEDKYFLHLSITINYGVDDENINAKMIEYIAKHNNMNVIGIDRGERNLIYISVINNKGNIIEQKSFNLVNSYDYKNKLKNMEKTRDNARKNWQEIGKIKDVKSGYLSGVISKIARMVIDYNAIIVMEDLNKGFKRGRFKVERQVYQKFENMLISKLNYLVFKERKADENGGILRGYQLTYIPKSIKNVGKQCGCIFYVPAAYTSKIDPSTGFINIFDFKKYSGSGINAKVKDKKEFLMSMNSIRYINEGSEEYEKIGHRELFAFSFDYNNFKTYNVSSPVNEWTAYTYGERIKKLYKDGRWLRSEVLNLTENLIKLMEQYNIEYKDGHDIREDISHMDETRNADFICSLFEELKYTVQLRNSKSEAEDENYDRLVSPILNSSNGFYDSSDYMENENNTTHTMPKDADANGAYCIALKGLYEINKIKQNWSDDKKFKENELYINVVEWLDYIQNRRFE* |
| CEAM01003869.1_48 | MNKAADNYTGGNYDEFIALSKVQKTLRNELKPTPFTAEHIKQRGIISEDEYRSQQSLELKKIADEYYRNYITHKLNDINNLDFYNLFDAIEEKYKKNDKENRDKLDLVEKSKRGEIAKLLSADDNFKSMFEAKLITKLLPDYVEQNYIGEDKEKALETIALFKGFTTYFTDYFNIRKNMFKENGGASSICYRIVNVNASIFYDNLKTFMCIKEKAETEIALIEEELTELLDSWRLEHIFSEDYYNELLAQKGIDYYNQICGDVNKHMNLYCQQNKLKANVFKMTKLQKQIMGISEKVFEIPPMYQNDEEVYASFNEFISRLEEVKLTDRLRNILQNINIYNTAKIYINARYYTNVSSYVYGGWGVIDSAIERYLYNTIAGKGQSKVKKIENAKKDNKFMSVKELDSIVAEYEPDYFNAPYIDDDDNAVKAFGGQGVLGYFNKMSELLADVSLYTIDYNSDDSLIENKESALRIKKQLDDIMSLYHWLQTFIIDEVVEKDNAFYAELEDICCELENVVTLYDRIRNYVTKKPYSTQKFKLNFASPTLAAGWSRSKEFDNNAIILLRNNKYYIAIFNVNNKPDKQIIKGSEEQRLSTDYKKMVYNLLPGPNKMLPKVFIKSDTGKRDYNPSSYILEGYEKNRHIKSSGNFDINYCHDLIDYYKACINKHPEWKNYGFKFKETNQYNDIGQFYKDVEKQGYSISWAYISEEDINKLDEEGKIYLFEIYNKDLSAHSTGRDNLHTMYLKNIFSEDNLKNICIELNGEAELFYRKSSMKSNITHKKDTILVNKTYINETGVRVSLSDEDYMKVYNYYNNNYVIDTENDKNLIDIIEKIGHRKSKIDIVKDKRYTEDKYFLYLPITINYGIEDENVNSKIIEYIAKQDNMNVIGIDRGERNLIYISVIDNKGNIIEQKSFNLVNNYDYKNKLKNMEKTRDNARKNWQEIGKIKDVKSGYLSGVISEIARMVIDYNAIIVMEDLNKGFKRGRFKVERQVYQKFENMLISKLNYLVFKERKADENGGILRGYQLTYIPKSIKNVGKQCGCIFYVPAAYTSKIDPATGFINIFDFKKYSGSGINAKVKDKKEFLMSMNSIRYINEGSEEYEKIGHRELFAFSFDYNNFKTYNVSSPVNEWTAYTYGERIKKLYKDGRWLRSEVLNLTENLIKLMEQYNIEYKDGHDIREDISHMDETRNADFICSLFEELKYTVQLRNSKSEAEDENYDRLVSPILNSSNGFYDSSDYMENENNTTHTMPKDADANGAYCIALKGLYEINKIKQNWSDDRKFKENELYINVTEWLDYIQNRRFE* |
| CDZY01023362.1_31 | MNKAADNYTGGNYDEFIALSKVQKTLRNELKPTPFTAEHIKQRGIISEDEYRSQQSLELKKIADEYYRNYITHKLNDINNLDFYNLFDAIEEKYKKNDKENRDKLDLVEKSKRGEIAKLLSADDNFKSMFEAKLITKLLPDYVEQNYIGEDKEKALETIALFKGFTTYFTDYFNIRKNMFKENGGASSICYRIVNVNASIFYDNLKTFMCIKEKAETEIALIEEELTELLDSWRLEHIFSEDYYNELLAQKGIDYYNQICGDVNKHMNLYCQQNKLKANVFKMTKLQKQIMGISEKVFEIPPMYQNDEEVYASFNEFISRLEEVKLTDRLRNILQNINIYNTAKIYINARYYTNVSSYVYGGWGVIDSAIERYLYNTIAGKGQSKVKKIENAKKDNKFMSVKELDSIVAEYEPDYFNAPYIDDDDNAVKAFGGQGVLGYFNKMSELLADVSLYTIDYNSDDSLIENKESALRIKKQLDDIMSLYHWLQTFIIDEVVEKDNAFYAELEDICCELENVVTLYDRIRNYVTKKPYSTQKFKLNFASPTLAAGWSRSKEFDNNAIILLRNNKYYIAIFNVNNKPDKQIIKGSEEQRLSTDYKKMVYNLLPGPNKMLPKVFIKSDTGKRDYNPSSYILEGYEKNRHIKSSGNFDINYCHDLIDYYKACINKHPEWKNYGFKFKETNQYNDIGQFYKDVEKQGYSISWAYISEEDINKLDEEGKIYLFEIYNKDLSAHSTGRDNLHTMYLKNIFSEDNLKNICIELNGEAELFYRKSSMKSNITHKKDTILVNKTYINETGVRVSLSDEDYMKVYNYYNNNYVIDTENDKNLIDIIEKIGHRKSKIDIVKDKRYTEDKYFLYLPITINYGIEDENVNSKIIEYIAKQDNMNVIGIDRGERNLIYISVIDNKGNIIEQKSFNLVNNYDYKNKLKNMEKTRDNARKNWQEIGKIKDVKSGYLSGVISEIARMVIDYNAIIVMEDLNKGFKRGRFKVERQVYQKFENMLISKLNYLVFKERKADENGGILRGYQLTYIPKSIKNVGKQCGCIFYVPAAYTSKIDPATGFINIFDFKKYSGSGINAKVKDKKEFLMSMNSIRYINEGSEEYEKIGHRELFAFSFDYNNFKTYNVSSPVNEWTAYTYGERIKKLYKDGRWLRSEVLNLTENLIKLMEQYNIEYKDGHDIREDISHMDETRNADFICSLFEELKYTVQLRNSKSEAEDENYDRLVSPILNSSNGFYDSSDYMENENNTTHTMPKDADANGAYCIALKGLYEINKIKQNWSDDRKFKENELYINVTEWLDYIQNRRFE* |
| CDYR01026036.1_2 | MNNGTNNFQNFIGISSLQKTLRNALIPTETTQQFIVKNGIIKEDELRGENRQILKDIMDDYYRGFISETLSSIDDIDWTSLFEKMEIQLKNGDNKDTLIKEQAEKRKAIYKKFADDDRFKNMFSAKLISDILPEFVIHNNNYSASEKEEKTQVIKLFSRFATSFKDYFKNRANCFSADDISSSSCHRIVNDNAEIFFSNALVYRRIVKNLSNDDINKISGDIKDSLKEMSLEEIYSYEKYGEFITQEGISFYNDICGKVNSFMNLYCQKNKENKNLYKLRKLHKQILCIADTSYEVPYKFESDEEVYQSVNGFLDNISSKHIVERLRKIGDNYNGYNLDKIYIVSKFYESVSQKTYRDWETINTALEIHYNNILPGNGKSKADKVKKAVKNDLQKSITEINELVSNYKLCPDDNIKAETYIHEISHILNNFEAQELKYNPEIHLVESELKASELKNVLDVIMNAFHWCSVFMTEELVDKDNNFYAELEEIYDEIYTVISLYNLVRNYVTQKPYSTKKIKLNFGIPTLADGWSKSKEYSNNAIILMRDNLYYLGIFNAKNKPDKKIIEGNTSENKGDYKKMIYNLLPGPNKMIPKVFLSSKTGVETYKPSAYILEGYKQNKHLKSSKDFDITFCHDLIDYFKNCIAIHPEWKNFGFDFSDTSTYEDISGFYREVELQGYKIDWTYISEKDIDLLQEKGQLYLFQIYNKDFSKKSTGNDNLHTMYLKNLFSEENLKDIVLKLNGEAEIFFRKSSIKNPIIHKKGSILVNRTYEAEEKDQFGNIQIVRKTIPENIYQELYKYFNDKSDKELSDEAAKLKNVVGHHEAATNIVKDYRYTYDKYFLHMPITINFKANKTSFINDRILQYIAKEKNLHVIGIDRGERNLIYVSVIDTCGNIVEQKSFNIVNGYDYQIKLKQQEGARQIARKEWKEIGKIKEIKEGYLSLVIHEISKMVIKYNAIIAMEDLSYGFKKGRFKVERQVYQKFETMLINKLNYLVFKDISITENGGLLKGYQLTYIPDKLKNVGHQCGCIFYVPAAYTSKIDPTTGFVNIFKFKDLTVDAKREFIKKFDSIRYDSEKKLFCFTFDYNNFITQNTVMSKSSWSVYTYGVRIKRRFVNGRFSNESDTIDITKDMEKTLEMTDINWRDGHDLRQDIIDYEIVQHIFEIFRLTVQMRNSLSELEDRDYDRLISPVLNENNIFYDSAKAGDALPKDADANGAYCIALKGLYEIKQITENWKEDGKFSRDKLKISNKDWFDFIQNKRYL* |

**Supplementary Table 3.** Percentage identity of amino acid sequences from eight novel Cas12a candidates and three previously known Cas12a proteins. All protein sequences were aligned, and the percentage identity was calculated by Clustal2.1. Candidates with 90% sequence identity are highlighted in pink. Candidates without direct repeat sequences were excluded from the analysis.

| Amino acid length |  | AsCas12a | CDZY01023362.1_31 | CEAM01003869.1_48 | CDYK01036676.1_3 | FnCas12a | LbCas12a | CDYL01005663.1_6 | CDYK01004246.1_121 | mgCas12a-2 | CDYR01026036.1_2 | mgCas12a-1 |
| --- | --- | --- | --- | --- | --- | --- | --- | --- | --- | --- | --- | --- |
| 1,365 | AsCas12a | 100 |  |  |  |  |  |  |  |  |  |  |
| 1,305 | CDZY01023362.1_31 | 33.33 | 100 |  |  |  |  |  |  |  |  |  |
|  | CEAM01003869.1_48 | 33.33 | 100 | 100 |  |  |  |  |  |  |  |  |
|  | CDYK01036676.1_3 | 33.42 | 97.39 | 97.39 | 100 |  |  |  |  |  |  |  |
| 1,301 | FnCas12a | 38.12 | 38.46 | 38.46 | 38.29 | 100 |  |  |  |  |  |  |
| 1,286 | LbCas12a | 36.03 | 36.73 | 36.73 | 36.98 | 42.40 | 100 |  |  |  |  |  |
| 1,282 | CDYL01005663.1_6 | 33.91 | 51.18 | 51.18 | 51.26 | 40.12 | 38.47 | 100 |  |  |  |  |
|  | CDYK01004246.1_121 | 34.32 | 51.18 | 51.18 | 51.26 | 40.37 | 38.13 | 98.05 | 100 |  |  |  |
| 1,275 | mgCas12a-2 | 33.33 | 46.30 | 46.30 | 46.46 | 37.75 | 36.78 | 51.75 | 51.75 | 100 |  |  |
| 1,263 | CDYR01026036.1_2 | 34.90 | 51.84 | 51.84 | 52.08 | 39.39 | 37.42 | 52.95 | 52.95 | 53.45 | 100 |  |
|  | mgCas12a-1 | 34.90 | 51.84 | 51.84 | 52.08 | 39.39 | 37.42 | 52.95 | 52.95 | 53.45 | 100 | 100 |

**Supplementary Table 4**. Repeat and spacer sequences identified in CRISPR arrays of *mgCas12a*. Mismatched repeat sequences are in red.

|  |  | Repeat sequence (5’-3’) |  | Spacer sequence (5’-3) |
| --- | --- | --- | --- | --- |
| mgCas12a-1 | #1 | GTTAAGTAATATAGAATAATTTCTACTGTTGTAGAT |  | TCACCTCCCTGTAATAAAAAGAGCT |
|  | #2 | GTTAAGTAATATAGAATGATTTCTACTGTTGTAGAT |  | TTCGCCAAACCTTCTATTTAGCAAATAG |
|  | #3 | GTTAAATAATATAGAATAATTTCTACTGTTGTAGAT |  | TTCTACTCTTTGACTAAGGTAAACAAG |
|  | #4 | GTTAAGTAATGTAGAATAATTTCTACTGTTGTAGAT |  | ATTGGTATATATCCTACAATTTTAATC |
|  | #5 | GTTAAGTAATATAGAATAATTTCTACTGTTGTAGAT |  | GGTTGTGCCGTCAGAAAAGCTTTTTGAAA |
|  | #6 | GTTAAGTAATATAGAATAATTTCTACTGTTGTAGAT |  | TATGTCTAAAACTGTCGCTATCTATGTC |
|  | #7 | GTTAAGTAATATAGAATAATTTCTACTGTTGTAGAT |  |  |
| mgCas12a-2 | #1 | GTTGAATAACCTTAAATAATTTCTACTGTTGTAGAT |  | CTATGACACCCTCAAAAGAGTTCATA |
|  | #2 | GTTGAATAACCTTAAATAATTTCTACTGTTGTAGAT |  | AAAGTGTTACCCGTGTTACCTTTTCGGCA |
|  | #3 | GTTGAATAACCTTAAATAATTTCTACTGTTGTAGAT |  | TATAGTGCAAGAAGATATGAATTATCTG |
|  | #4 | GTTGAATAACCTTAAATAATTTCTACTGTTGTAGAT |  | CGCATAATATAATCTTCCGAACATTTT |
|  | #5 | GTTGAATAACCTTAAATAATTTCTACTGTTGTAGAT |  | ATATCGCACTGATTCATGACAGCGGC |
|  | #6 | GTTGAATAACCTTAAATAATTTCTACTGTTGTAGAT |  | TACGGCAGAGCCGGATGTTAGTAAATT |
|  | #7 | GTTGAATAACATTAAATAATTTCTACTATGTAGATA |  |  |

**Supplementary Table 5.** List of PCR amplicons used for in-tube assays. Bold nucleotides indicate Cas12a RNP binding sites; underlined nucleotides indicate the PAM.

|  | Sequence (5’-3’) |
| --- | --- |
| Target DNA #1 (*HsEMX1*) | GTAAAACGACGGCCAGTGAATTGTAATACGATTCACTATAGGGCGAATTGGGCCCTCTAGATGCATGCTCGAGCGGCCGCCAGTGTGATGGATATCTGCAGAATTCGCCCTTGTGGGGACAGAAGGTCTGGAGCTGCCCGTGAAGGGCAGAATGCTGCCCTCAGACCCGCTTCCTCCCTGTCCTTGTCTGTCCAAGGAGAATGAGGTCTCACTGGTGGATTTCGGACTACCCTGAGGAGCTGGCACCTGAGGGACAAGGCCCCCCACCTGCCCAGCTCCAGCCTCTGATGAGGGGTGGGAGAGAGCTACATGAGGTTGCTAAGAAAGCCTCCCCTGAAGGAGACCACACAGTGTGTGAGGTTGGAGTCTCTAGCAGCGGGTTCTGTGCCCCCAGGGATAGTCTGGCTGTCCAGGCACTGCTCTTGATATAAACACCACCTCCTAGTTATGAAACCATGCCCATTCTGCCTCTCTGTATGGAAAAGAGCATGGGGCTGGCCCGTGGGGTGGTGTCCACTTTAGGCCCTGTGGGAGATCATGGGAACCCACGCAGTGGGTCATAGGCTCTCTCATTTACTACTCACATCCACTCTGTGAAGAAGCGATTATGATCTCTCCTCTAGAAACTCGTAGAGTCCCATGTCTGCCGGCTTCCAGAGCCTGCACTCCTCCACCTTGGCTTGGCTTTGCTGGGGCTAGAGGAGCTAGGATGCACAGCAGCTCTGTGACCCTTTGTTTGAGAGGAACAGGAAAACCACCCTTCTCTCTGGCCCACTGTGTCCTCTTCCTGCCCTGCCATCCCCTTCTGTGAATGTTAGACCCATGGGAGCAGCTGGTCAGAGGGGACCCCGGCCTGGGGCCCCTAACCCTATGTAGCCTCAGTCTTCCCATCAGGCTCTCAGCTCAGCCTGAGTGTTGAGGCCCCAGTGGCTGCTCTGGGGGCCTCCTGAGTTTC**TCATCTGTGCCCCTCCCTCCCTG**GCCCAGGTGAAGGTGTGGTTCCAGAACCGGAGGACAAAGTACAAACGGCAGAAGCTGGAGGAGGAAGGGCCTGAGTCCGAGCAGAAGAAGAAGGGCTCCCATCACATCAACCGGTGGCGCATTGCCACGAAGCAGGCCAATGGGGAGGACATCGATGTCACCTCCAATGACTAGGGTGGGCAACCACAAACCCACGAGGGCAGAGTGCTGCTTGCTGCTGGCCAGGCCCCTGCGTGGGCCCAAGCTGGACTCTGGCCACTCCCTGGCCAGGCTTTGGGGAGGCCTGGAGTCATGGCCCCACAGGGCTTGAAGCCCGGGGCCGCCATTGACAGAGGGACAAGCAATGGGCTGGCTGAGGCCTGGGACCACTTGGCCTTCTCCTCGGAGAGCCTGCCTGCCTGGGCGGGCCCGCCCGCCACCGCAGCCTCCCAGCTGCTCTCCGTGTCTCCAATCTCCCTTTTGTTTTGATGCATTTCTGTTTTAATTTATTTTCCAGGCACCACTGTAGTTTAGTGATCCCCAGTGTCCCCCTTCCCTATGGGAATAATAAAAGTCTCTCTCTTAATGACACGGGCATCCAGCTCCAGCCCCAGAAAGGGCGAATTCCAGCACACTGGCGGCCGTTACTAGTGGATCCGAGCTCGGTACCAAGCTTGGCGTAATCATGGTCATAGCTGTTTCCTG |
| Target DNA #2 (*HsDNMT1*) | GTAAAACGACGGCCAGTGAATTGTAATACGATTCACTATAGGGCGAATTGGGCCCTCTAGATGCATGCTCGAGCGGCCGCCAGTGTGATGGATATCTGCAGAATTCGCCCTTGCTGCTCTCGAACTCCTGGCCTCAACTAATCCACCTGCCTTGGCCTCCCAAAGTGCTGGGATTACAGGCGTGAGCCACTGCTCCCAGCCCCACGTGTCTTTGTCTCAAGTCTTTCTGAAGCTCTTCAAAGGCCCAGTGACTTGTGGCTGTGGGGCGGGATGATGGGCCAGTTGGAGGGTCCAAGGATCTTGTGCTGGAAGGGTTTTGGGCCCATGTGAGCAGGACCAGAACCCTTCCCCAAGGGGTGCAATGCCCAGGTTGTCCTCCATCTGAGCAGGGGCTGGCAGTACACCTGCCCCCGGGCCTTGGGCCTGGGTGTCCACATCAGGCATTGCCCTTCTCCCCTCCTGCAGGTGGGCAATGCCGTGCCACCGCCCCTGGCCAAAGCCATTGGCTTGGAGATCAAGCTTTGTATGTTGGCCAAAGCCCGAGAGAGTGCCTCAGGTATGGTGGGGTGGGCCAGGCTTCCTCTGGGGCCTGACTGCCCTCTGGGGGTACATGTGGGGGCAGTTGCTGGCCACCGTTTTGGGCTCTGGGACTCAGGCGGGTCACCTACCCACGTTCGTGGCCCCATCTTTCTCAAGGGGCTGCTGTGAGGATTGAGTGAGTTGCACGTGTCAAGTGCTTAGAGCAGGCGTGCTGCACACAGCAGGCCTTTGGTCAGGTTGGCTGCTGGGCTGGCCCTGGGGCCGTTTCCCTCACTCCTGCTCGGTGAATTTGGCTCAGCAGGCACCTGCCTCAGCTGCTCACTTGAGCCTCTGGGTCTAGAACCCTCTGGGGACCGTTTGAGGAGTGTTCAGTCTCCGTGAACGTTCCCTTAGCACTCTGCCACTTATTGGGTCAGCTGTTAACATCAGTACGTTAATGTTTCCTGATGGTCCATGTCTGTTACTCGCCTGTCAAGTGGCGTGACACCGGGCGTGTTCCCCAGAGTGACTTTTCCTTTTATTTCCCTTCAGCTAAAATAAAGGAGGAGGAAGCTGCTAAGGACTAGTTCTGCCCTCCCGTCACCCCTGTTTC**TGGCACCAGGAATCCCCAACATG**CACTGATGTTGTGTTTTTAACATGTCAATCTGTCCGTTCACATGTGTGGTACATGGTGTTTGTGGCCTTGGCTGACAAGGGCGAATTCCAGCACACTGGCGGCCGTTACTAGTGGATCCGAGCTCGGTACCAAGCTTGGCGTAATCATGGTCATAGCTGTTTCCTG |
| Target DNA #3 (*HsCCR5*) | CAGGAAACAGCTATGACCATGATTACGCCAAGCTTGGTACCGAGCTCGGATCCACTAGTAACGGCCGCCAGTGTGCTGGAATTCGCCCTTCAGAGGCCATCCTCACCCTGACCTGAGGGCTGTTGGTGGTCTCCTTGCCCTAAATGCTCATCCTTCCTGACCCTCCTTTGGCCACAGAGTAAACCTTCTGCAACACCAACCAGGATCTCCCTGCTCAGCTCATGACTTAGACGGAGCAATGCCGTCAAGGTTCTTCATGATCTAGCCTTGTCCTTCCTCCCCATGTCTTCCCAACCAGCTCTGTCTCCTTCTACAGCCAAGCTTGCCCATGCAGTGCTTGCAGTGAGGCTTCTGTCTTTGCCAGCAATAGATGATCCAACTCAAATTCCTTCTCATTTAAGAAGCTTCCCCAGCTCTCCCAGGCCACAAGTCTCTCGCCTGGTTCTAAGTCAGTGAAACTTATTAACCATACCTTGGAGGGGAAATCACACATGAAAAGTGTCATTTCTTTACTAATCATATTCATGTCTTTTCTCCCCATAGCAAGACAAAGACCTGTTTTAAACACATTTACAACCTATATGTTGCCTTGTACTAGGTAAAAAGTTGTACATTTCTGAAATAATTTTGGTATTTCTGTTCAGATCACTAAACTCAAGAATCAGCAATTCTCTGAGGCTTTCTTTTAAATATACATAAGGAACTTTCGGAGTGAAGGGAGAGTTTGTCAATAACTTGATGCATGTGAAGGGGAGATAAAAAGGTTGCTATTTTTCATCAACATATTTTGATTTGGCTTTCTATAATTGATGGGCTTAAAAGATCTAATCTACTTTAAACAGATGCCAAATAAATGGATGAATCTTAGACCCTCTATAACAGTAACTTCCTTTTAAAAAAGACCTCTCCCACCCCACCCCCAGCCCAGGCTGTGTATGAAAACTAAGCCATGTGCACAACTCTGACTGGGTCACCAGCCCACTTGAGTCCGTGTCACAAGCCCACAGATATTTC**CTGCTCCCCAGTGGATCGGGTGT**AAACTGAGCTTGCTCGCTCGGGAGCCTCTTGCTGGAAAATAGAACAGCATTTGCAGAAGCGTTTGGCAATGTGCTTTTGGAAGAAGACTAAGAGGTAGTTTCTGAACTTCTCCCCGACAAAGGCATAGATGATGGGGTTGATGCAGCAGTGCGTCATCCCAAGAGTCTCTGTCACCTGCATAGCTTGGTCCAACCTGTTAGAGCTACTGCAATTATTCAGGCCAAAGAATTCCTGGAAGGTGTTCAGGAGAAGGACAATGTTGTAGGGAGCCCAGAAGAGAAAATAAACAATCATGATGGTGAAGATAAGCCTCACAGCCCTGTGCCTCTTCTTCTCATTTCGACACCGAAGCAGAGTTTTTAGGATTCCCGAGTAGCAGATGACCATGACAAGCAGCGGCAGGACCAGCCCCAAGATGACTATCTTTAATGTCTGGAAATTCTTCCAGAATTGATACTGACTGTATGGAAAATGAGAGCTGCAGGTGTAATGAAGACCTTCTTTTTGAGATCTGGTAAAGATGATTCCTGGGAGAGACGCAAACACAGCCACCACCAAGGGCGAATTCTGCAGATATCCATCACACTGGCGGCCGCTCGAGCATGCATCTAGAGGGCCCAATTCGCCCTATAGTGAATCGTATTACAATTCACTGGCCGTCGTTTTAC |

**Supplementary Table 6.** Sequences of the crRNAs used in this study. Guide sequences are underlined. Sequences of AsCas12a 5′ handle are in blue, the FnCas12a 5′ handle is in orange, and the LbCas12a 5′ handle is in purple.

| Target DNA | Sequence (5’-3’) | Length (nt) |
| --- | --- | --- |
| #1 (*HsEMX1*) | UAAUUUCUACUCUUGUAGAUCUGCUCCCCAGUGGAUCGGGUGU | 43 |
|  | UAAUUUCUACUGUUGUAGAUCUGCUCCCCAGUGGAUCGGGUGU | 43 |
|  | UAAUUUCUACUAAGUGUAGAUCUGCUCCCCAGUGGAUCGGGUGU | 44 |
| #2 (*HsDNMT1*) | UAAUUUCUACUCUUGUAGAUUGGCACCAGGAAUCCCCAACAUG | 43 |
|  | UAAUUUCUACUGUUGUAGAUUGGCACCAGGAAUCCCCAACAUG | 43 |
|  | UAAUUUCUACUAAGUGUAGAUUGGCACCAGGAAUCCCCAACAUG | 44 |
| #3 (*HsCCR5*) | UAAUUUCUACUCUUGUAGAUUCAUCUGUGCCCCUCCCUCCCUG | 43 |
|  | UAAUUUCUACUGUUGUAGAUUCAUCUGUGCCCCUCCCUCCCUG | 43 |
|  | UAAUUUCUACUAAGUGUAGAUUCAUCUGUGCCCCUCCCUCCCUG | 44 |

**Supplementary Table 7.** List of oligonucleotides used in this study.

| Name | Sequence (5’-3’) | Note |
| --- | --- | --- |
| AsCas12a F1 | ACTGGTGGACAGCAAATGGGTCGCATGACCCAGTTCGAGGGGTT | Gibson Assembly |
| AsCas12a R1 | ACCCTTTCTGATCCCCGGTT |  |
| AsCas12a F2 | AAAAAAACCGGGGATCAGAAAGGGT |  |
| AsCas12a R2 | CCGGCCTTTTCTGCAGGTCGAGTGCGTTACGCAGTTCCTGGATGTATGC |  |
| FnCas12a F1 | TTGCGGCCGCATGTCAATTTATCAAGA | Restriction enzyme cloning |
| FnCas12a R1 | GTGCGGCCGCTTGGTTATTCCTATTCTG |  |
| d/ddFnCas12a F1 | CTGGTGGACAGCAAATGGGTCGCG | Gibson Assembly |
| dFnCas12a R1 | AGTAAGCTAAATGTCTTTCACCTCTAGCTATACTTAA |  |
| dFnCas12a F2 | AGAGGTGAAAGACATTTAGCTTACTATACTTTGGTAG |  |
| ddFnCas12a R1 | CCAAAATTTAAATCCGCAAAAACCACAATAGC |  |
| ddFnCas12a F2 | TGGTTTTTGCGGATTTAAATTTTGGATTTAAAAGAGGG |  |
| d/ddFnCas12a R2 | CCGGCCTTTTCTGCAGGTCGAGTGC |  |
| LbCas12a F1 | ACTGGTGGACAGCAAATGGGTCGCATGAGCAAACTGGAGAAGTTCA | Gibson Assembly |
| LbCas12a R1 | CTTATACCCTTGTTCTTCGACTTCG |  |
| LbCas12a F2 | CGAAGTCGAAGAACAAGGGTATAAGGT |  |
| LbCas12a R2 | CCGGCCTTTTCTGCAGGTCGAGTGCATGTTTCACGCTGGTCTGCG |  |
| d/ddmgCas12a-1/2 F1 | ACTGGTGGACAGCAAATGGGTCGCG | Gibson Assembly |
| dmgCas12a-1 R1 | TTCGCCGCGGGCAATCCCGATCACA |  |
| dmgCas12a-1 F2 | TGTGATCGGGATTGCCCGCGGCGAA |  |
| ddmgCas12a-1 R1 | CTTAAAACCATAAGAAAGATCTGCCATGGCGA |  |
| ddmgCas12a-1 F2 | ACGCAATTATCGCCATGGCAGATCTTTCTTATGG |  |
| dmgCas12a-2 R1 | TCCGCTCACCGCGAGCAATGCCGAT |  |
| dmgCas12a-2 F2 | TAATCGGCATTGCTCGCGGTGAGCGGAATCT |  |
| ddmgCas12a-2 R1 | CAAATCCGCCATGGCGATTATTGCGTTGTA |  |
| ddmgCas12a-2 F2 | ACAACGCAATAATCGCCATGGCGGATTTGAATTATGG |  |
| d/ddmgCas12a-1/2 R2 | TGGCCGCCGGCCTTTTCTCGAGTGC |  |
| Target DNA #1 (*HsEMX1)* F | GTGGGGACAGAAGGTCTGGAGC | TA cloning |
| Target DNA #1 R | TCTGGGGCTGGAGCTGGATG |  |
| Target DNA #2 (*HsDNMT1)* F | GCTGCTCTCGAACTCCTGGCC |  |
| Target DNA #2 R | GTCAGCCAAGGCCACAAACACC |  |
| Target DNA #3 (*HsCCR5*) F | GGTGGTGGCTGTGTTTGCG |  |
| Target DNA #3 R | CAGAGGCCATCCTCACCCTGAC |  |
| M13 F | GTAAAACGACGGCCAGTGAATTGT | Target DNA amplification from vector |
| M13 R | CAGGAAACAGCTATGACCATGATTACG |  |
| HsCCR5_crRNA1 F1 | GCCAAGCTCTCCATCTAGTGGACAG | HsCCR5 target DNA amplification for T7E1 |
| HsCCR5_crRNA1 R1 | GCAAACACAGCCACCACCCAAG |  |
| HsCCR5_crRNA1 F3 | CCTTCTGGGCTCACTATGCTGCCG |  |
| HsCCR5_crRNA1 R3 | CAGCATTTGCAGAAGCGTTTGGC |  |
|  |  |  |
| HsCCR5_A_SDM_F | GGAATCCTAATAACTCTGCTTCG | Site directed mutagenesis for NTTV PAM |
| HsCCR5_G_SDM_F | GGAATCCTAACAACTCTGCTTCG |  |
| HsCCR5_C_SDM_F | GGAATCCTAAGAACTCTGCTTCG’ |  |
| HsCCR5_SDM_R | CGAGTAGCAGATGACCATG |  |
| HsCCR5_sjIF | GCCAGGACGGTCACCTTTGG | Amplification of PAM variant DNA |
| HsCCR5_sjR2 | CACACTGGCCATATCGGTGGTC |  |
